# Skeletal elements of the penguin eye and their functional and phylogenetic implications (Aves: Sphenisciformes: Spheniscidae)

**DOI:** 10.1002/jmor.21354

**Published:** 2021-05-02

**Authors:** Peter W. Hadden, Dane A. Gerneke, Charles N. J. McGhee, Jie Zhang

**Affiliations:** ^1^ Department of Ophthalmology, New Zealand National Eye Centre, Faculty of Medical and Health Sciences University of Auckland Auckland New Zealand; ^2^ Auckland Bioengineering Institute University of Auckland Auckland New Zealand

**Keywords:** micro‐CT, os opticus, ossicle, penguin, scleral ring

## Abstract

Scleral ossicles and other bony elements are present in the eyes of many vertebrates, including birds. In this study, the skeletal elements present in the penguin eye and orbit were imaged using macro photographs and micro‐computed tomography (micro‐CT), to help elucidate their function and significance. A total of 36 scleral rings and three whole skulls were imaged. King (*Aptenodytes patagonicus*), Fiordland crested (*Eudyptes pachyrhynchus*), Snares crested (*Eudyptes robustus*), royal (*Eudyptes schlegeli*) and yellow‐eyed (*Megadyptes antipodes*) penguins had between 12 and 14 elements in their scleral ring while the gentoo (*Pygoscelis papua*) had 14 and 17; little penguins (*Eudyptula* sp.) consistently had between 10 and 12 elements. All had at least two elements that overlapped, usually totally, each neighbour, and two that were overlapped by each neighbour. The interior structure of all ossicles revealed a lattice‐like arrangement of struts typical of cancellous bone, the whole being surrounded by thick cortical bone. The scleral ring of a 10 week gentoo chick was not completely ossified but rather had multiple small holes within it on micro‐CT. A large os opticus was present in one king penguin but in another bird of the same age and gender there was no such bone. Much smaller accessory bones were found in the posterior pole of one Snares crested and one little penguin. We conclude that the penguin scleral ring not only maintains the shape of the eye but also provides protection and a site of insertion for rectus muscles. However, the extreme variability in the os opticus suggests that it is not essential to normal function.

## Graphical abstract

Micro‐CT of the scleral ring of a Snares crested penguin (*Eudyptes robustus*) surrounding a gentoo penguin (*Pygoscelis papua*) skull. In penguins the scleral ring contains 10–17 overlapping ossicles, maintaining the shape of the eye and providing an attachment for the rectus muscles. Its position within the skull suggests a role in globe protection also. The ossicle ring is 23.3 mm in external diameter and the part of the head imaged is 80 mm long.

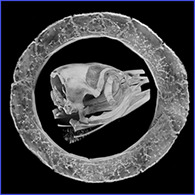

## 1 INTRODUCTION

The sclera is a universal component of the vertebrate eye, forming a firm, pressure‐resistant protective housing for the contents of the globe. In birds, it is reinforced by both bone and cartilage. The bony element consists of a ring of scleral ossicles as well as, unique among vertebrates to some birds, the os opticus (os nervi optici, *Gemminger's ossicle*). The scleral ossicles are a ring of interlocking bones found at the anterior margin of the sclera near the corneo‐scleral junction. They are present in birds, non‐avian dinosaurs, turtles and lizards. In contrast, they are not present in amphibians, snakes, crocodilians or mammals; this difference may be due to secondary loss (Franz‐Odendaal, 2020). The absent or reduced scleral rings of snakes and some other burrowing reptiles may have occurred in association with the general simplification of the body plan, such as the loss of limbs seen in such animals, while scotopic squamates tend to have wider anterior and posterior apertures, resulting in a narrower bony ring (Atkins & Franz‐Odendaal, 2016). The ossicles found in many teleost fish, particularly the more active ones (Franz‐Odendaal, 2008a), may have evolved independently of those found in terrestrial vertebrates (Franz‐Odendaal & Vickaryous, 2006).

Lemmrich (1931) published a description of the scleral ossicles present in European birds, including both his findings and those of previous authors, with whom he did not always agree. We translated this work into English for ease of reference (supplementary online material, File 1). Curtis and Miller (1938), almost contemporaneously and mostly prior to them becoming aware of Lemmrich's work, examined 1404 pairs of scleral rings from North American birds. In birds, the number of individual bones in a scleral ossicle ring ranges from 10 to 20 (Curtis & Miller, 1938) and in some larger species there are said to be air spaces within them (Lemmrich, 1931). There is considerable variation in the steepness and thickness of the ring and pattern of ossicle overlap between different orders of birds (Curtis & Miller, 1938). Tiemeier (1950) and Walls (1963) found that the ossicles of some bird species, generally the larger ones, contain marrow cavities with blood vessels as well as blood and fat cells.

Penguins (Sphenisciformes) are flightless birds that are highly adapted to living and hunting in water. They live almost exclusively in the southern hemisphere, with the exception of the Galapagos penguin (*Spheniscus mendiculus*) that ventures slightly north of the equator. With regard to the scleral ring in penguins, Lemmrich (1931) counted 15 ossicles in one king penguin (*Aptenodytes patagonicus*) specimen and 14–15 in three jackass penguins (*Spheniscus demersus*), noting that others had found a ring with 12 ossicles in a rockhopper penguin (*Catarrhactes* [=*Eudyptes*] *chrysocome*) and 15 in a king penguin. He noted that these numbers were insufficient to draw conclusions from. More recently, the scleral ossicles of the Magellanic penguin (*Spheniscus magellanicus*) were described by Suburo and Scolaro (1990). Lima et al. (2009) also counted 13 and 14 ossicles in a pair of rings from a Magellanic penguin and noted, in this study of 208 Brazilian birds of different orders, that the number of ossicles could vary between each eye of the same individual, although species of the same order were generally similar. Asymmetry was also documented in the chicken (*Gallus gallus*; Franz‐Odendaal, 2008b).

Regarding other bony elements, an os opticus has been said to surround the scleral opening through which the optic nerve passes in some but not all birds (de Queiroz & Good, 1988). Tiemeier (1950), in a study of 639 avian ossa optica, found that they generally have a horseshoe shape, although they sometimes surround the entire optic nerve. He also found marrow cavities containing blood cells, fat and blood vessels in all the os optici that he examined. The aquatic birds that Tiemeier (1950) examined did not have an os opticus; the presence or absence of an os opticus in penguins has not been reported previously, although Lima et al. (2009) did note a small scleral sesamoid bone in a Magellanic penguin.

Given the paucity of information on the morphology, function and significance of the scleral rings and accessory ocular bones in penguins, we aimed to examine the ossicles and other ocular skeletal elements from the five genera of penguins that are available in New Zealand, three of which (*Eudyptula, Megadyptes* and *Pygoscelis*) have not previously been described at all in this regard.

## 2 METHODS

### 2.1 Specimens

**FIGURE 1:**
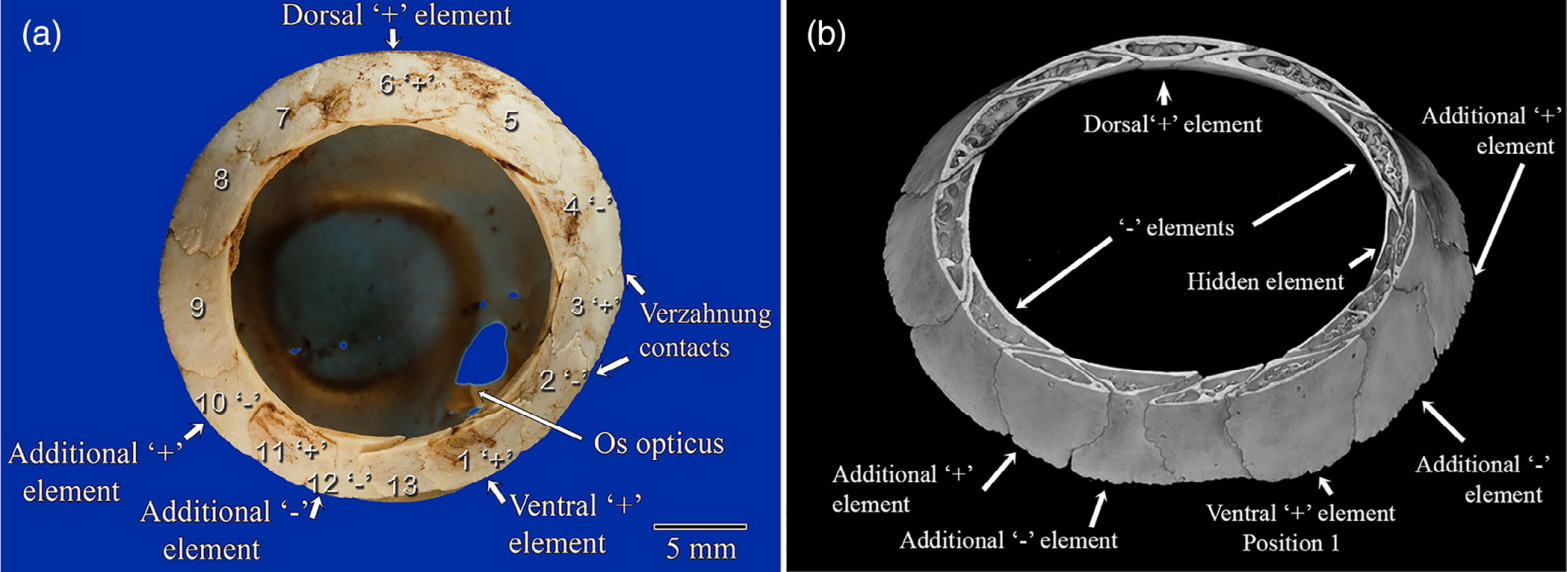
(a) Macro photograph of a scleral ossicle ring of a snares crested penguin (*Eudyptes robustus*, AV 10449A) showing 13 visible ossicles and an os opticus, to demonstrate the most complex and irregular scleral ring in this study. In this specimen alone was the sclera still attached and a small os opticus visible through the central aperture. This is most likely a left eye as the thickest area of the rim (position 5) was on the top right of the photograph, making that the temporodorsal part of the ring. The Lemmrich notation for this ring is 1, 3, 6, 11; 2, 4, 10, 12. There were four ‘+’ elements at positions 1, 3, 6 and 11, making this a type D ring. These comprised two ‘+’ elements, in common positions 1 and 6, and additional ‘+’ elements at positions 11 and 3, with 3 overlapping 2 and 4 on the pupillary side courtesy of two verzahnung contacts. In most specimens, two ‘−’ elements were commonly found at approximately 3 and 9 o'clock. However, in this unusual specimen, the common ‘−’ elements were found at positions 4 and 10, with two additional ‘−’ elements at positions 2 and 12. (b) Micro‐CT image of the same scleral ring in virtual cross section. Note the internal structural detail and the finding of a 14th ossicle hidden under element 3. This was the only such hidden ossicle in the study. It also confirmed that position 4 was a ‘−’ element by being overlapped by both neighbours

A total of 27 scleral rings were obtained from Otago Museum, Dunedin, New Zealand over the period September 2019 to February 2020, under an approval granted by the New Zealand Department of Conservation (68003‐DOA, 28 November 2018). These included four pairs of ossicle rings labelled as being from little penguins (and at the time of collection labelled as *Eudyptula minor*), two single rings, one incomplete, from yellow‐eyed penguins (*Megadyptes antipodes*), five pairs from Fiordland crested penguins (*Eudyptes pachyrhynchus*), two pairs and one single ring from Snares crested penguins (*Eudyptes robustus*) and one pair from a royal penguin (*Eudyptes shlegeli*). For the purposes of some analyses, we grouped Fiordland crested, Snares crested and royal penguins, that is the crested penguins (*Eudyptes*), together. All ossicles were dried, detached from the rest of the body and, judging by the size both of the ring itself and of the corresponding skull, all were from adults. A dried sclera and os opticus was still attached to each ring of one pair of ossicles from one Snares crested penguin (Figure 1). There was no label to identify which side of the head each ossicle came from although ossicles of each pair were labelled A and B. One scleral ring from each pair of scleral rings was examined with micro‐computed tomography (micro‐CT).

A further nine scleral rings were acquired from other sources. One ring from a 10‐week‐old gentoo (*Pygoscelis papua*) chick (G2) and both one ring (K2) and one whole eye (K3) from two different adult king penguins were also scanned. Additionally, the whole head of another adult king (K1), an adult gentoo (G1) and an adult little (L1) penguin were analysed to confirm the position and orientation of the scleral ring within the head, analyse those rings and look for accessory ocular bones. The gentoo and king penguins had been bred and kept in captivity at SEALIFE Kelly Tarlton's Aquarium in Auckland, New Zealand, while the little penguin head (L1) used for micro‐CT was a wild bird that was found dead of natural causes in Auckland. The king penguin stock had been sourced originally from South Georgia and came to SEALIFE Kelly Tarlton's Aquarium via SeaWorld San Diego, California, USA. The gentoo penguins also were of South Georgian descent, although the proximate source was Edinburgh Zoo, Scotland, UK. None of these specimens had been dried or macerated; rather, following death they had been stored frozen and then imaged either immediately after defrosting or, in the case of the complete heads, while still frozen, except in the one case noted below.

### 2.2 Macro imaging

Macro photographic images were taken using a purpose‐constructed macro imaging system using a Canon EOS Kiss i7 and Canon macro EF 100 mm f/2.8 USM lens in manual mode (Canon 30‐2 Shimomaruko 3‐chome, Ota‐ku, Tokyo Japan). Single field images of each ossicle were recorded at the same lens focus so are at the same magnification. The ossicles were imaged tilted so that the outer ring of the ossicle was orientated to be parallel to the sensor plane to match the orientation of the 3D images from micro‐CT data sets. Processing of the RAW files and conversion to .tiff files was undertaken using the RAW converter in Adobe Photoshop CS 6 (San Jose, CA, RRID:SCR_014199), the same settings being used for all.

### 2.3 Micro‐computed tomography

Micro‐CT data acquisitions of ossicles and the little penguin head were conducted using a Bruker Skyscan 1172, and for the gentoo head we used a Bruker Skyscan 1272 instrument (Kontich, Belgium). The images were reconstructed using InstaRecon CBR Server Premium 15K (InstaRecon Inc., Champaign, IL) and subsequently visualised in 3D using CTVox V 3.3 (Bruker, Kontich, Belgium). Data View V 1.5.4.0 (Bruker, Kontich, Belgium) was used to adjust rotational and tilt orientation to give comparable views of all samples. Analysis was done using CTAnal V1.18.4.0 (Bruker, Kontich, Belgium). Additional editing was done using Adobe Photoshop CC V19.1.3 (San Jose, CA, RRID:SCR_014199). Micro‐CT data of the king penguin head was acquired with the following North Star CT instrument parameters: X25 CT system NSI (North Star Imaging, Rogers, MN). The software used for analysis was Geomagic Design X (Geomagic Inc., Morrisville, NC). Further information on the scanning parameters is presented in Table 1.

**TABLE 1:** The imaging conditions used for micro‐CT of the ossicles, penguin heads and the enucleated king penguin eye

Sample	CT	kV	μA	Filter Al.	Exposure time (ms)	μm pixel resolution	Rotation step (°)	Rotation (°)	Number of fields	Scan time (h)	Frame Ave.	Random movement
All ossicles												
Ossicles	1172	78	128	0.25	400–1050	10.02–13.54	0.3–0.4	360	1–4	1.75–3.0	2	4
Heads												
Little	1172	100	100	1.0	1100	27.09	0.5	360	6	6.25	2	6
Gentoo	1272	100	100	0.5	1500	20.0	0.6	360	18	15.5	2	6
King	X25	150	100	None	‐	170.8	Helical	360 × 12	‐	0.25	‐	‐
Eye												
King eye	1172	94	106	None	800	10.15	0.5	360	4	4.2	2	‐

After scanning of the whole head of the little penguin L1, it was defrosted and the right eye was enucleated to improve penetration of fixative and iodine to deeper structures of the orbit. The rest of the head was fixed in 1% formaldehyde and 1.25% glutaraldehyde, dehydrated using an ethanol gradient (30, 50, 70% for 6 days each), changing the solution every 3 days, stained with alcoholic potassium triiodide (IKI) solution 0.75% for 14 days, and briefly washed in 70% alcohol. It was re‐scanned using the same parameters as previously except 1550 ms 1.0 mm aluminium filter, 4x random movement over 360°, scan time 7.5 h. The enucleated eye was not further analysed in this study.

An enucleated eye from king penguin K3 was fixed for 24 h using 3% formalin in phosphate‐buffered saline (PBS), flushing the vitreous cavity with the fixative using a 28 gauge needle. The vitreous cavity was then flushed four times with PBS before repeating the flush using 1.5% aqueous IKI and the eye was stored in the same solution for 3 days. The vitreous cavity was then flushed twice with water and left in fresh water for 24 h. Due to unsatisfactory image quality, repeat staining and flushing of the vitreous cavity with 1.5% aqueous IKI for 4 days was required and the eye was then scanned using the parameters in Table 1.

It is important to note that, when visualising both macro photographs and 3D micro‐CT data, perspective distortion alters interpretation. Shorter focal length wide angle camera lenses distort close objects and make distant objects appear much further away than they really are, while longer telephoto lenses compress the real distance of features in the field of view. In the same way, 3D visualisation programmes allow perspective to be changed in micro‐CT. In CTVox, once the ‘camera angle’ is greater than 70° severe distortion results (Appendix 2). Therefore, to enable a valid comparison and reproducibility we maintained a 10° ‘camera angle’ for all micro‐CT samples.

### 2.4 Description of scleral ossicles using Lemmrich's convention

We elected to use that notation devised by Lemmrich (1931) to describe the positions of the ossicles within the scleral ring (Figure 1). Lemmrich (1931) divided avian scleral rings into two basic types. In Type A, the most dorsal element overlaps both neighbours and another, approximately opposite, ventral element also overlaps both its neighbours. There are also two elements, at approximately 90° to these, that are overlapped by both neighbours. He called overlapping ossicles ‘+’ elements and overlapped elements minus elements; for the latter he used the symbol ‘O’ in his drawings but we have elected to use the mathematical minus symbol ‘−’ for clarity. He termed these ‘+’ and ‘−’ elements ‘ausgezeichneten’. In the less common Type B rings there is only one ventral ‘+’ element that overlaps each neighbour. Lemmrich (1931) then assigned the ventral ‘+’ element position 1 and numbered the remaining elements sequentially, proceeding in a posterior‐dorsal direction from 1. He listed the position of each ‘+’ element, separating the numbers using a comma and, following a semicolon, listed the numerical position of each ‘−’ element.

Some elements do not completely overlap the entire width of their neighbour but instead only overlap a portion thereof and are themselves overlapped over the other portion. These were called ‘verzahnung’ contacts by Lemmrich (1931). In such cases, Curtis and Miller (1938) arbitrarily considered the overlap at the inner or pupillary border to be the one that established the status of the plate and we have used this convention. The latter also reported that many loons have a ring with three ‘+’ and three ‘−’ elements and occasional birds have four of each. We felt it would be appropriate, given that a Type A ring is the most common and Type B the second most common, to call a ring with three such elements Type C and the even rarer ring, with four of each, a Type D ring.

### 2.5 Measurement and statistical analysis of scleral ring geometry

The number of ossicles in each genus (*Eudyptula*, *Eudyptes*, *Aptenodytes* and *Pygoscelis*, excluding *Megadyptes* due to a sample size of 1 was analysed using one‐way ANOVA in SPSS (International Business Machines Corporation [IBM], Armonk, NY). A test of homogeneity of variances returned a significant difference due to the large variation in sample sizes. The Games‐Howell post hoc test was performed to find out significant differences between two groups without assuming equal variances. A *p*‐value of <.05 indicates a significant difference between groups.

The size and shape of each ring imaged using micro‐CT were analysed by DAG on reconstructed images using CTAn V 1.18.4.0 software (Bruker, Kontich, Belgium). The internal and external diameters were measured radially in the x and y axes to minimise stepped pixel edges occurring at intermediate angles and the angle made between the plane of the anterior edge of the ring and the slope of each ring at its thinnest and thickest point was measured. We refer to this angle henceforth as ‘steepness’. Measurement error in grey level images was 1–2% pixels. The mean difference in pixel width measurements between the grey level transaxial plane and the same binary transaxial plane was 2.2% (minimum 0%, maximum 3.5%). Those rings with thicker, denser edges were better defined and easier to confidently select the measurement points. All analyses were undertaken by the same person.

The shape and size of the scleral rings as imaged by micro‐CT were measured in 2D and 3D using CTAn V 1.18.4.0 software (Bruker, Kontich, Belgium). Data View V 1.5.4.0 (Bruker, Kontich, Belgium) was used to re‐orientate all rings for consistency of measurements. Because not all rings were imaged using micro‐CT and because the micro‐CT images of the scleral rings obtained using the whole head were not of sufficiently high resolution to measure accurately using the above methods, we were only able to statistically compare the scleral rings from three genera (*Eudyptula*, *Eudyptes* and *Aptenodytes*), using one‐way ANOVA in SPSS. A test of homogeneity of variances was performed. Where there was not a significant difference, the Tukey post hoc test was performed and where there was a significant difference the Games‐Howell post hoc test, to determine any significant differences between the genera. A *p*‐value of <.05 indicates a significant difference.

## 3 RESULTS

**FIGURE 2:**
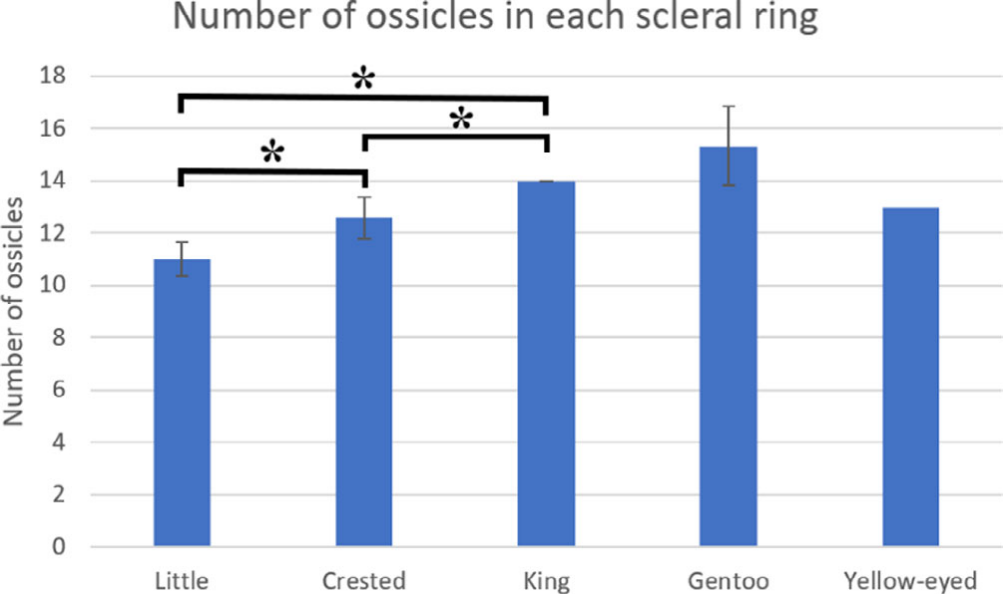
Average number of ossicles in each scleral ring by genus. Bars represent mean ± *SD*. *p < .05. The scleral ring from the yellow‐eyed penguin was excluded from analysis due to a sample size of 1. Therefore it has no *SD* bars. Samples sizes were 10, 17, 4, 3 and 1, respectively

Little penguins had between 10 and 12 ossicles per ring (11 ± 0.6, mean ± *SD*, *n* = 10), crested penguins had 12–14 ossicles (12.6 ± 0.8, *n* = 10), all king penguins had 14 ossicles (14 ± 0, *n* = 4), gentoo penguins had 14, 15 and 17 ossicles (15.3 ± 1.5, *n* = 3) and the one intact yellow‐eyed penguin ring had 13 ossicles (Figure 2). One‐way ANOVA analysis of the first four groups found a statistically significant difference between groups. The yellow‐eyed penguin ring was excluded from analysis due to having a sample size of one. Post hoc tests found significant differences between little and crested (*p* < .001), little and king (*p* < .001) and crested and king (*p* < .001). All but two ossicle rings examined in this study were Type A rings, with two overlapping ‘+’ and two underlapping ‘−’ elements. Those exceptions were a Type C ring from a Fiordland crested penguin and a Type D ring from a Snares crested penguin (Figure 1). Please see supplementary online material Appendix 1 for a description of each scleral ossicle ring.

**FIGURE 3:**
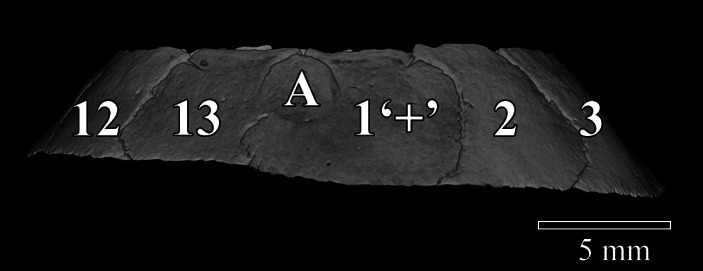
3D visualisation using micro‐CT of a scleral ring from a yellow‐eyed penguin (*Megadyptes antipodes*, AV 1181C), viewed from the ventral aspect. Between neighbouring ossicles, the zone of contact was slightly arcuate. One side of the ring had a wider rim than the other, making this a left eye. Unique to this specimen was a small bone (marked ‘A’) fused to and overlapped both neighbours at positions 1 and 13. It was not counted as a separate ossicle due to the fusion. It was also difficult to define the ventral ‘+’ element, as the small, fused bone was on the pupillary side of the ring. The ossicle at position 1 was arbitrarily labelled a ‘+’ element as it overlapped the ossicles at positions 2 and 13

The asymmetry of the ossicle ring (Figure 3) enabled determination of the orientation of ring and the side of the skull from which it came. In all the little penguins examined, the dorsal ‘+’ element was slightly smaller than the ventral but subjectively there was little variation in ossicle size in little penguins. Some Snares and Fiordland crested penguins had very small ‘+’ elements, such that the two ossicles beneath it were in contact with each other. One Snares crested penguin (AV10449) had a symmetric Type A ring in the right eye but in the left eye the irregular Type D ring shown in Figure 1, with two verzahnung contacts and an extra ‘hidden’ ossicle to which we did not assign a position. There was similar verzahnung contacts in two of three rings of the gentoo penguins and in one king penguin ring. In general, the surface area of different ossicles from the same scleral ring was more variable in Fiordland crested, Snares crested and yellow‐eyed penguins than in the other species. A scleral ring from a yellow‐eyed penguin (AV1181C) was found to contain a small bone that was partially fused to each of its neighbouring bones (Figure 3). We elected not to count this as an ossicle, given the fusion.

**FIGURE 4:**
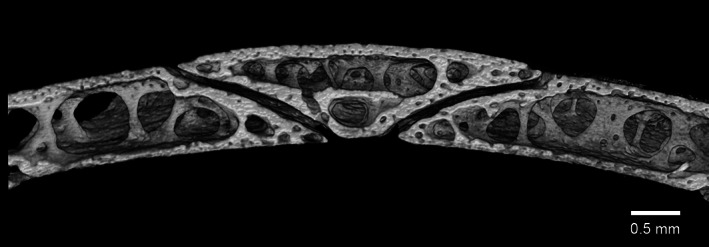
Micro‐CT of the ossicle ring of a gentoo penguin chick (*Pygoscelis papua*, G2) showing one ossicle overlapping two others. There are spaces within the still developing bone of the chick, not present in any adult examined, yet the scleral ring is clearly already present at 10 weeks

**FIGURE 5:**
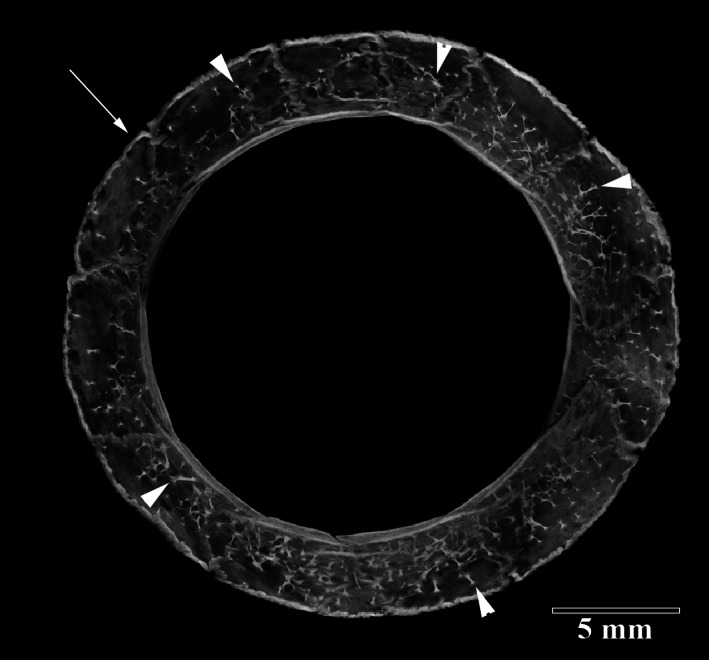
Micro‐CT maximum intensity projection image of the scleral ossicle ring of the right eye of a Snares crested penguin (*Eudyptes robustus*, AV 1178A). The interior of ossicles contained multiple internal struts (arrowheads) instead of solid bone. The external surface rim of the scleral ring consisted of more solid bone (arrow)

**FIGURE 6:**
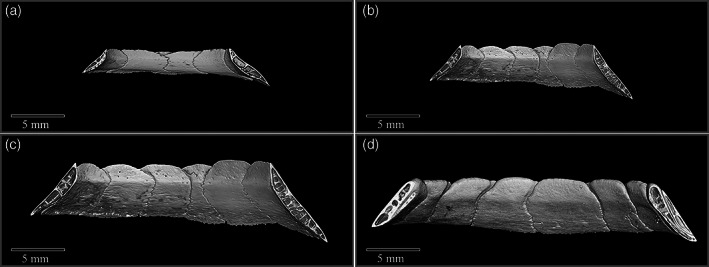
Midline cut away micro‐CT image to show the curvature of the inner and outer surfaces of the scleral ring in two little penguins (a and b, likely to be *Eudyptula novaehollandiae*, AV 958A and AV 989A), one Snares penguin (*Eudyptes robustus*, AV 1181A) and one king penguin (*Aptenodytes patagonicus*, K3). The outer surface appeared almost flat but the inner was concave inwards. The curvature of the scleral ring did vary but, excluding size differences, without digital analysis there did not appear to be greater variation in shape between species and genera than between individuals of the same species, as demonstrated by the obvious curvature difference between (a) and (b), both *Eudyptula*, but not between (b) and (c)

Micro‐CT showed that, in all species examined, each ossicle made a large overlapping contact with its neighbour and the edge of the overlapping ossicle tended to be somewhat arcuate, being roundest in the little penguin and most angular in the king (supplementary online material, Appendix 1). The internal structure of all ossicles was not solid but rather contained multiple bony struts, although the whole was surrounded by a solid external bony surface (Figures 4 and 5). Generally, also the widest part of the ring tended to be flatter and the narrowest part of the ring steeper. The external surface of the scleral ring was only minimally curved in this series. In contrast, the internal surface was convex inwards, particularly anteriorly (Figure 6).

The size of the scleral ring, as measured by volume, surface area, diameter and both x and y centroid, was largest in king penguin scleral rings, then crested penguins and smallest in little penguins. The yellow‐eyed penguin ring was excluded from analysis due to having a sample size of one. One‐way ANOVA analysis found a statistically significant difference between these groups for all these parameters. These results were expected as they reflect the relative size of each bird. However, the z centroid was statistically larger than the others in crested penguins, indicating that their scleral rings are deeper from anterior to posterior (Table 2). Shape analysis revealed that king penguin scleral rings possess a significantly greater surface convexity index, a lower eccentricity and a significantly steeper ring at the thinnest point than the other two genera. Little penguins, conversely, had a significantly less steep ring at the thickest point than both king and crested penguins (Table 3).

**TABLE 2:** As one would expect, penguins of the genus *Eudyptula* have the smallest scleral rings, with the rings of crested penguins (*Eudyptes*) being larger while those of king penguins (*Aptenodytes patagonicus*) are larger again

	Number	Mean	*SD*	Std error	*p*‐Value, one‐way ANOVA	*p*‐Value, Tukey HSD post hoc analysis		
						Versus little	Versus crested	Versus king
**2D object surface (mm** ^**2**^ **)**								
Little	6	1158.4	139.0	56.7	<.001		<.001	<.001
Crested	10	2602.7	412.2	130.4		<.001		<.001
King	2	4326.1	10.9	7.7		<.001	<.001	
Yellow‐eyed	1	2182.3						
**2D object volume (mm** ^**3**^ **)**								
Little	6	69.3	19.9	8.1	<.001		<.001	.018
Crested	10	156.7	43.7	13.8		<.001		.014
King	2	269.8	19.8	14.0		.018	.014	
Yellow‐eyed	1	140.0						
**2D centroid x axis (mm)**								
Little	6	10.1	0.7	0.3	<.001		<.001	<.001
Crested	10	14.1	1.0	0.3		<.001		<.001
King	2	18.8	0.6	0.4		<.001	<.001	
Yellow‐eyed	1	13.1						
**2D centroid y axis (mm)**								
Little	6	9.8	1.3	0.5	<.001		<.001	<.001
Crested	10	13.3	0.8	0.2		<.001		.004
King	2	16.2	0.8	0.6		<.001	.004	
Yellow‐eyed	1	11.8						
**2D centroid z axis (mm)**								
Little	6	5.6	2.1	0.9	.195			
Crested	10	8.2	3.7	1.2				
King	2	4.8	0.8	0.6				
Yellow‐eyed	1	7.9						
**3D object volume (mm** ^**3**^ **)**								
Little	6	69.0	19.9	8.1	<.001		<.001	<.001
Crested	10	156.5	43.3	13.7		<.001		.003
King	2	268.7	19.8	14.0		<.001	.003	
Yellow‐eyed	1	140.1						
**3D object surface (mm** ^**2**^ **)**								
Little	6	879.4	107.5	43.9	<.001		<.001	<.001
Crested	10	1978.9	316.7	100.1		<.001		<.001
King	2	3320.6	12.4	8.8		<.001	<.001	
Yellow‐eyed	1	1640.8						
**3D centroid x axis (mm)**								
Little	6	10.1	0.7	0.3	<.001		<.001	<.001
Crested	10	14.1	1.0	0.3		<.001		<.001
King	2	18.8	0.6	0.4		<.001	<.001	
Yellow‐eyed	1	13.1						
**3D centroid y axis (mm)**								
Little	6	9.8	1.3	0.5	<.001		<.001	<.001
Crested	10	13.3	0.8	0.2		<.001		<.001
King	2	16.2	0.8	0.6		<.001	<.001	
Yellow‐eyed	1	11.8						
**3D centroid z axis (mm)**								
Little	6	5.6	2.1	0.9	.311			
Crested	10	8.2	3.7	1.2				
King	2	4.8	0.8	0.6				
Yellow‐eyed	1	4.1						
**Inside diameter x axis (mm)**								
Little	6	11.9	0.3	0.1	<.001		<.001	<.001
Crested	10	17.2	0.8	0.2		<.001		<.001
King	2	22.4	0.2	0.1		<.001	<.001	
Yellow‐eyed	1	16.1						
**Outside diameter x axis (mm)**								
Little	6	17.8	0.7	0.3	<.001		<.001	<.001
Crested	10	25.0	1.0	0.3		<.001		<.001
King	2	30.9	0.9	0.7		<.001	<.001	
Yellow‐eyed	1	23.9						
**Inside diameter y axis (mm)**								
Little	6	11.8	0.4	0.2	<.001		<.001	<.001
Crested	10	17.3	0.8	0.3		<.001		<.001
King	2	22.4	0.2	0.1		<.001	<.001	
Yellow‐eyed	1	16.6						
**Outside diameter y axis (mm)**								
Little	6	17.4	0.8	0.3	<.001		<.001	<.001
Crested	10	24.0	1.2	0.4		<.001		<.001
King	2	31.0	1.1	0.8		<.001	<.001	
Yellow‐eyed	1	24.1						

*Note*: This size difference is statistically significant for all parameters except the z centroid, a measure of the size of the ring in its z axis (from the side closest to the cornea to that closest to the posterior pole), which is greatest in crested penguins, although the difference is not statistically significant. The scleral ring of the yellow‐eyed penguin (*Megadyptes antipodes*) could not be analysed statistically due to a sample size of 1, although seemed to be of similar size to those of crested penguins.

**TABLE 3:** There are significant differences in shape of the penguin ossicle ring between the genera *Eudyptula*, *Eudyptes* and *Aptenodytes*

	Number	Mean	*SD*	Std error	*p*‐Value, one‐way ANOVA	*p*‐Value, Tukey HSD post hoc analysis		
						Versus little	Versus crested	Versus king
**2D mean eccentricity**								
Little	6	0.5657	0.06034	0.02463	.002		.979	.003
Crested	10	0.5707	0.04386	0.01387		.979		.002
king	2	0.403	0.03816	0.02698		.003	.002	
Yellow‐eyed	1	0.64396						
**2D mean surface convexity index**								
Little	6	−1.189	1.15489	0.47148	.004		.542	.003
Crested	10	−0.5874	0.48363	0.15294		.542		.008
King	2	2.3833	2.96319	2.09529		.003	.008	
Yellow‐eyed	1	−0.79577						
**Steepness at thinnest point (°)**								
Little	6	44.2833	1.51052	0.61667	.138			
Crested	10	43.15	3.52365	1.11428				
King	2	47.85	0.35355	0.25				
Yellow‐eyed	1	44.05						
**Steepness at thickest point (°)**								
Little	6	46.0167	2.20401	0.89978	.018		.018	.126
Crested	10	51.32	3.89353	1.23124		.018		.993
King	2	51.6	0.14142	0.1		.126	.993	
Yellow‐eyed	1	49.5833						

*Note*: In particular, the king penguin (*Aptenodytes patagonicus*) has more convex and less eccentric rings and the ring is steeper than others at the thinnest point. At the thickest point, however, there is no statistically significant difference between the steepness of the scleral ring in *Eudyptes* and king penguins, but both are steeper at this point than are the rings of penguins from the genus *Eudyptula*.

**FIGURE 7:**
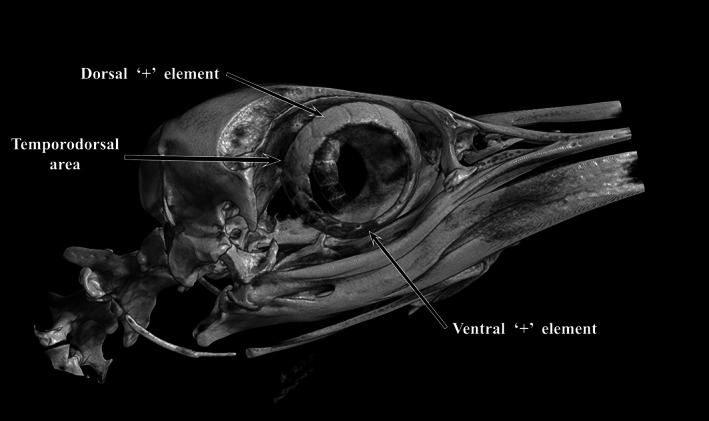
Micro‐CT image of the whole head of a king penguin (*Aptenodytes patagonicus*, K1) showing a dorsal ‘+’ element at 12 o'clock. The temporo‐dorsal area was the widest part of the scleral ring. It was also apparent that the ventral ‘+’ element was further rostral than the dorsal ‘+’ element

**FIGURE 8:**
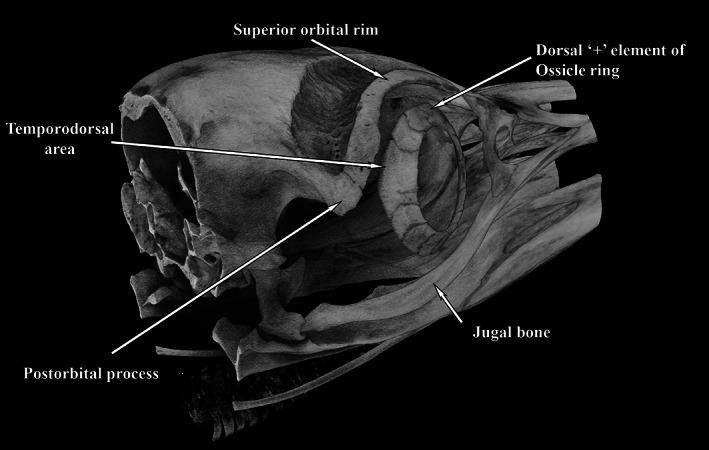
Micro‐CT image of the whole head of an adult gentoo penguin (*Pygoscelis papua*, G1) showing a dorsal ‘+’ element at 12 o'clock. The area between the postorbital process and the jugal bone contains the thick musculus adductor mandibulae externus, whose bulk would protect the globe in that area. The part of the globe least protected by the bony orbit can be seen to be the dorsal and temporo‐dorsal area, where the ring was thickest. Length of head imaged = 80 mm

Micro‐CT of the whole heads G1, K1 and L1 (Figure 7, king K1, Figure 8, gentoo G1) demonstrated the presence of a dorsal ‘+’ element at approximately 12 o'clock in all. It also showed that the temporo‐dorsal element is the widest part of the ossicle, confirming the findings of Lemmrich (1931) and validating our orientation of those ossicles that were not in an intact head. Excluding the area between the postorbital process and the posterior jugal bone, protected by the bulk of the thick musculus adductor mandibulae externus, the part of the globe least protected by the bony orbit appeared to be the dorsal and temporo‐dorsal area, where the scleral ring was thickest (Figure 8).

**FIGURE 9:**
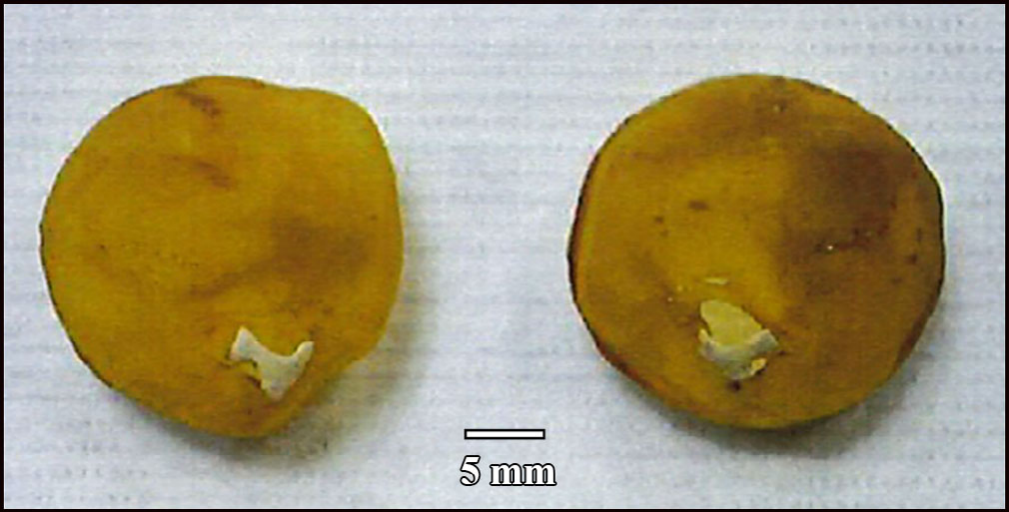
The small optic nerve head ossification attached to the remnants of the cartilaginous scleral cup in each eye of a Snares crested penguin (*Eudyptes robustus*, AV 10449 A and B)

**FIGURE 10:**
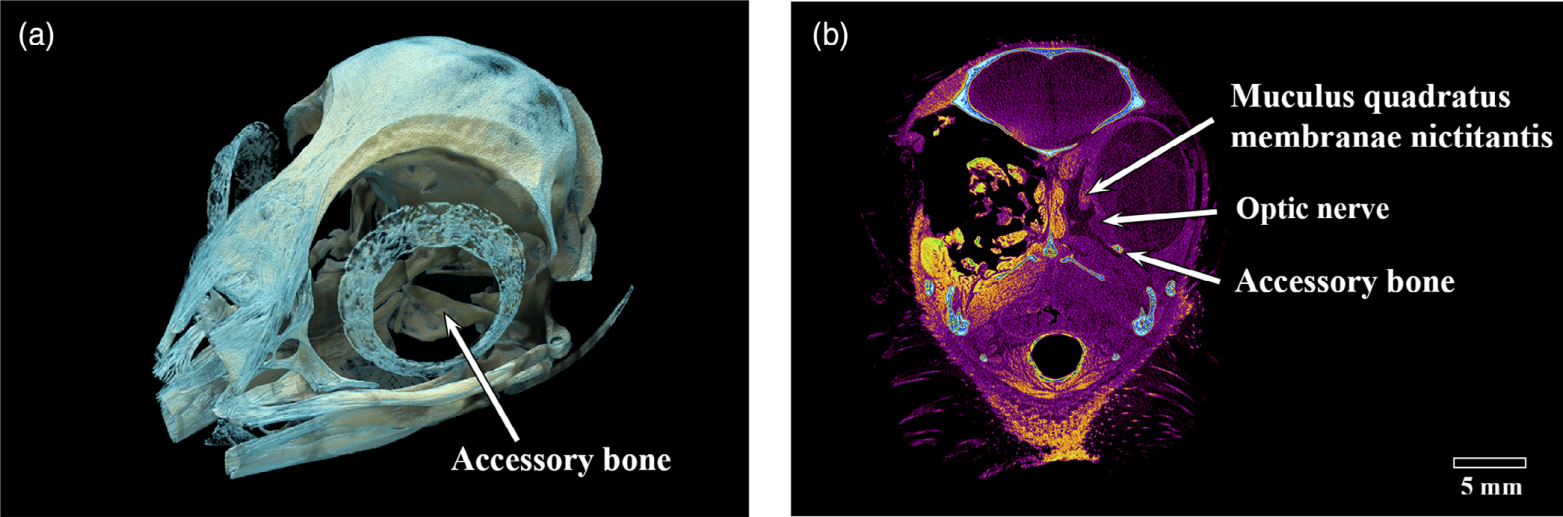
Micro‐CT images of the whole head of an adult little penguin (*Eudyptula minor*, L1). (a) An accessory bone could be identified deep to the centre of the scleral ring. (b) When soft tissues were stained, the accessory bone was adjacent to the scleral wall and just ventral to the optic nerve

**FIGURE 11:**
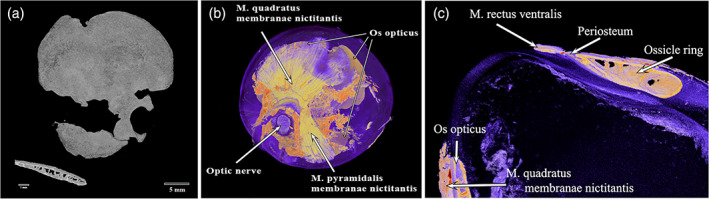
Micro‐CT images of the whole left eye of an adult king penguin (*Aptenodytes patagonicus*, K3). (a) A large os opticus viewed through the scleral ring. The lower left picture showed a virtual cross section of the os opticus, which contained struts surrounded by solid bone. (b) After staining, the purple to pale yellow os opticus appeared to surround the optic nerve and underlie the musculus (m.) quadratus membranae nictitantis and M. pyramidalis membranae nictitantis (yellow‐green), muscles that move the nictitating membrane. The bright orange was connective tissue. (c) M. rectus ventralis was seen to insert at the posterior edge of the scleral ring. Again the os opticus was seen to underlie almost exactly M. quadratus membranae nictitantis

A small bone was found still attached to the remnants of the cartilaginous scleral cup in each eye of a Snares crested penguin (Figure 9). It did not have the horseshoe shape of the os opticus as described by Tiemeier (1950), being much smaller than he described and not large enough to surround the optic nerve, but rather fitted within the opening in the sclera for the optic nerve. It had three arms. An even smaller accessory bone in each eye was also identified on micro‐CT of the little penguin L1, immediately ventral and slightly anterior to the optic nerve (Figure 10). It was small, with neither a horseshoe shape nor arms. Micro‐CT of the king penguin K3 revealed a much larger bone, more similar to Tiemeier's description of an os opticus, which roughly corresponded to where the muscles that move the nictitating membrane (musculi quadratus membranae nictitantis et pyramidalis membranae nictitantis) were situated (Figure 11). However, no accessory bone of any size could be found on micro‐CT of the king (K1) and gentoo (G1) penguin skulls.

## 4 DISCUSSION

De Queiroz and Good (1988) felt that 14–15 scleral ossicles would appear to be basal for neognath birds. These were also the most common numbers of ossicles found by Curtis and Miller (1938). *Aptenodytes*, that is emperor (*A. forsteri*) and king penguins, are considered by some to be the most basal penguins (Baker et al., 2006) and our finding that all four scleral rings from this genus had 14 ossicles does concord with this.

The little penguins in this study had the smallest number of ossicles in each ring, statistically different from crested and king penguins. Among these three groups, little penguins both had the smallest rings and are the smallest birds (Scofield & Stephenson, 2013; Shirihai, 2007), followed by crested penguins and then the larger king penguins, which seemed to suggest a body and scleral ring size association with number of ossicles, consistent with findings in other birds (Franz‐Odendaal, 2020). However, the gentoo penguins in this series had either as many or more ossicles (14–17) as king penguins despite both a slightly smaller ring and body size. Curtis and Miller (1938) also found no correlation between the size of the ring and the number of ossicles in North American birds.

All little penguin museum specimens were labelled as *Eudytula minor*. Recent genetic evidence suggests that those from the Otago Region may well have been *Eudyptula novaehollandiae*, which also inhabit southern Australia and there are often called fairy penguins, although the other (L1) was from Auckland and therefore almost certainly was *E. minor* (Grosser et al., 2017). These two species diverged about 3MYA but, for consistency and because in Otago the name ‘fairy penguin’ was not in common use at the time they were labelled, the name little penguin was used throughout to refer to members of either species. There was no obvious difference between the scleral rings in this small group. Conversely, the royal penguin is considered genetically indistinguishable from the macaroni (*Eudyptes chrysolophus*) (Cole et al., 2019).

Verzahnung contacts are more common in some bird orders than in others, varying from only one case in Columbiformes to 81.5% in woodpeckers (Curtis & Miller, 1938); they reported tendency for them to occur in definite locations in the ring, depending on the species. They appear to be uncommon in penguins, as out of 36 complete scleral rings in our study there were only seven such contacts, but the small numbers made it hard for us to draw conclusions regarding location. Lemmrich (1931) noted that some authors who preceded him found an uneven number of ‘+’ and ‘−’ elements to be a common occurrence. He suggested that they might have been mistaken, except for a few cases where one ossicle was interlocked with its neighbour due to a verzahnung contact, making the determination of ‘+’ or ‘−’ difficult. We had to apply Curtis and Miller's (1938) convention to resolve this situation in only four cases (Snares AV 10449B, king K2, gentoo G1 right eye and yellow‐eyed AV 1181C). The curvature of the scleral ring did vary, but to the naked eye, there did not appear to be greater variation between species and genera than between individuals of the same genus (Figure 6). However, shape analysis confirmed significant differences between genera and, unexpectedly, showed that the scleral rings of crested penguins have a greater anterior to posterior length than the otherwise larger rings of king penguins. These differences may allow identification by genus of penguins from the size and morphology of the scleral ring alone, using micro‐CT and computer analysis. It is possible, however, that the rings may have been distorted during their collection and storage.

It is difficult to understand why little, king and royal (macaroni) penguins appear to have a noticeably more regular arrangement of ossicles and ossicles of a more regular size than do other crested penguins. Possibly the latter are more subject to inbreeding depression and genetic drift within their smaller populations and therefore have a scleral ring that deviates more from the family norm, but the numbers in this study are small. Lemmrich (1931) suggested that domesticated birds show greater variability in the scleral ring but this was disputed by Curtis and Miller (1938). Given the variability in the penguins of this series, although Suburo and Scolaro (1990) found that there were consistently 13 ossicles in each of eight rings from Magellanic penguins, we thought it very unlikely that all such penguins have 13 ossicles and this is supported by the finding of Lima et al. (2009) of 13 and 14 ossicles in a pair of rings from one Magellanic penguin.

Although with practice we found it easy to tell whether a scleral ring came from a little, crested or king/gentoo penguin, it was not possible to subjectively differentiate scleral rings from different species within the same genus. This corresponds to findings in other avian genera. Warheit et al. (1989) commented that there was significant variation in the scleral ring between genera but not between species in pelicans (Pelecaniformes) and de Queiroz & Good (1988) noted a similar lack of intrageneric variability.

The function of scleral ossicles is a subject of debate. It may be that it is simply a vestigial structure, but Curtis and Miller (1938) felt that this is unlikely to be true given the considerable development of the ring in birds. They have been postulated to play a role in maintaining the shape of non‐spherical eyes. This is supported by the finding that it tends to be particularly robust in birds such as owls which have very aspheric eyes (Franz‐Odendaal & Vickaryous, 2006) and the fact that dinosaurs' nearest living relatives, crocodilians, do not have a scleral ring and have a spherical eye (Ruiz et al., 2015). Further studies to look at the strength of the ring and correlate that to the size and asphericity of the eye would be helpful.

The scleral ring has also been postulated to serve as an attachment for the ciliary muscle and have a role in accommodation, given that the ossicles are capable of movement against each other (Slonaker, 1918), and to prevent distortion of the globe during the extensive accommodation seen in some diving birds (Walls, 1963). In order to be emmetropic both above and below water, the great cormorant (*Phalacrocorax carbo sinensis*) and many other pursuit‐diving birds require over 60 dioptres of lenticular accommodation to compensate for the almost total loss of corneal refractive power when underwater (Katzir & cHowland, 2003). By contrast, the chicken (*Gallus gallus domesticus*) has an accommodative amplitude in the order of 10–20 dioptres (Sivak et al., 1986) and both it and the pigeon (*Columba livia*) have been shown to rely more on corneal as opposed to lenticular accommodation (Pardue & Sivak, 1997). Absence of a scleral ring in animals such as snakes and placental mammals has been suggested to reflect a period of nocturnal evolution when accommodation was not essential (Atkins & Franz‐Odendaal, 2016). The penguin does not require as extreme lenticular accommodation because of a relatively flat cornea (Howland & Sivak, 1984) and the refractive status of the penguin on land has been the subject of some debate. However, the presence of a substantial scleral ring and the finding in the Adélie penguin (*Pygoscelis adeliae*) of an iris sphincter muscle similar to those in diving birds (Sivak & Vrablic, 1979) support the argument of a significant accommodative ability.

In our study, micro‐CT of the iodine‐stained enucleated king penguin eye demonstrated a rectus muscle inserting directly into the periosteum at the posterior edge of the ring (Figure 11c). We therefore suggest that another function of the ossicle ring is to provide a solid anchor for these muscles which rotate the globe, a function that was considered by Lemmrich (1931) but which he could not support.

Ichthyosaurs, extinct Mesozoic reptilian relatives of dinosaurs, were able to dive to great depths (almost certainly to depths greater than 500 m) and had both extremely thick ossicles and huge eyes. They may have required such large eyes to gather more light at depth (Motani et al., 1999). Perhaps also it was the size of the eye that demanded a strong and larger ossicle. Additionally, a stronger scleral ring may reduce deformation of the globe when swimming at speed and there does appear to be a correlation between fast‐moving teleost fish and birds and a larger ring (Fischer & Schoenemann, 2019). We feel that the explanation that thicker ossicles allowed deeper diving by withstanding greater compression (Schwab, 2002) is not likely to be important because the eye does not contain any easily compressible gases, although emperor penguins have been recorded diving to over 500 m (Wienecke et al., 2007) and seawater is slightly compressible at depth (Rodriguez & Millero, 2013). Compressibility could therefore be a consideration in the larger, deeper diving birds.

The widest part of the scleral ring corresponds to that part of the eye that is most exposed and left unprotected by surrounding skull structures. This was most obvious in the head of the gentoo penguin (G1, Figure 7), where it would appear that the ring and contiguous bony orbit combine to constitute a more all‐encompassing bony case for the globe. The argument that the ring is protective was considered by Lemmrich (1931) and is supported by Curtis and Miller's (1938) observation that diving birds have more bony rings and rapid fliers steeper rings.

Finally, the scleral ring would appear to ossify early in life, which agrees with the work of Tiemeier (1950) and Lemmrich (1931), although in this study they were not fully ossified at 10 weeks, at least in the gentoo penguin (Figure 4). Franz‐Odendaal (2008b) has shown that, in chicken embryos, ossification commences at stage 36 (day 10). Ossification is preceded by the formation of a conjunctival papilla, a process with inbuilt redundancy such that even if a papilla is removed the ring will still form, albeit over a longer period, and interrupting the process later merely means that the remaining ossicles enlarge to produce a complete ring but with a lesser number of elements (Franz‐Odendaal, 2008b; Jourdeuil & Franz‐Odendaal, 2017). Regarding the os opticus, this series showed that it is not a consistent feature of the penguin eye, as has been found to be the case in some other birds (Tiemeier, 1950). Tiemeier (1950) felt that the os opticus might have a role in ‘(a) protection of the eye against shock; (b) maintenance of rigidity of the eyeball; and (c) protection of the central segment of the optic‐nerve head’. The fact that the ossified area corresponds to the insertion point of muscles that move the nictitating membrane (Figure 10), as well as surrounds the optic nerve, suggests that if present it may also have some role in force transmission by these muscles. However, given that in our study there were two king penguins, both male and both euthanized for health reasons aged 26 years after a life in captivity, one with a large os opticus and the other with none, we suggest that the os opticus does not play an essential role in the function of the eye.

We also found smaller ossifications in the posterior pole of one little and one Snares crested penguin but micro‐CT definitively excluded their presence in one gentoo and one king penguin. We were not able to definitively exclude the presence of an accessory ossification in any little, crested or yellow‐eyed penguin as we did not have access to the whole eye of the museum birds. Tiemeier (1950) noted that accessory ossifications in the posterior pole have been found in other birds but remarked that ‘they are not present in those birds that do not have the os opticus’, a statement that we can now disprove. We could not detect the scleral sesamoid bone (os sesamoideum esclerae) that Boho'rquez Mahecha and de Oliveira (1998) described adjacent to the scleral ring in most owls and the common potoo (*Nictibius griseus*).

We also identified a cartilaginous cup in each eye of one Snares crested penguin (AV 10449, Figure 8). This has also been thought to have a supportive function, possibly to prevent deformation during accommodation (Fischer & Schoenemann, 2019). As with the scleral ring, its presence suggests that penguins are able to accommodate as they transition from air to water and back.

This study was limited by the small number of scleral rings examined and particularly by the small number of skulls available for examination of accessory skeletal elements in the eye. Although this means we probably have not captured the full range of variation in such elements, even with our small numbers the range of variation was demonstrably large. We also relied on using the ring width to determine the correct orientation for the museum specimens, verifying this by using micro‐CT of the whole skull to ensure that this was appropriate, as they were not in situ at the time of examination. Our rings may also have been distorted due to differing methods of preparation, drying and long periods of storage and this does introduce some potential error.

Further studies to understand the way in which the scleral ring provides strength to the eye are required to better understand its function. It would be particularly useful to understand how the number and overlap of ossicles changes the stability and strength of the ring. Knowing what forces the internal struts are designed to overcome would also help us understand whether the ring also plays a part in resisting compression at depth.

## 5 CONCLUSIONS

This study demonstrated the variability of scleral rings and accessory skeletal elements in penguins. Although limited by only examining with micro‐CT three whole heads and recognising that care needs to be taken to minimise perspective distortion when interpreting macro photographic and micro‐CT images of 3D structures, it supports the premise that the correct orientation and laterality of isolated scleral rings can be determined as they are asymmetric and there is a dorsal ‘+’ element. Micro‐CT also demonstrated the complex internal structure of the scleral ring and, in all penguins we examined, it was not solid bone. Digital analysis of micro‐CT images may be helpful to determine the genus of a penguin using the scleral ring alone. This study also provides further evidence that the scleral ring is involved in globe protection and shows that it serves as an attachment site for the rectus muscles.

Finally, the extreme variability in accessory skeletal elements in even different members of the same species suggests that they are not essential to normal function, although there does appear to be a relationship between the os opticus, if present, and the muscles that move the nictitating membrane.

## AUTHOR CONTRIBUTIONS

**Peter W. Hadden:** Conceptualization; formal analysis; funding acquisition; investigation; methodology; project administration; validation; visualization; writing‐original draft; writing‐review and editing. **Dane A. Gerneke:** Conceptualization; data curation; formal analysis; investigation; methodology; project administration; resources; software; validation; visualization; writing‐review and editing. **Charles N. J. McGhee:** Conceptualization; formal analysis; funding acquisition; methodology; project administration; resources; supervision; validation; writing‐review and editing. **Jie Zhang:** Conceptualization; formal analysis; funding acquisition; investigation; methodology; project administration; resources; software; supervision; validation; visualization; writing‐review and editing.

### PEER REVIEW

The peer review history for this article is available at https://publons.com/publon/10.1002/jmor.21354.

## Supporting information


**Appendix** **1**. The scleral rings included in this study. The side to which the ossicle belonged and its orientation were determined according to the criteria described in the text. The orientation of the macro photographs reflects the orientation of the specimen as photographed, which is random, whereas the orientation of the micro‐CT images is in the same orientation as it is in the animal, as evidenced by surrounding bones in some images. ‘VZ’ in the ‘Number of ossicles’ column indicates that a verzahnung contact was present; in no specimen was there more than 1 verzahnung contact present. Scale bars = 5 mm.

**Appendix** **2**. This graph demonstrates how the width of the ossicle plate and the ratio of the width to the inner diameter of the ring appear to vary depending on the CTVox camera angle, which in turn is a function of how close the camera is to the sample and the size of the sample. The distortion increases sharply beyond 70°, especially of the ratio. These values are, in fact, invariant, and can be measured absolutely using programmes such as DataView (version V1.5.4.0, Bruker), the programme used for calibration in this study to ensure the optical macro images and micro‐CT viewpoints were as close as possible.

**Supplementary File 1**. The sclerotic ring of birds.

## Data Availability

The data that support the findings of this study are openly available in institutional figshare at http://doi.org/10.17608/k6.auckland.c.5114849, the University of Auckland Data Publishing and Discovery Service.
