# Supplementary material for: Skeletal elements of the penguin eye and their functional and phylogenetic implications (Aves: Sphenisciformes: Spheniscidae)
Source: J Morphol. 2021 May 2;282(6):874–86. doi: 10.1002/jmor.21354 (PMC8252517; doi:10.1002/jmor.21354)
Supplement: Supplementary file 1 — Appendix 1. The scleral rings included in this study. The side to which the ossicle belonged and its orientation were determined according to the criteria described in the text. The orientation of the macro photographs reflects the orientation of the specimen as photographed, which is random, whereas the orientation of the micro‐CT images is in the same orientation as it is in the animal, as evidenced by surrounding bones in some images. ‘VZ’ in the ‘Number of ossicles’ column indicates that a verzahnung contact was present; in no specimen was there more than 1 verzahnung contact present. Scale bars = 5 mm. [file JMOR-282-874-s002.docx]

| **Penguin type and collection number** | **Origin** | **Image** | **Right / Left eye (Collection label)** | **Number of**  **ossicles** | **Lemmrich**  **notation** |
| --- | --- | --- | --- | --- | --- |
| Little AV 959 | Westwood, Dunedin, Otago | 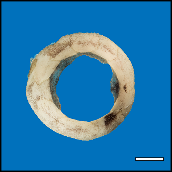 | R (A) | 11 | 1,6;3,9 |
|  |  | 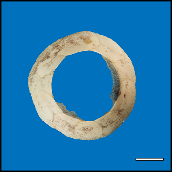 | L (B) | 10; VZ | 1,6;3,9 |
| Little AV 957 | Little Papanui, Dunedin, Otago | 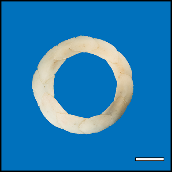 | R (A) | 11 | 1,7;4,9 |
|  |  | 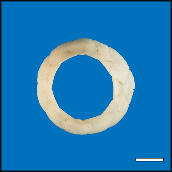 | L (B) | 11 | 1,7;4,9 |
| Little AV 958 | Moeraki, North Otago | 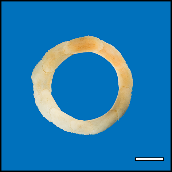 | R (B) | 10 | 1,6;4,8 |
|  |  | 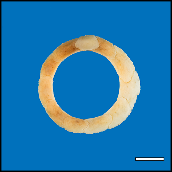 | L (A) | 11 | 1,7;4,9 |
| Little L1 | Auckland | 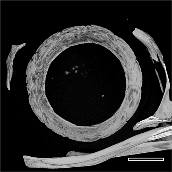 | R | 11 | 1,7;4,9 |
|  |  | 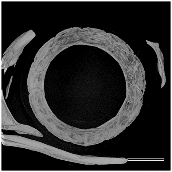 | L | 11 | 1,7;4,9 |
| Little AV 800 | Otago | 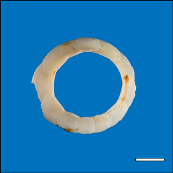 | R (A) | 12 | 1,7;4,9 |
|  |  | 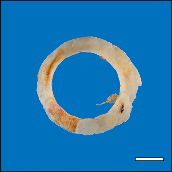 | L (B) | 12 | 1,7;4,10 |
| Fiordland  AV 966 | Dagg Sound, Fiordland | 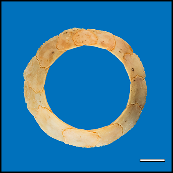 | R (B) | 13; VZ | 1,7;3,10 |
|  |  | 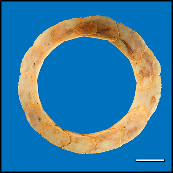 | L (A) | 14 | 1,9;5,11 |
| Fiordland  AV 7889 | Katiki, North Otago | 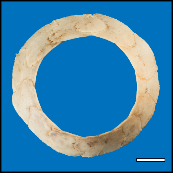 | R (A) | 12 | 1,6;3,10 |
|  |  | 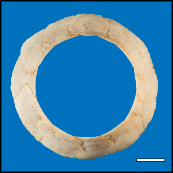 | L (B) | 13 | 1,7;4,11 |
| Fiordland  AV 1179 | Unknown | 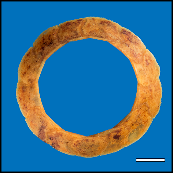 | R (B) | 13 | 1,7;4,10 |
|  |  | 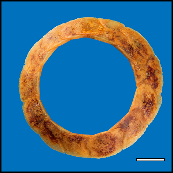 | L (A) | 14 | 1,7;4,11 |
| Fiordland  AV 964 | Unknown | 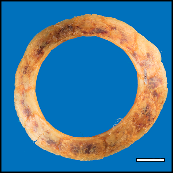 | R (B) | 12 | 1,8;4,10 |
|  |  | 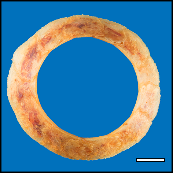 | L (A) | 12 | 1,7;4,11 |
| Fiordland  AV 1134 | Unknown | 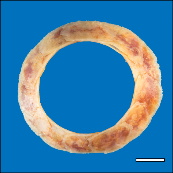 | R (A) | 12 | 1,6;3,8 |
|  |  | 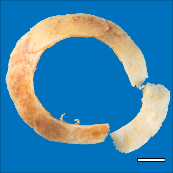 | L (B) | 12; VZ | 1,6,9;3,8,10 |
| Snares  AV 1909 | Oamaru, North Otago | 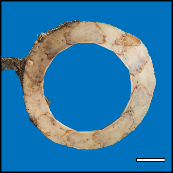 | L | 12 | 1,6;4,10 |
| Snares  AV 1178 | Otago Peninsula | 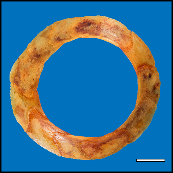 | R (B) | 12 | 1,8;4,10 |
|  |  | 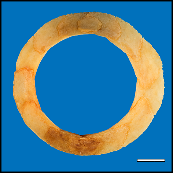 | L (A) | 14 | 1,8;4,11 |
| Snares  AV 10449 | Unknown | 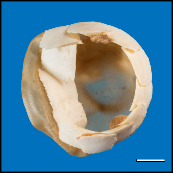 | R (B) | 12 | 1,7;4,10 |
|  |  | 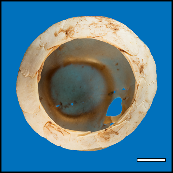 | L (A) | 13; VZ  1 hidden ossicle | 1,3,6,11;2,4,10,12 |
| Royal  AV 11055 | Unknown | 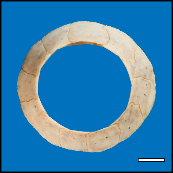 | R (A) | 12 | 1,6;3,9 |
|  |  | 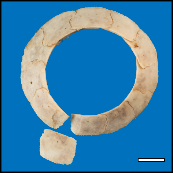 | L (B) | 12 | 1,6;3,9 |
| Yellow-eyed  AV 1181 | Unknown | 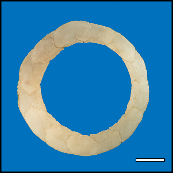 | L (C) | 13; VZ | 1,7;4,11 |
| Yellow-eyed  AV 7419 | Unknown | 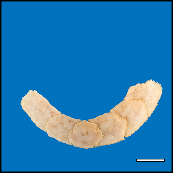 | Fragment only; unable to determine side |  |  |
| King K1 | South Georgia | 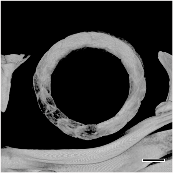 | R | 14 | 1,9;5,12 |
|  |  | 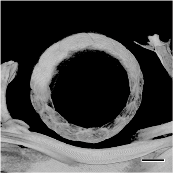 | L | 14 | 1,8;5,12 |
| King K2 | South Georgia | 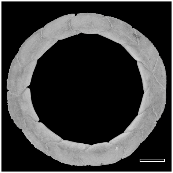 | L | 14; VZ | 1,8;5,12 |
| King K3 | South Georgia | 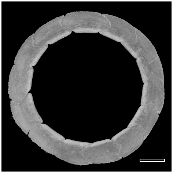 | L | 14 | 1,8;5,12 |
| Gentoo head G1 | Unknown | 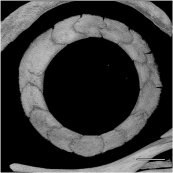 | R | 14; VZ | 1,8;5,11 |
|  |  | 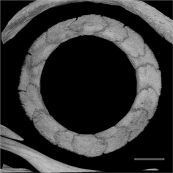 | L | 15 | 1,9;6,12 |
| Gentoo chick G2 | Unknown | 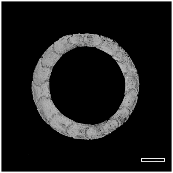 | R | 17; VZ | 1,11;11,14 |
