# Supplementary material for: Skeletal elements of the penguin eye and their functional and phylogenetic implications (Aves: Sphenisciformes: Spheniscidae)
Source: J Morphol. 2021 May 2;282(6):874–86. doi: 10.1002/jmor.21354 (PMC8252517; doi:10.1002/jmor.21354)
Supplement: Supplementary file 2 — Appendix 2. This graph demonstrates how the width of the ossicle plate and the ratio of the width to the inner diameter of the ring appear to vary depending on the CTVox camera angle, which in turn is a function of how close the camera is to the sample and the size of the sample. The distortion increases sharply beyond 70°, especially of the ratio. These values are, in fact, invariant, and can be measured absolutely using programmes such as DataView (version V1.5.4.0, Bruker), the programme used for calibration in this study to ensure the optical macro images and micro‐CT viewpoints were as close as possible. [file JMOR-282-874-s001.docx]

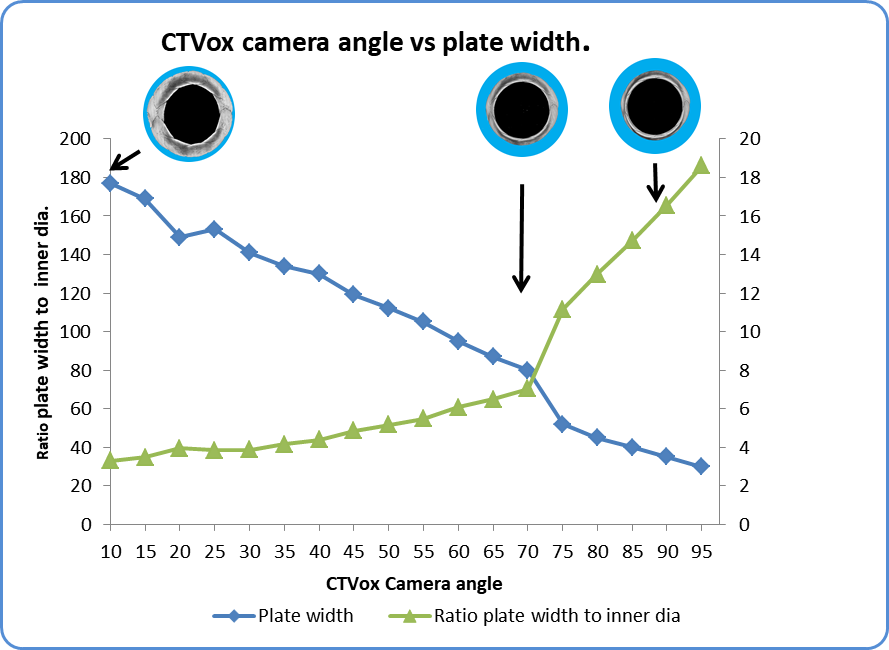


Appendix 2: This graph demonstrates how the width of the ossicle plate and the ratio of the width to the inner diameter of the ring appear to vary depending on the CTVox camera angle, which in turn is a function of how close the camera is to the sample and the size of the sample. The distortion increases sharply beyond 70˚, especially of the ratio. These values are, in fact, invariant, and can be measured absolutely using programs such as DataView (Version V1.5.4.0, Bruker), the program used for calibration in this study to ensure the optical macro images and micro CT viewpoints were as close as possible.
