# Supplementary material for: Skeletal elements of the penguin eye and their functional and phylogenetic implications (Aves: Sphenisciformes: Spheniscidae)
Source: J Morphol. 2021 May 2;282(6):874–86. doi: 10.1002/jmor.21354 (PMC8252517; doi:10.1002/jmor.21354)
Supplement: Supplementary file 3 — Supplementary File 1. The sclerotic ring of birds. [file JMOR-282-874-s003.docx]

**The sclerotic ring of birds**

By

Werner Lemmrich, Greiz

(translated by Claudia Bold and edited by Peter Hadden, 2020)

Includes 60 pictures within text.

Table of contents

1. Literature and the situation of the problem ……………………………………………………………………. 513
2. Systematic overview ……………………………………………………………………………………………………….. 520
3. The scales of the sclerotic ring ………………………………………………………………………………………... 551

Shape ……………………………………………………………………………………………………………….… 551

Quantity …………………………………………………………………………………………………………….. 552

1. The ring ………………………………………………………………………………………………………………………….. 566

Shape ……………………………………………………………………………………………………………….... 566

Structural design …………………..……………………………………………………………………………. 567

1. Sclerotic ring and genealogical tree ………………………………………………………………………………... 571
2. Histogenesis of the sclerotic ring ………………………………………………………………………………….... 574
3. Function of the sclerotic ring ……………………………………………………………………………………..…… 578
4. Summary ………………………………………………………………………………………………………………………... 582
5. **Literature and the situation of the problem.**

Although the bird’s eye has received much valuable attention in the last century in regard to its anatomy and physiology, the problem of the sclerotic ring, however, has been left standing somewhere in the middle or rather at the beginning of the previous century. It is a rather remarkable fact that this problem has not moved closer to a solution in any way in recent times. The workings of the last few years, that somehow try to address this, do not result in much more than the workings already carried out by GIEBEL (1857) and ALBERS (1802).

Based on the remarkable formation of the sclerotic ring in the eyes of birds, especially in birds of prey, it should not come as a surprise that its existence is not a recent discovery, but as per LEUCKART, Emperor Friedrich II (1194-1250) was already aware of it. Based on accounts from authors of the 17^th^ century, one can see that they knew of the existence of this ring and that VOLCHER COITER (1645) was the first writer to describe it in detail.

In the 18^th^ century, numerous authors have examined and described the sclerotic ring; some in a superficial manner, others more thoroughly. I agree with ALBERS, who states that PETIT, as one of the earliest authors, is the one that examined and described the sclerotic ring the most thoroughly. MERY (1701) and WARREN (1725) were in no way inferior to their contemporary PETIT due to the fact that WARREN not only determined the roof tile shaped formation but also the existence of especially covering plates; as well as to differentiate terrestrial and marine birds in respect to the number of plates. BOSE (1767), CALDANI (1784) and MALACARNE (1794) are authors whose works made only small, but nonetheless remarkable, contributions to the question regarding the sclerotic ring, whilst the works of DEMOURS (1741), CAMPER (1748), ZINN, MUSCHENBROEK (1762), OLBERS (1780), BLUMENBACH (1784) and E. HOME (1796) show that they knew of it, but also that they did not know to say anything new about it. It is known that there is 1 more author who makes his appearance towards the end of this century, and who describes the sclerotic ring in such a way as if he was the discoverer. What is incomprehensible, however, is that even after the observations of NUEL and HOSCH (1874) in the 2^nd^ half of the past century, the ring in birds of prey is apparently one single piece of bone as CANFIELD states.

There are not even close to as many works from the 19^th^ century that need to be mentioned compared to the previous century. Only 2 present themselves but they do not go into any more depth than the others but rather constitute more of a broadening of the topic. Those are the works of ALBERS (1802) and GIEBEL (1857). A progress in the works of GIEBEL, in which the handwritten remains of NITZSCH now appear as published, is very apparent. In it we find an impressive number of sclerotic rings described in pictures as well as words and one can get a rough idea of the structure of a ring. There are however two things regarding this work that I must mention straight away at this point. The first and most apparent is that one encounters mistakes made by the author as well as errata in various places. The second is that this work cannot make for a neat conclusion as the processing of the material was not done with the necessary aspects in mind. I will elaborate more in detail on these points at a later stage. Besides the description of 37 species with regards to the number of scales, measurements of the ring and the position of individual scales, ALBERS also dedicates one chapter to the meaning of the ring with the heading “Vom Nutzen des Augenringes”.

Because GIEBEL’s work is the only one that describes a tremendous number of rings next to each other, it became the basis of information for the newer works, textbooks as well as the other reference material. Only in the most recent times have authors begun to provide input again. Worthy of mention here are the works of TILLY EDINGER and A. DABELOW, that address the phylogeny and ontogeny of the sclerotic ring and finally bring its functional meaning up for discussion. It is also important to mention the contributions of authors of the statistics; here G. ISCHREYT issues not only detailed descriptions of form and layout of the scales but also some figures regarding the ring of a duck’s eye and PARREIDT provides details regarding the one of a penguin. A few other authors give only a few more examples.

Neither the specialised works nor the reference books give a complete and clear picture of the presently known facts regarding the sclerotic ring. Therefore, I would like to quickly outline everything that is known so far in the following pages.

The sclerotic ring is the ossification of the distal part of the sclera, the part that stretches between the cornea-sclera edge and the cartilaginous sclera. It does not consist of one piece of bone in the shape of a ring but is made up of individual plates that are positioned in the form of a ring. Those plates – often referred to as scales in the literature – lie on one another like roof tiles. MERY states the following about the sclerotic ring: *“that it consists of scales just like fish have and that they are arranged one on top of the other just like fish again”*. One cannot speak of a circular consistent coverage in the shape of roof tiles because there will always be a few scales that do not cover in that way. The kind of cover of those special scales is in such a way that the one scale covers both sides, whereas the other one is being covered by both its neighbouring scales. WARREN already recognized this fact correctly by saying: *“Their position is such that one bone covers the end of the other two, then there are three or four arranged like fish scales one over the other, on top of this there is again a bone under the end of other two and now there are again two or three arranged like fish scales”.*

GIEBEL not only mentions the number of covering and covered scales, as ALBERS does, but also shows the position of those scales in text and pictures. Regarding the number of the **ausgezeichneten** scales, which is what I would like to call both the covered as well as the covering scales, GIEBEL concludes – which I will repeat word for word: *“The number of completely covered scales varies between 1-3, and usually one finds as many completely covering scales as completely covered ones, in many exceptions one more completely covering scale than just one covered one”.* How GIEBEL comes to this conclusion that one can find “*in many exceptions one more completely covering scale than just one covered one”* is hard to explain. In a ring with coverage in the form of roof tiles there has to be the same number of covered scales as well as covering scales; the one ausgezeichnete scale is necessarily the result of the other one. Even ALBERS mentions a number of examples which apparently show an uneven number of ausgezeichnete scales. It is possible that an ausgezeichnete scale is interlocked with its neighbouring scale. That this however happens in many exceptions and only with covering scales is, in my opinion, impossible. From GIEBELS interpretations one must assume that he has not yet noticed the interlocking scales. I would not make a point of this statement if this inaccuracy had not found its way into one of the newest works.

Although it is not difficult at all to recognize the individual elements of the ring in most birds, NUEL and HOSCH (1874) have apparently observed that the ring in birds of prey is one piece of bone only. Furthermore, THOMAS ALLIS states a case in which he observed one single ring. In “Proceedings of the zoological society of London, Part V. 1837” one can read a letter in which ALLIS observes that the sclerotic ring of the big *Podargus* did not consist of individual plates but was rather a circular piece of bone – “being simply a bony ring”. I beg to differ that such observations are correct, however one cannot simply draw a conclusion on this.

The few statements made about the number of scales that we can find in the literature were almost exclusively made by GIEBEL; he observes a maximum of 17 scales and a minimum of 10 and states as the usual amount 13, 14 and 15 scales.

FRANZ speaks for the first time of a “mutual wedging of the scales” and refers thereby to the occurrence that 2 scales mutually cover each other at the edge in both kinds of coverage, i.e. scale A partially covers scale B and that it is being partially covered by it and vice versa. I have called this phenomenon interlocking as KOPSCH has (1892 page 44). We know very little about the function and meaning of the sclerotic ring; it appears that it has a supporting function.

GIEBEL considers the conditions for the development of the sclerotic ring to be important for the classification, which prompted him to publish NITZSCHE’s data. GADOW on the contrary reaches the opinion that due to that same data “the ring of bone can hardly be of systematic importance”. I personally find it very bold to try and answer such questions successfully with merely GIEBEL’s findings, which do not give a lot of clarity on the conditions of the sclerotic ring and are not free from errors in the first place.

When observing some randomly selected rings, one is tempted to deny any regularities in its structural design. It is this diversity that conceals the problem of the sclerotic ring. With that in mind we must make the following demand: the examination of numerous and diverse material. Only then will it be possible to get closer to the nature of the sclerotic ring. This task requires a large quantity of factual material, the more so as the rings will have to be examined in pairs if at all possible and because it is absolutely necessary to have a large number of specimens of certain species available. Therefore, my results can only be seen as a contribution and they will not be enough to find a solution to the problem of the sclerotic ring all at once. My main task is to offer material that is as far as possible free of errors and that offers a stable foundation for all questions that will arise from the existence of this ring.

Primarily, I will be talking about the number of scales. It is known that the sclerotic rings of the bird species consist of 10-17 scales. How the number of scales are arranged over the individual groups – order, family, species – requires thorough work as the scarce results so far do not offer much clarification. One has an approximate idea that they are not a random phenomenon on each individual and that any number must be typical for a specific group, which GIEBEL already noticed, even though this was based on “notably meagre observations”, as he himself said. This observation per se is not important to me; what is valuable, however, is to determine in particular the type and degree of the deviation. Only then will there be results from which one can make statements with fair certainty.

I will call those scales that interrupt the cycle of coverage ausgezeichnete scales. I am speaking of a **Plus-Scale** (+ -scale) – it covers on both sides – and of a **Minus-Scale** (⃝ -scale) – which is covered on both sides. ISCHREYT characterises the scales based on the appearance of its cross section and refers to a Plus-scale as “Stockgrifform” (stick grip shape) and to a Minus-scale as “Untertassenform” (saucer shape); all other scales are referred to by him as “rhombisch” (rhombic). Since a Plus-scale in this rooftile kind of coverage necessarily requires a minus-scale, it is justified to speak of a pair of scales. After the amount of ausgezeichnete scales in the rings, I then differentiate between 2 types: **B-type** with a pair of ausgezeichnete scales and **A-type** with two (or more) pairs of ausgezeichnete scales (text figure 1 a,b).

Text figure 1 a, b.

The ausgezeichnete scales (schematical).

a A-type.

b B-type.

*d* dorsal, *v* ventral.

Left is temporal, right is nasal.

The abbreviation A and B are the initial letters of the genus where I found these 2 types first and particularly often: *anas* and *buteo*. The A-type had already been correctly identified in ducks by ISCHREYT; he also describes the varying position of the ausgezeichneten scales between the different species due to the variation of the number of scales. With regards to the ausgezeichneten scales, the following main question is: to what extent is there a regularity in their formation? How does the positioning in the ring change from group to group? To get the necessary overview of all the existing rings, each scale will be numbered. This thought stemmed from GIEBEL; he states, “We count the scales from the first one at the bottom of the outside around to the inside until the starting point and thus identify the position and number of the only covering and the fully covered scales”.

Soon I noticed that each ring is the mirror image of its corresponding one, whereby the plane of symmetry runs through beak and crown. Furthermore, I was able to observe that the plus-scales are located in the dorsal-ventral line, bar minor deviations. So is the 1^st^ scale always a plus-scale. So much to be mentioned for now.

Each ring still needs to be looked at in terms of its other characteristics; what kind of interlocking there is and which scales are part of it. Perhaps one can discover also a regularity in this. The form of the scales, the degree of the coverage, the form of the ring; those are all aspects that need to be looked at. Any changes from species to species, whether it be in the number of scales, the formation of the scales or the type of scales, have to be closely examined to determine if it is typical or unique. I believe knowledge of the embryonic development of the rings is desirable for the clarification of a number of questions. It is to determine:

1. Number of scales in the right and left ring; within the individual groups.
2. Formation of the scales or structural design of the ring (the ausgezeichneten scales as characteristics of the rings).
3. Amount; type.
4. Formation
5. Other characteristics.
6. Of the scales.

aa) Interlocking

bb) Form.

cc) Degree of the coverage.

dd) “Markräume” (CANFIELD) (Hohlräume). *(cavities)*

1. Of the ring.

aa) Form.

1. Ontogenesis of the ring.

In identifying these as my scope of work it ought not express any limitations. On the contrary, the clarification of this formulated scope of work is supposed to clear the path to finding solutions regarding the questions regarding the meaning of the ring, which is being driven onward based on the findings.

1. **Systematic Overview**

Before I present my findings, I first need to make some remarks regarding the composition of the material. Using the findings of the existing literature in the form that we know it, is often not possible; provided we attach importance to the other results, one must mostly rework them. To be able to compare GIEBEL’s results to my own without any difficulties, I adopted his method to draw the scales which granted great clarity. Soon however, I noticed discrepancies between both findings, which were mostly based on mistakes in GIEBEL’s work. These discrepancies mostly refer to the fact that GIEBEL, contrary to his statement to count from the first scale at the bottom of the outside around to the inside, arguably counted almost exclusively in the wrong direction. This is the only comprehensible way why GIEBEL, for example, counted scales number 1 and 10 as ausgezeichnete scales for a sparrow hawk, whilst my own observation says it should be scales number 1 and 7. I do not find it necessary to discuss all the mistakes found in GIEBEL’s work. I have naturally only used those rings that, after my revision, I can wholeheartedly support for their correctness. It was astonishing to see that the number of rings that needed to be corrected was so high. Through a lot of effort, I managed to remedy these shortcomings.

Another flaw in GIEBEL’s work is the fact that he leaves us in the dark regarding the way in which he reached his conclusions. Are the written results always only based on an examined individual ring or were they there as pairs in all the species that he named? I find the latter very unlikely as I noticed that, in a great number of ring pairs, the number of scales in both rings is not the same; GIEBEL however stated hardly anything regarding that. In my systematic overview I will adhere to the system that we can find in BREHM, IV. Aufl. (Leipzig, Bibliogr. Institut). I will try to demonstrate the relationship between the individual rings in a short and clear manner, which is why I often use many abbreviations. The following example as a reference:

*Buteo buteo*, Mäusebussard *(common buzzard).*

B-type, right and left 15 scales, +-scales: 1; ⃝-scales: 7.

This would mean: the right and the left eye of a pair have been examined; both have B-type and 15 scales and on both eyes is the first scale a plus-scale and the seventh scale is a minus-scale. All possible amendments are now easy to understand. Following that is a short characteristic of the scales and ring in keywords and at times there may be short special remarks regarding the examined rings. According to the system I have inserted the observations from the other authors, orientated in accordance with my own observations and with the following note attached: based on GIEBEL (or similar). The observations made by GIEBEL are rendered as they should have been, however, I have made a note if I had to modify them. I tried to verify as many facts as possible through my own drawings and do not forgo any drawings if they were already available in GIEBEL’s work; I do not need to explain the reason for this again. I will not show a drawing of those rings that show in every respect the same as the other one, but only of those rings that show anything significantly new. If we are only interested in parts of a ring, such as interlockings, then I will only show section figures.

***Ratites.***

***Struthionidae,*** Strauβe***.*** *(Ostriches)*

*Struthio camelus,* gewöhnlicher Strauβ (*common ostrich)* [text fig. 2] ¹).

A-type, right and left 15 scales, +-scales: 1, 8; ⃝-scales: 5, 11.

Scales: more wide than tall, thin and flexible, well covered.

Ring: flat, slightly concave. The cartilaginous cup reaches far underneath the ring.

The ring of the ostriches is very similar to the ring of the passerine birds. We read in ALBERS work that RANBY also describes the ring of an ostrich as consisting of 15 scales.

***Casuariidae,*** Kasuare. *(Cassowary)*

According to GIEBEL: *Dromaeus novae-hollandiae,* Emu *(emu).*

A-type, left 15 scales, +-scales: 1, 6, 9; ⃝-scales: 5, 7, 12.

According to the text and drawing, only the 5^th^ scale is marked as a minus-scale. GIEBEL explicitly says: “and only the 5^th^ very narrow fully covered”. I believe the 7^th^ and 12^th^ scales are also minus-scales.

____________________________

1. The drawings of the rings represent projections. The interlockings are symbolically somewhat illustrated.

***Colymbiformes.***

***Colymbidae,*** Seetaucher***.*** *(Loon)*

According to GIEBEL: *Colymbus arcticus,* Polartaucher *(arctic loon).*

A-type, left 14 scales, +-scales: 1, 8, 11; ⃝-scales: 5, 10, 12.

Only the 1^st^ and 8^th^ are marked as +-scales. The drawing clearly shows that the 11^th^ is also a +-scale.

According to GIEBEL: *Colymbus septentrionalis,* Rotkehltaucher *(red-throated loon).*

Same as *C. arcticus* according to my data.

I cannot make any general remarks regarding diving birds. The existence of 3 pairs of ausgezeichente scales in both these representative samples seems to be a regular phenomenon. I do not doubt the existence of such rings; however, in all my examinations, I have only ever found 2 examples – 2 rings (hoopoe and goose) – which were in possession of 3 pairs of ausgezeichnete scales, whereby this only ever occurred in the left ring of a pair of rings. Noteworthy is that GIEBEL knows of 5 examples.

***Sphenisciformes.***

***Spheniscidae,*** Pinguine***.*** *(Penguins)*

*Spheniscus demersus,* Brillenpinguin *(jackass penguin).*

A-type, right and left 15 scales

right: +-scales: 1, 8; ⃝-scales: 5, 12

(text fig. 3 a, b).

left: +-scales: 1, 10; ⃝-scales: 5, 14

A-type, right 14 scales, +-scales: 1, 8; ⃝-scales: 5, 11 (text fig. 4)

Scales: more wide than tall, up to square shaped, thick, tightly interlocked with each other, well covered, small medullary spaces.

Ring: steep, slightly convex.

What is striking is that in text fig. 3a and b as well as in text fig. 4 one can find particularly small scales. In text fig. 3a scale 8 covers scales 7 and 9, while 9 simultaneously covers 7 and 10. Furthermore, the outer edge of the scale does not by any means reach the outer edge of the ring. Text fig. 4 shows a similar case. The only difference is that scale 1 covers scale 2 and 14, while scale 2 also covers 3 and 14. Text fig. 3b shows that scales 6 and 8 in the ring are very narrow; 8 does not even reach the inner edge of the ring. It seems to be due to both of these narrow scales that the formation of the ausgezeichneten scales in this ring is so unexpected.

*Aptenodytes patachonica,* Königspinguin *(king penguin)* (text fig. 5).

A-type, right 15 scales, +-scales: 1, 8; ⃝-scales: 4, 12.

Interlocked: 1 X 15.

Scales: partly taller than wider, well covered, tightly interlocked with each other.

Ring: ascending to steep, slightly convex.

According to T. EDINGER:

A-type, left 15 scales, +-scales: 1, 8; ⃝-scales: 4, 12.

According to those details, compared with my data, only the aforementioned results are possible.

According to PARREIDT:

*Catarrhactes chrysocome,* Felsenpinguin *(rockhopper penguin).*

A-type, right 12 scales, +-scales: 1, 8; ⃝-scales: 5, 10.

Interlocked: 7 X 8.

PARREIDT said, and I quote: “… 12 scales, of which 2 are fully covered, while 1 is fully covering.” Based on the drawing, one can see that the 4^th^ ausgezeichnete scale is interlocked with a neighbouring scale and therefore yields the aforementioned formation.

It is not easy to establish a predominating form based on this order, as all rings somehow vary. What is consistent, is the A-type, the shape of scales and rings and the strong interlocking of the scales with one another; predominant is the number 15 and the formation of the scales in such a way that 1 and 8 are plus-scales and 5 and probably 12 minus-scales.

Text fig. 2-6b. *Ratites. Sphenisciformes. Procellariiformes.*

Text fig. 2. Common ostrich, right, ¾;

Text fig. 3a, b;

Text fig. 4. Jackass penguin, right and left, right, ¾;

Text fig. 5. King penguin, right, ¾;

Text fig. 6a, b. albatross, right and left, ¾.

***Procellariiformes.***

***Procellariidae,*** Sturmvögel***.*** *(petrels and shearwaters)*

*Diomedia regia Bulleri,* Albatros *(albatross)* (text fig. 6a, b).

A-type, right 13 and left 14 scales;

Right: +-scales: 1, 8; ⃝-scales: 4, 9;

Left: +-scales: 1, 9; ⃝-scales: 4, 10.

Interlocked: left 11 X 12, 6 X 7 X 8.

Scales: wider than tall, well covered, many medullary spaces.

Ring: slightly raised

According to T. EDINGER: 14 scales.

***Ciconiiformes.***

***Sulidae,*** Tölpel***.*** *(gannets)*

*Sula bassana,* gewöhnlicher Tölpel *(common gannet).*

A-type, right 12 scales, +-scales: 1, 7; ⃝-scales: 4, 10.

Scales: taller than wide, sparsely covering

Ring: steep, weak, concave.

***Ardeidae,*** Reiher***.*** *(herons)*

*Ardea cinerea,* grauer Fischreiher *(grey heron)* (text fig. 7).

A-type, left 14 scales, +-scales: 1, 8; ⃝-scales: 5, 10.

Interlocked: 4 X 5 (slightly).

Scales: wider than tall, slightly curved (with wing formation), tightly interlocked.

Ring: flat.

According to GIEBEL:

A-type, left 15 scales, +-scales: 1, 8; ⃝-scales: 5, 10.

According to text: ⃝-scale: 5, 10; according to drawing: 5, 11.

I adopt the statements made in the text; ALBERS examines 15 scales as well.

*Botaurus stellaris,* Rohrdommel *(bittern).*

A-type, right and left 14 scales, +-scales: 1, 8; ⃝-scales: 5, 10.

Scales: wider than tall, laterally rounded, contiguous on the outer edge, well covered.

Ring: slightly erect, concave.

According to GIEBEL:

A-type, left 14 scales, +-scales: 1, 8; ⃝-scales: 5, 10.

According to text: +-scales: 1, 8; ⃝-scales: 4, 13; this is however the conclusion based on a counting in the incorrect way; the drawing also clearly shows the formation as I have found it.

According to GIEBEL:

*Nycticorax nycticorax,* Nachtreiher *(night heron).*

A-type, left 14 scales, +-scales: 1, 8; ⃝-scales: 5, 10.

***Ciconiidae,*** echte Störche***.*** *(storks)*

According to ALBERS:

*Ciconia Ciconia*, Hausstorch *(white stork).*

A-type, 14 scales.

***Ibidae,*** Ibisvögel***.*** *(ibises)*

According to GIEBEL:

*Platalea leucerodea*, Löffler *(common spoonbill).*

A-type, left 15 scales, +-scales: 1, 9; ⃝-scales: 5, 11.

The text only mentions the formation of the ausgezeichneten scales as described in the aforementioned. It is not possible to determine how many scales in any case based on the text and pictures; however, 15 scales are highly likely.

This order is quite consistent in regard to the number and formation of scales. *Sula bassana* stands in particular contrast to the other ones, provided that this is not an exception. I, in fact, believe it is more probable that a grey heron has 14 scales instead of 15 as GIEBEL and ALBERS have examined, because the fact that matters to conclude this, is that all close relatives have 14 scales with the same formation.

***Anseriformes.***

***Palamedeidae,*** Wehrvögel***.*** *(screamers)*

*Chauna chavaria* (text fig. 8a,b).

A-type, right 15 scales and left 12 scales;

Right: +-scales: 1, 8; ⃝-scales: 6, 11;

Left: +-scales: 1, 8; ⃝-scales: 5, 9.

Interlocked: right 1 X 2, 3 X 4, 6 X 7, 13 X 14;

Left: 5 X 6, 7 X 8.

Scales: wider than tall, slightly round, sizes vary a lot, especially wide in the left ring.

Ring: slightly erect.

***Anseridae,*** Gänse***.*** *(geese)*

According to GIEBEL:

*Mergus merganser*, Gänsesäger *(goosander).*

A-type, left 15 scales, +-scales: 1, 7, 9; ⃝-scales: 5, 8, 11.

According to text: 1, 7, 14 and 3, 12, 15 and as the right ring, as it was counted from top to bottom in the picture. In my opinion this is however a left eye and the formation is as per the aforementioned. Particularly interesting is the fact that 3 pairs of ausgezeichnete scales could be witnessed.

According to ISCHREYT:

*Oedemia fusca*, Samtente *(velvet scoter).*

A-type, left 15 scales, +-scales: 1, 8; ⃝-scales: 6, 12.

According to GIEBEL:

*Netta rufina*, Kolbenente *(red-crested pochard)*: 15 scales

According to GIEBEL:

*Fuligula fuligula*, Reiherente *(tufted duck)*.

A-type, right 15 scales, +-scales: 1, 9; ⃝-scales: 5, 11.

According to text: 1, 8, 6, 12; that would be a left eye, at which point nasal would be wider than temporal. The corrected results are equal to the results I found in ducks that I examined myself.

According to GIEBEL:

*Clangula clangula*, Schellente *(goldeneye)*.

A-type, left 15 scales, +-scales: 1, 9; ⃝-scales: 5, 11.

According to text: 1, 9, 5, 14; again it was counted from top to bottom. The corrected counting based on the drawing gives the aforementioned results.

According to ISCHREYT:

A-type, left 15 scales; +-scales: 1, 9; ⃝-scales: 4, 11.

*Anas boscas*, Stockente *(mallard duck)*.

A-type, right and left 15 scales; right and left +-scales: 1, 9;

Right: ⃝-scales: 5, 12.

Left: ⃝-scales: 5, 11.

Scales: wider than tall, thin, without medullary spaces, rounded, well covered.

Ring: flat.

According to ISCHREYT:

A-type, left 16 scales, +-scales: 1, 9; ⃝-scales: 5, 13.

*Mareca penelope*, Pfeifente *(wigeon)*.

1. A-type, right and left 15 scales, +-scales: 1, 9; ⃝-scales: 5, 11 (text fig. 9).

2. The same as under 1.

Interlocked: right 7 X 8 X 9 (text fig. 10).

3. A-type, right and left 15 scales, +-scales: 1, 9;

Right: ⃝-scales: 5, 11.

Left: ⃝-scales: 3, 11 (text fig. 11).

The left ring of the 3^rd^ pair is particularly interesting. We can witness an anomaly in the structure and the formation of the scales. Scale 4 and 5 do not have the normal size regarding their height. They are situated on the inner edge of the ring and therefore leave a big gap in the ring between scales 3 and 6. Scale 4 covers scale 3 and is being covered by scale 6; furthermore scale 5 covers scales 6 and 4.

According to ISCHREYT:

A-type, left 15 scales, +-scales: 1, 9; ⃝-scales: 5, 13.

According to ISCHREYT:

*Querquedula querquedula*, Knäkente *(garganey)*.

A-type, left 15 scales, +-scales: 1, 9; ⃝-scales: 5, 13.

*Dafila acuta*, Spieβente *(pintail)*.

1. A-type, right and left 15 scales, +-scales: 1, 9; ⃝-scales: 5, 11.

2., 3., 4. The same as under 1.

5. A-type, right 16, left 15 scales;

Right: +-scales: 1, 10; ⃝-scales: 5, 12.

Left: +-scales: 1, 9; ⃝-scales: 5, 11.

Interlocked: left 4 X 5 (marginally).

*Blutschnabelente* *(rosy-billed pochard)* (text fig. 12).

A-type, right 16, left 15 scales, right and left: +-scales: 1, 9;

Right: ⃝-scales: 5, 13;

Left: ⃝-scales: 5, 11.

The 9^th^ scale in the right ring is particularly small and seems to have given the impetus to anomaly.

With regards to the scales and rings of the aforementioned ducks, I can only say the same as under *Anas boscas*.

Text fig. 7-14b. *Ciconiiformes. Anseriformes.*

Text fig. 7. Grey heron, left, ⅟₁; text fig. 8a, b. *Chauna chavaria*, right and left, ⅟₁; text fig. 9. wigeon, right, ⁵∕₃; text fig. 10. wigeon, interlocking right, ⁵∕₃; text fig. 11. wigeon, left anomaly, ⁵∕₃;

text fig. 12. rosy-billed pochard, right part, ⅟₁; text fig. 13a, b. domestsic goose, right and left, ⅟₁; text fig. 14a. domestsic goose, right, ⁴∕₃; text fig. 14b. domestsic goose, right, scales 5, 6, 7, ⁵∕₃.

According to GIEBEL and ALBERS:

*Spatula Clypeata*, Löffelente *(shoveler)*: 14 scales.

According to GIEBEL:

*Alopochen ägyptheacus*, Nilgans *(egyptian goose)*: 15 scales.

*Anser anser domesticus*, Hausgans *(domestic goose)*.

1. A-type, right 16 scales and left 18 scales;

Right: +-scales: 1, 9; ⃝-scales: 5, 11.

Left: +-scales: 1, 8, 10; ⃝-scales: 6, 9, 13 (text fig. 13a, b).

Interlocked: right 8 X 9 and left 7 X 8.

2. A-type, right 18 scales, +-scales: 17, 9; ⃝-scales: 4, 12.

Interlocked: 5 X 6 X 7 and 9 X 10 (text fig. 14a).

Scales: thin, wider than tall, at times taller than wide, slightly rounded, well covered.

Ring: flat.

The left ring under 1. shows 3 pairs of ausgezeichnete scales. The ring under 2. is structured in a noticeably abnormal way. To emphasize the interlocking, I drew 3 scales very clearly (text fig. 14b).

ALBERS also states A-type and 16 scales; GIEBEL the same amount.

According to GIEBEL:

*Branta leucopsis*, Nonnengans *(barnacle goose)*.

A-type, right 13 scales, +-scales: 1, 8; ⃝-scales: 4, 12.

According to GIEBEL:

*Cairina moschata*, Moschusente *(muscovy duck)*.

A-type, right 15 scales, +-scales: 1, 9; ⃝-scales: 5, 10.

According to GIEBEL:

*Cygnus cygnus*, Singschwan *(whooper swan)*.

A-type, right 15 scales, +-scales: 1, 5, 8; ⃝-scales: 4, 6, 12.

ALBERS also counts 15 scales.

According to GIEBEL:

*Chenopsis atrata*, Schwarzschwan *(black swan)*.

A-type, left 16 scales, +-scales: 1, 10, 13; ⃝-scales: 7, 12, 14.

*Chauna chavaria* varies widely; it is possible that this is only to be seen as a unique occurrence. A strong uniformity can be seen on the other representatives. Number 15 is the most common and the formation 1, 9, 5, 11 can be examined in almost all of the rings, even Mergus merganser, with its 3 pairs of ausgezeichnete scales, includes this formation.

***Falconiformes.***

***Vulturidae,*** Geier***.*** *(vultures)*

*Lophogyps occipitalis* (text fig. 15a, b).

A-type, right 17 scales, left 15 scales;

Right: +-scales: 1, 10; ⃝-scales: 7, 13.

Left: +-scales: 1, 9; ⃝-scales: 5, 11 (?).

Interlocked: right 1 X 14, 10 X 11, 12 X 13;

Left: 1 X 15, 5 X 6, 9 X 10.

Scales: taller than wide, size not consistent, hardly covering.

Ring: rising.

There are 2 small scales in the right ring. Scales 11, 12 of the right ring and 10, 11 of the left ring touch each other or the overlapping is so small that it cannot be recognized as such.

According to GIEBEL:

*Gyps fulvus*, Gänsegeier *(griffon)*.

B-type, right and left 15 scales, +-scales: 1; ⃝-scales: 6.

According to text: 1 and 11; but this was counted the incorrect way.

***Falconidae,*** Falkenvögel***.*** *(falcons)*

According to GIEBEL:

*Gypaëtus barbatus*, Lämmergeier *(bearded vulture)*.

A-type, right 14 scales, +-scales: 1, 7, 11; ⃝-scales: 5, 8, 12.

According to text: 1, 5, 9 and 8, 11. The drawing shows that this was counted in the wrong direction. There is a minus scale missing, which can however be clearly seen in the drawing.

According to GIEBEL:

*Haliaëtus albicilla*, Seeadler *(sea eagle)*.

A-type, left 14 scales, +-scales: 1, 7; ⃝-scales: 6, 8.

According to text: 1, 7 and 2, 14; according to the picture this was counted starting from dorsal.

*Circaëtus gallicus*, Schlangenbussard *(Congo serpent eagle)*.

A-type, right 16 scales, +-scales: 1, 9; ⃝-scales: 7, 12.

Interlocked: 7 X 8 X 9.

Scales: taller than wide, furnished with spikes (wings), many medullary spaces, little to well covered.

Ring: ascending to steep.

GIEBEL mentions 15 scales.

*Spizaëtus ornatus* (text fig. 16).

A-type, right and left 13 scales, +-scales: 1, 8; ⃝-scales: 6, 10.

Interlocked: right 1 X 2 X 3, 5 X 6, 9 X 10.

Left: 1 X 2, 5 X 6, 9 X 10.

Scales: taller than wide up to square shaped, little to well covered, on the outside partly bumping each other, many medullary spaces.

Ring: steep.

According to GIEBEL:

*Aquila chrysaëtus*, Steinadler *(golden eagle)*.

A-type, right 15 scales, +-scales: 1, 8; ⃝-scales: 5, 10.

According to the text GIEBEL stipulates 1, 9 and 5, 10 but the drawing very clearly shows 1, 8 and 5, 10; according to T. EDINGER: 15 scales.

According to GIEBEL:

*Aquila maculata*, großer Schreiadler *(spotted eagle)*.

B-type, right 15 scales, +-scales: 1; ⃝-scales: 7.

In the text, GIEBEL stipulates 1 and 10, as well as for sparrow hawk. Counted the wrong way. The corrected counting concludes as per the above mentioned, also for the sparrow hawk.

According to GIEBEL:

*Archibuteo lagopus*, Rauhfußbussard *(rough-legged buzzard)*.

B-type, right 15 scales, +-scales: 1; ⃝-scales: 6.

ALBERS also states b-type and 15 scales.

*Buteo buteo*, Mäusebussard *(common buzzard) (text fig. 17a)*.

1. B-type, right and left 15 scales, +-scales: 1; ⃝-scales: 7.

2.-6. The same as under 1.

7. B-type, right 15, left 14 scales, +-scales: 1; ⃝-scales: 7.

8. B-type, right and left 15 scales, +-scales: 1; right: ⃝-scales: 6; left: ⃝-scales: 7.

9. B-type, right and left 15 scales, +-scales: 1; right: ⃝-scales: 7; left: ⃝-scales: 6.

Interlocked: 1.-3., 7.: right and left 6 X 7 (text fig. 17b, c); 4., 5., 8.: left 6 X 7; 6.: right 6 X 7;

4.: right 13 X 14 (text fig. 17d); 9.: left 1 X 15 (text fig. 17e).

Scales: taller than wide, many medullary spaces, hardly covered.

Ring: steep, slightly convex.

According to GIEBEL: B-type, right 15 scales, +-scales: 1; ⃝-scales: 6.

*Pernis apivorus*, Wespenbussard *(honey buzzard) (text fig. 18)*.

A-type, right and left 15 scales, +-scales: 1, 9; ⃝-scales: 5, 10.

Scales: taller than wide, hardly covering.

Ring: ascending, flatter than the one from *Buteo b.*

According to GIEBEL: A-type, right 15 scales, left 16 scales; left: +-scales: 1, 9; ⃝-scales: 8, 10.

According to ALBERS: B-type, 15 scales.

*Harphyhaliaëtus coronatus.*

A-type, right 15 scales, +-scales: 1, 8; ⃝-scales: 6, 9.

Scales: taller than wide, many medullary spaces, hardly covering.

Ring: steep, especially steep on the inner edge.

According to GIEBEL:

*Milvus milvus*, Königsweihe *(red kite).*

B-type, right 15 scales, +-scales: 1; ⃝-scales: 6.

According to text: 1 and 11; the same mistake as *Aquila maculata*.

*Accipiter nisus*, Sperber *(sparrow hawk) (text fig. 19a)*.

1. B-type, right and left 15 scales, +-scales: 1; ⃝-scales: 6.

2. and 3. the same as under 1.

4. B-type, right 15 and left 16 scales, +-scales: 1; ⃝-scales: 7.

5. B-type, right and left 15 scales, +-scales: 1; right: ⃝-scales: 6; left: ⃝-scales: 7.

6. B-type, right 16 scales and left 14 scales, +-scales: 1; right: ⃝-scales: 7; left: ⃝-scales: 6.

Interlocked: 2.: left 6 X 7 (text fig. 19c); 4.: left 7 X 8 (text fig. 19d); 5.: right 6 X 7 (text fig. 19b).

Scales: taller than wide up to square shaped, many medullary spaces, hardly covering.

Ring: ascending.

According to GIEBEL:

B-type, left 15 scales, +-scales: 1; ⃝-scales: 7.

Refer to remarks made under *Aquila maculata*.

ALBERS also states B-type and 15 scales.

*Astur palumbarius*, Hühnerhabicht *(goshawk).*

1. B-type, right and left 15 scales, +-scales: 1; ⃝-scales: 8.

2. B-type, right 15 scales and left 16 scales, +-scales: 1; ⃝-scales: 7.

3. B-type, left 16 scales, +-scales: 1; ⃝-scales: 8.

Interlocked: 1.: right and left 5 X 6 X 7 X 8; 3.: 6 X 7 X 8.

Scales: taller than wide, little to well covered.

Ring: ascending.

ALBERS states b-type and 16 scales; whereas GIEBEL notes 14 scales.

Text fig. 15a-19d. *Falconiformes.*

Text fig. 15a,b. *Lophogyps occipitalis*, right and left, ⅟₁; text fig. 16 *Spizaëtus ornatus*, right, ⅟₁; text fig. 17a-e. Common bussard, ⅟₁; a left, b right scales 6-8, c left scales 6-8, d right scales 13-15, e left scales 15, 1, 2; text fig. 18. Honey buzzard, right, ⅟₁; text fig. 19a-d. sparrow hawk, ⅟₁; a right, b right scales 6, 7, c left scales 6, 7, d left scales 7, 8.

*Circus cyaneus*, Kornweihe *(hen harrier).*

B-type, right 15 scales, left 14 scales, +-scales: 1; ⃝-scales: 8.

Interlocked: right 7 X 8 X 9; left 6 X 7 X 8.

The scales 6 and 7 of the right ring touch each other without coverage.

Scales: wider than tall up to square shaped, hardly covering, with many and big medullary spaces,

Ring: ascending.

According to GIEBEL:

*Circus pygargus*, Wiesenweihe *(montagu’s harrier):* 15 scales.

According to GIEBEL:

*Circus aeruginosus*, Rohrweihe *(marsh harrier).*

B-type, right 15 scales, +-scales: 1; ⃝-scales: 8.

According to GIEBEL:

*Falco peregrinus*, Wanderfalke *(peregrine falcon).*

B-type, right 14 scales, +-scales: 1; ⃝-scales: 7.

According to GIEBEL:

*Falco subbuteo*, Baumfalke *(Eurasian hobby):* 15 scales.

And also *Falco aesalon*, Merlin *(merlin).*

According to GIEBEL:

*Cerchneis tinnunculus*, Turmfalke *(kestrel).*

B-type, left 14 scales, +-scales: 1; ⃝-scales: 7.

ALBERS and T. EDINGER state the same as above.

In birds of prey we can find B-type as well as A-type; however, B-type is more common than A-type. Noteworthy is the fact that from the 14 interlockings, 12 were the same – 6 X 7 – and that from 4 pairs of rings, both the right and the left showed the same interlocking 6 X 7. On these interlockings we can also see that the interlockings of the one ring are always the mirror image of the corresponding one, as is shown in text fig. 17b and c. The interlocking 6 X 7 correlate absolutely. The same can be examined on the interlockings of the sparrow hawk *(Sperber)* (text fig. 19b, c), goshawk *(Hühnerhabicht)* and hen harrier *(Kornweihe).*

***Galliformes.***

***Cracidae,*** Hokkovögel***.***

According to GIEBEL:

*Penelope spec.*, Schakuhuhn: 14 scales.

*Crax alberti*, Hokko (text fig. 20).

A-type, right and left 14 scales +-scales: 1, 9; ⃝-scales: 7, 10.

Scales: wider than tall, strongly covered.

Ring: temporal notably wider than nasal, concave, steep, cartilage reaches far under.

According to GIEBEL:

*Crax alector*, Glattschnabelhokko *(black curassow).*

Left ring the same as under *C. alberti.*

Not mentioned in the text. This formation can clearly be seen from the drawing.

***Gallidae,*** eigentliche Hühner***.***

According to ALBERS:

*Meleagris gallopavo*, Truthuhn *(common turkey).*

A-type, 14 scales.

According to GIEBEL the same number of scales.

According to GIEBEL:

*Pavo cristatus*, gewöhnlicher Pfau *(common peacock).*

A-type, left 14 scales, +-scales: 1, 9; ⃝-scales: 6, 10.

*Gallus domesticus*, Haushuhn *(domestic fowl).*

1. A-type, right and left 14 scales, +-scales: 1, 9; ⃝-scales: 6, 10 (text fig. 21 a).

2.-5. The same as under 1. (text fig. 21 c-f).

6. A-type, right and left 15 scales, +-scales: 1, 10; ⃝-scales: 7, 11.

7. B-type, right and left 13 scales, +-scales: 1; ⃝-scales: 6 (text fig. 21 k).

8. A-type, right 15 scales and left 14 scales (text fig. 21 g);

Right: +-scales: 1, 10; ⃝-scales: 7, 11.

Left: +-scales: 1, 9; ⃝-scales: 6, 10.

9. A- type, right 14 scales and left 15 scales, +-scales: 1, 9;

Right: ⃝-scales: 7, 10.

Left: ⃝-scales: 6, 10.

10. A-type, right 16 scales and left 15 scales (fig. 21 l);

Right: +-scales: 1, 10; ⃝-scales: 7, 11.

Left: +-scales: 1, 9; ⃝-scales: 6, 10.

11. A-type, right 14 scales and left 15 scales, +-scales: 1, 10; ⃝-scales: 7, 11.

12. A-type, right and left 15 scales, +-scales: 1, 9; ⃝-scales: 6, 10.

13. A-type, right 15 scales, +-scales: 1, 9; ⃝-scales: 6, 10.

14. A-type, right 15 scales, +-scales: 1, 8; ⃝-scales: 7, 10 (text fig. 21 b).

15. A-type, left 13 scales, +-scales: 1, 8; ⃝-scales: 6, 9.

16a. A-type, right 13 scales, +-scales: 1, 8; ⃝-scales: 6, 9.

(Text fig. 21h, i).

16b. B-type, left 14 scales, +-scales: 1; ⃝-scales: 7.

Interlocked: 2. left and right, 3. right, 8. left, 10. left, 14. right: 6 X 7; 16a. right: 8 X 9;

10. right: 1 X 16, 10 X 11.

Scales: square shaped up to wider than tall, rounded, thin, very well covered, without medullary spaces.

Ring: flat

*Phasianus colchicus*, Edelfasan *(common pheasant).*

1. A-type, right and left 14 scales, +-scales: 1, 9;

Right: ⃝-scales: 6, 10;

Left: ⃝-scales:7, 10.

2. A-type, right and left 14 scales, +-scales: 1, 9; ⃝-scale: 7, 10.

Interlocked: 1. left 6 X 7.

According to GIEBEL: as per 2.

ALBERS also states A-type and 14 scales.

*Perdix perdix*, Rebhuhn *(partridge) (text fig. 22 a, b).*

A-type, right and left 14 scales, +-scales: 1, 9; ⃝-scales: 7, 10.

Interlocked: right 6 X 7 and 3 X 4.

According to GIEBEL: A-type, left 15 scales, +-scales: 1, 9; ⃝-scales: 5, 10.

*Lyrurus tetrix*, Birkhuhn *(black grouse).*

A-type, right 14 scales and left 15 scales, +-scales: 1, 9;

Right: ⃝-scale: 6, 10; left: ⃝-scale: 6, 11.

According to ALBERS: A-type, 14 scales.

GIEBEL counts only 11 scales.

*Tetrao urogallus*, Auerhuhn *(capercaillie).*

A-type, right 15 scales and left 14 scales, +-scales: 1, 9; ⃝-scales: 6, 10.

Interlocked: left 3 X 4 (with only a small overlap).

With regards to the formation of the scales and the ring of the above mentioned 4 representatives of the *Gallidae*, we can say the same as what was said under *Gallus domesticus*.

***Opisthocomidae,*** Schopfhühner***.***

*Opisthocomus hoazin*, Schopfhuhn *(hoatzin).*

Right and left 12 scales.

The formation of the scales could not be examined due to severe ruptures.

Text fig. 20-22b *Galliformes.*

Text fig. 20 *Crax alberti*, right ⅟₁; text fig. 21a-l. domestic fowl, a right, ⅟₁, b right, ⅟₁, c right scales 9, 10, ⁵∕₃, d left scales 6-10, ⁵∕₃, e left scales 6-8, ⁵∕₃, f right scales 8-10, ⁵∕₃, g left scales 8-10, ⁵∕₃, h right, ⅟₁,

i left, ⅟₁, k left, ⅟₁, l left, ⅟₁; text fig. 22a, b. partridge, right and left, ⅟₁.

The series of forms of the domestic fowl shows many remarkable things. I will only mention a few things about the interlocking at this stage as I will go into more detail regarding the aforementioned further below. Here too we can examine an accumulation of the same interlocking. All interlockings 9 X 10 are happening in the same way (text fig. 21b-d, f, g, l); of the 3 interlockings 6 X 7 only 2 correspond. Looking at the other representatives of the Hühnervögel *(Galliformes)*, we can see that the interlocking 6 X 7 of pheasant and partridge and 3 X 4 of partridge and capercaillie also coincide. The fact is that *Opisthocomus* with 12 scales is in remarkably stark contrast to the rest of the Galliformes.

***Gruiformes.***

***Rallidae,*** Rallen *(rails)****.***

*Rallus aquaticus*, Wasserralle *(water rail)* (text fig. 23).

A-type, 14 scales, +-scales: 1, 8; ⃝-scales: 6, 10.

*Crex crex*, Wiesenknarrer *(landrail)* (text fig. 24a, b).

A-type, right and left 13 scales, +-scales: 1, 7;

Right: ⃝-scales: 6, 9; left: ⃝-scales: 6, 8.

*Gallinula chloropus*, grünfüßiges Teichhuhn *(common moorhen)* (text fig. 25).

1. A-type, right and left 13 scales, +-scales: 1,8; ⃝-scales: 6, 9.

2. The same as under 1.

3. A-type, right 12 scales, left 13 scales;

Right: +-scales: 1, 7; ⃝-scales: 5, 8;

Left: +-scales: 1, 8; ⃝-scales: 6, 9.

4. A-type, right 14 scales, left 13 scales;

Right: +-scales: 1, 9; ⃝-scales: 7, 10;

Left: +-scales: 1, 8; ⃝-scales: 6, 9.

5. A-type, right 14 scales, left 13 scales;

Right: +-scales: 1, 8; ⃝-scales: 6, 9;

Left: +-scales: 1, 7; ⃝-scales: 5, 8.

6. A-type, right 13 scales, left 14 scales;

Right: +-scales: 1, 8; ⃝-scales: 6, 9;

Left: +-scales: 1, 9; ⃝-scales: 6, 10.

According to GIEBEL:

A-type, left 13 scales, +-scales: 1, 8; ⃝-scales: 6, 9.

The formation of the scales is not mentioned in the text. Based on the drawing we can see the above mentioned, although the ausgezeichneten scales are not marked as such.

*Fulica atra*, Bleßhuhn *(Eurasian coot).*

A-type, right 13 scales, left 14 scales, +-scales: 1, 8; ⃝-scales: 6, 9.

According to GIEBEL:

A-type, right 12 scales, left 13 scales, +-scales: 1, 8; ⃝-scales: 6, 9.

According to text: 12: 1, 6; 5, 8. 13: 1, 7; 6, 9.

Again counted in the incorrect sense. The corrected numbers match my own findings.

The following common denominators for the aforementioned representatives of the *Rallidae* are:

Scales: wider than tall up to square shaped, nasal almost hexagonal, strongly covered.

Ring: flat (for *Crex* ascending concave).

***Gruidae,*** Kraniche *(cranes)****.***

According to GIEBEL:

*Grus grus,* grauer Kranich *(common crane).*

A-type, left 15 scales, +-scales: 1, 9; ⃝-scales: 5, 11.

Only drawing available, from which the above-mentioned data can clearly be extracted.

***Dicholophidae,*** Schlangenstörche***.***

*Cariama cristata,* Seriema (text fig. 26).

A-type, right and left 14 scales, +-scales: 1, 9; ⃝-scales: 6, 11.

Interlocked: left 8 X 9, 10 X 11.

Scales: taller than wide, hardly covering, many medullary spaces.

Ring: on the outside slightly convex, on the inside concave; therefore erect. The convex part lies above the cartilage.

***Otitidae,*** Trappen *(bustards)****.***

According to GIEBEL:

*Otis tarda,* Großtrappe *(giant bustard).*

1. A-type, right and left 15 scales.

2. A-type, right 13 scales and left 15 scales.

***Rhinochetidae,*** Rallenkraniche *(limpkins)****.***

*Rhinochetus jubatus,* Kagu *(kagu).*

A-type, right and left 15 scales, +-scales: 1, 9; ⃝-scales: 5, 11.

Scales: taller than wide, hardly covering, distally well covered.

Ring: nasally notable narrower than temporally, proximally slightly convex, distally strongly concave, nasally less concave, cartilage reaches far under.

Text fig. 23-27. *Gruiformes.*

Text fig. 23 Wasserralle *(water rail)*, right, ⁵∕₂; text fig. 24a, b. Wiesenknarrer *(landrail),* right and left, ³∕₂; text fig. 25. Grünfüßiges Teichhuhn *(common moorhen),* left, ⁵∕₂; text fig. 26. Seriema, left, ³∕₂; text fig. 27. Kagu, right, ³∕₂.

***Charadriiformes.***

***Charadriidae,*** Regenpfeifer *(plover)****.***

According to GIEBEL:

*Charadrius pluvialis*, Goldregenpfeifer.

A-type, right 15 scales, +-scales: 1, 9; ⃝-scales: 5, 11.

According to ALBERS also A-type and 15 scales.

*Vanellus vanellus*, Kiebitz *(lapwing)* (text fig. 28).

A-type, right and left 15 scales, +-scales: 1, 9; ⃝-scales: 5, 11.

Scales: wider than tall, well covered, partly curved.

Ring: concave, flat.

According to ALBERS: A-type, 15 scales; GIEBEL also counts 15 scales.

According to GIEBEL:

*Haematopus ostralegus*, Austernfischer *(oystercatcher)*.

A-type, right 15 scales, +-scales: 1, 9; ⃝-scales: 5, 11.

According to GIEBEL and ALBERS:

*Pavonella pugnax*, Kampfläufer *(ruff):* 15 scales.

According to GIEBEL:

*Phalaropus lobatus*, Wassertreter*:* 15 scales.

*Scolopax rusticola*, Waldschnepfe *(woodcock).*

A-type, right and left 15 scales, +-scales: 1, 9; ⃝-scales: 5, 11.

Scales: wider than tall, well covered, rounded.

Ring: flat.

According to GIEBEL:

A-type, left 15 scales, +-scales: 1, 8; ⃝-scales: 4, 11.

According to GIEBEL:

*Numenius arquatus*, Brachvogel (curlew).

A-type, left 14 scales, +-scales: 1, 8; ⃝-scales: 5, 10.

***Oedicnemidae,*** Dickfüße *(stone-plovers)****.***

According to GIEBEL:

*Oedicnemus oedicnemus*, Triel.

A-type, right and left 15 scales, +-scales: 1, 9; ⃝-scales: 5, 11.

Nothing is mentioned in the text. Based on the pictures, the above-mentioned formation can be seen if counted the correct way, this means one line of the one ring is to be removed and therefore also the redundant minus sign. This accidentally drawn line is probably the reason why GIEBEL counted an additional scale in this ring, therefore 16 according to the table.

***Laridae,*** Möwen *(seagulls)****.***

According to GIEBEL:

*Larus glaucus*, Eismöwe *(burgomeister gull):* right 16 scales, left 17 scales.

*Larus argentatus*, Silbermöwe *(common European gull).*

1. A-type, right and left 15 scales, +-scales: 1, 9; ⃝-scales: 5, 11.

2. The same as under 1.

3. A-type, right 16 scales, +-scales: 1, 9; ⃝-scales: 5, 12.

4. A-type, right and left 14 scales, +-scales: 1, 8; ⃝-scales: 4, 10.

5. A-type, right 16 scales, left 15 scales, +-scales: 1, 9;

Right: ⃝-scales: 5, 12; left: ⃝-scales: 5, 11.

Scales: wider than tall up to square shaped, strongly covered.

Ring: ascending.

According to GIEBEL: A-type, left 15 scales, +-scales: 1, 9; ⃝-scales: 5, 11.

*Larus canus*, Sturmmöwe *(mew gull).*

1. A-type, right and left 15 scales, +-scales: 1, 9; ⃝-scales: 5, 11.

2. A-type, right 15 scales, +-scales: 1, 9; ⃝-scales: 5, 11.

Scales: wider than tall, strongly covered, nasally the edges are drawn out to the tips.

Ring: ascending.

According to GIEBEL: A-type, left 15 scales, +-scales: 1, 9; ⃝-scales: 5, 11.

Möwe *(gull).*

Class and species are unknown to me.

(Location: St. Cruz- Islands.)

A-type, right and left 15 scales;

Right: +-scales: 1, 8; ⃝-scales: 7, 9;

Left: +-scales: 1, 6; ⃝-scales: 5, 7.

Scales: temporally almost double as tall than wide, nasally almost square shaped, well covered, firmly connected to each other.

Ring: steep.

*Larus ridibundas*, Lachmöwe *(black-headed gull).*

A-type, right and left 15 scales, +-scales: 1, 9; ⃝-scales: 5, 11.

Scales: wider than tall, well covered.

Ring: flat, inner edge steep.

According to GIEBEL: 15 scales.

According to GIEBEL:

*Rissa tridactyla*, Stummelmöwe*:* 15 scales.

According to ALBERS:

*Sterna fluviatilis*, Flußseeschwalbe.

A-type, 15 scales.

GIEBEL also counts 15 scales.

***Alcidae,*** Flügeltaucher***.***

According to GIEBEL:

*Alca torda*, Tordalk *(razorbill).*

A-type, right 13 scales, +-scales: 1, 8; ⃝-scales: 4, 12.

According to GIEBEL and ALBERS:

*Alle alle,* Krabbentaucher *(little auk):* 13 scales.

According to ALBERS:

*Uria grille,* Teiste.

A-type, 13 scales.

GIEBEL also counts 13 scales.

According to ALBERS:

*Uria Troile,* Trottellumme (*guillemot).*

A-type, 13 scales.

GIEBEL also counts 13 scales.

According to ALBERS:

*Fratercula arctica,* Papageitaucher (*Atlantic puffin).*

A-type, 13 scales.

GIEBEL also counts 13 scales.

A note made by LEUCKART contradicts the above-mentioned number. He writes: “Their number usually fluctuates between 12 and 15. Only *Alca arctica* (Fr. a.) shows double the amount (30) as the individual scales are separated into a smaller front half and a bigger rear half” (as per Bern. Page 552).

***Columbidae,*** Tauben (*doves)****.***

According to GIEBEL:

*Streptopelia risoria*, Lachtaube *(ringneck dove).*

A-type, right 10 scales and left 11 scales;

Right: +-scales: 1, 6; ⃝-scales: 4, 7;

Left: +-scales: 1, 7; ⃝-scales: 4, 8.

According to GIEBEL:

*Goura coronata*, gewöhnliche Krontaube *(common crowned pigeon).*

A-type, right 11 scales, +-scales: 1, 7; ⃝-scales: 4,8.

He states the following regarding the common crowned pigeon: 1, 6 and 5, 9. However, the picture clearly shows that he counted incorrectly. The corrected results correlate to the results of the other doves.

*Columba Palumbus*, Ringeltaube *(wood pigeon).*

1. A-type, right and left 11 scales, +-scales: 1, 7; ⃝-scales: 4, 8.

2. A-type, right 12 scales and left 11 scales;

Right: +-scales: 1, 8; ⃝-scales: 5, 9;

Text fig. 29a, b.

Left: +-scales: 1, 7; ⃝-scales: 4, 8.

Scales: wider than tall, some in hexagonal shape, the others rounded, strongly covered.

Ring: ascending, on the inner edge steep, concave, the narrowest: nasoventrally, at that point hexagon shape of the scales (hight: width [of the scales] = 1:2).

What has been said about the scales and ring also applies to the following doves.

The anomaly in the kind of coverage under 2. right is due to the fact that a scale has been inserted in the ventral-temporal part, which means that the 4^th^ scale is smaller than its neighbouring scales. Together with scale 3, one could assume it is one scale. According to that the following kind of coverage exists: 1, 7; 4, 8, thus the typical one for this family.

Haustaube *(domestic pigeon).*

1. A-type, right and left 11 scales, +-scales: 1, 7; ⃝-scales: 4, 8.

2.-4. The same as under 1.

5. A-type, right 12 scales and left 11 scales;

Right: +-scales: 1, 8; ⃝-scales: 5, 9;

Left: +-scales: 1, 7; ⃝-scales: 4, 8.

Interlocked: 4. Right 4 X 5 (text fig. 30a).

Scale 1 under 5. Right is particularly small compared to its neighbouring scales. At that point 3 scales cover each other as follows: 1 over 2 and 12, 2 over 3 and 11. If scale number 1 would not be there, then the typical kind of coverage for this family would apply. This case is similar to the one for the wood pigeon.

Turtur turtur, gemeine Turteltaube.

A-type, right and left 11 scales, +-scales: 1, 7; ⃝-scales: 4, 8.

In this order the following 3 groups can clearly be defined based on the number of scales: the *Columbidae* with 11 scales, the *Alcidae* with 13 scales and the remaining families with continuously 15 scales. Apart from minor deviations, the rings with 15 scales have the formation 1, 9; 5, 11, in accordance with the *Anseridae*.

***Ouculiformes.***

***Cuculidae,*** echte Kuckucke *(cuckoos)****.***

*Cuculus canorus,* gemeiner Kuckuck *(common cuckoo)* (text fig. 31).

A-type, left 12 scales, +-scales: 1, 8; ⃝-scales: 5, 10.

Scales: wider than tall, well covered.

Ring: flat.

GIEBEL states the same of the right ring (picture upside down).

***Psittacidae,*** eigentliche Papageien.

*Ara macao,* Arakanga *(scarlet macaw)* (text fig. 32a, b).

A-type, right 12 scales and left 11 scales;

Right: +-scales: 1, 7; ⃝-scales: 5, 9;

Left: +-scales: 1, 6; ⃝-scales: 4, 8.

Interlocked: right 11 X 12; left 4 X 5.

Scales: wider than tall, well covered.

Ring: ascending.

Papagei (class and species unknown to me).

A-type, right and left 12 scales, +-scales: 1, 7; ⃝-scales: 4, 9.

Interlocked: right and left 5 X 6.

Scales and ring as per *Ara macao.*

According to GIEBEL:

*Psittacus sinensis.*

A-type, left 13 scales, +-scales: 1, 8; ⃝-scales:5, 11.

According to ALBERS:

*Psittacus erithacus,* Jako.

B-type, 14 scales.

GIEBEL also counts 14 scales.

According to ALBERS:

*Psittacus omocephalus.*

A-type, 12 scales.

According to GIEBEL:

*Psittacus christatus.*

A-type, left 12 scales, +-scales: 1, 6; ⃝-scales: 4, 9.

According to GIEBEL:

*Psittacus dominicensis.*

A-type, left 12 scales, +-scales: 1, 7; ⃝-scales: 4, 9.

According to text: left: 1, 7; 3, 10. Therefore counted the incorrect way. Once rectified, the formation matches the above-mentioned results.

According to GIEBEL:

*Psittacus solstitialis.*

A-type, left 12 scales, +-scales: 1, 7; ⃝-scales: 4, 9.

According to GIEBEL also *Psittacus ochrocephalus*, *Ps. auricapillus* and *Ps. pertinax* also have 12 scales.

Text fig. 28-32b. *Charadriiformes. Cuculiformes.*

Text fig. 28. Kiebitz *(lapwing)*, right, ³∕₂; text fig. 29a, b. Ringeltaube *(wood pigeon)*, right and left, ³∕₂; text fig. 30a. Haustaube *(domestic pigeon),* right scales 3-5, ⁵∕₂; text fig. 30b. Haustaube *(domestic pigeon),* right scales 12, 1, 2, 3, ⁵∕₂; text fig. 31. Gemeiner Kuckuck *(common cuckoo)*, left, ³∕₂; text fig. 32a, b. Arakanga *(scarlet macaw),* right and left, ³∕₂.

This order is relatively consistent. Noteworthy is the fact that according to ALBERS, Jako is supposed to have b-type; a bigger number of scales, which was verified by GIEBEL, sets Jako apart from the others. As noted many times before, we again find the same interlocking in the right and left eye of a representative as well as the same way of interlocking (Papagei).

***Coraciiformes.***

***Coraciidae,*** eigentliche Raken *(rollers)****.***

According to GIEBEL:

*Coracias garrulus,* Blaurake *(common roller)*.

A-type, left 13 scales, +-scales: 1, 8; ⃝-scales: 5, 10.

According to text: 1, 7; 5, 10. However, looking at the drawing it is obviously the above-mentioned order.

***Alcedinidae,*** Eisvögel *(kingfishers)****.***

According to GIEBEL:

*Alcedo ispida,* gewöhnlicher Eisvogel.

A-type, left 13 scales, +-scales: 1, 8; ⃝-scales: 5, 10.

***Upupidae,*** Hopfe (hoopoes*)****.***

According to ALBERS:

*Dichocerus bicornis,* Doppelhornvogel *(great Indian hornbill).*

B-type, 13 scales.

GIEBEL counts the same number of scales.

*Upupa epops,* Wiedehopf *(common Eurasian hoopoe) (text fig. 33a, b).*

A-type, right an left 13 scales;

Right: +-scales: 1, 8; ⃝-scales: 5, 10;

Left: +-scales: 1, 8, 11; ⃝-scales: 5, 10, 12;

Scales: wider than tall, well covered, rounded.

Ring: flat, concave.

Text fig. 33a, b, 38, 39. *Coraciiformes* I.

Text fig. 33a, b. Wiedehopf *(common Eurasian hoopoe)*, right and left ³∕₂; text fig. 38. Mauersegler *(common swift)*, right, ³∕₂; text fig. 39 Buntspecht *(spotted woodpecker)*, right, ³∕₂.

Here we have the very rare case that a ring has 3 pairs of ausgezeichnete scales, whereby 2 pairs match the ones from the other ring.

***Strigidae,*** Eulen *(owls)****.***

*Strix flammea,* Schleierkauz *(barn owl).*

1. A-type, right and left 16 scales;

Right: +-scales: 1, 14; ⃝-scales: 8, 15;

Left: +-scales: 1, 13; ⃝-scales: 8, 15.

Interlocked: left 7 X 8, 13 X 14, 15 X 16;

Right 7 X 8.

2a. A-type, right 16 scales, +-scales: 1, 14; ⃝-scales: 8, 15.

b. B-type, left 16 scales, +-scales: 1; ⃝-scales: 8 (text fig. 34a, b).

Interlocked: right 7 X 8 (little interlocking)

Scales: taller than wide, little covered many medullary spaces.

Ring: tube shaped, concave.

*Athene noctua,* Steinkauz *(little owl)*.

1. B-type, right 15 scales and left 14 scales, +-scales: 1;

Right: ⃝-scales: 6; left: ⃝-scales: 5.

2. B-type, right 15 scales and left 14 scales, +-scales: 1; ⃝-scales: 5.

Scales: taller than wide, partly double as tall than wide. Tapering shape after the inner edge of the ring, little covered.

Ring: tube shaped, concave.

According to GIEBEL:

*Glaucidium passerinum,* Zwergeule *(Eurasian pygmy owl).*

B-type, right 14 scales and left 15 scales, +-scales: 1; ⃝-scales: 5.

According to text: 1, 11. Counted the incorrect way.

*Syrnium aluco,* Waldkauz *(tawny owl).*

1. B-type, right 18 scales and left 15 scales;

Right: +-scales: 1; ⃝-scales: 7;

Left: +-scales: 1; ⃝-scales: 6.

2. B-type, right and left 15 scales, +-scales: 1; ⃝-scales: 6.

3. B-type, right 16 scales, +-scales: 1; ⃝-scales: 5.

4. B-type, right 15 scales, +-scales: 1; ⃝-scales: 6.

Interlocked: 1. right, 2. right and left, 4. left: 1 X 2 (text fig. 35).

Scales: taller than wide, some up to 3 times as tall than wide, many medullary spaces, little covered.

Ring: tube shaped, strongly concave.

According to EDINGER:

*Syrnium uralense,* Habichtseule *(ural owl)*: 15 scales and

*Scops scops,* Zwergohreule *(scops owl):* 14 scales and

Nyctea Nyctea, Schnee-Eule *(snowy owl):* 17 scales.

*Bubo bubo,* Uhu *(eagle owl).*

1. B-type, right 15 scales and left 16 scales, +-scales: 1; ⃝-scales: 6.

2. B-type, right and left 15 scales, +-scales: 1; ⃝-scales: 6 (text fig. 36a).

3. B-type, left 15 scales, +-scales: 1; ⃝-scales: 6.

Interlocked: 1. right and left 1 X 2 (text fig. 36b, c).

Scales: taller than wide, some 3-4 times as tall than wide, many and big medullary spaces, little covered.

Ring: most completed tube shaped, oval, nasally stronger concave than temporally, consequently strongly asymmetrical.

According to ALBERS: B-type, 15 scales.

GIEBEL also counts 15 scales.

*Asio otus,* Waldohreule *(long-eared owl)* (text fig. 37a-g).

1. B-type, right and left 15 scales, +-scales: 1; ⃝-scales: 6.

2., 3. The same as under 1. (text fig. 37a).

4. B-type, right 15 scales and left 16 scales, +-scales: 1;

Right: ⃝-scales: 6; left: ⃝-scales: 7.

5. B-type, right 16 scales and left 15 scales, +-scales: 1;

Right: ⃝-scales: 7; left: ⃝-scales: 6.

6. B-type, right and left 15 scales, +-scales: 1;

Right: ⃝-scales: 6; left: ⃝-scales: 7.

7. B-type, right 18 scales and left 16 scales, +-scales: 1;

Right: ⃝-scales: 8; left: ⃝-scales: 6.

8. B-type, right 15 scales, +-scales: 1; left: ⃝-scales: 7.

9. B-type, left 15 scales, +-scales: 1; ⃝-scales: 6.

10. B-type, right and left 14 scales, +-scales: 1; ⃝-scales: 6.

Interlocked: 2. right, 6. left: 6 X 7 (text fig. 37b, c); 2. right: 1 X 2; 2. left: 9 X 10 (text fig. 37d, f);

1. right: 1 X 2 X 3, 8 X 9 (fig. 37 e, g).

Scales: taller than wide, maximum: 3 times as tall than wide, many medullary spaces, little covered.

Ring: tube shaped, temporally steeper than nasally.

According to GIEBEL: A-type, 16 scales.

According to GIEBEL:

*Asio accipitrinus,* Sumpfeule *(short-eared owl).*

B-type, right 16 scales, +-scales: 1; ⃝-scales: 7.

According to text: 1, 11; as per *Glaucidium passerinum.*

***Caprimulgidae,*** Ziegenmelker *(nightjar)****.***

According to GIEBEL:

*Caprimulgus europaeus,* Nachtschwalbe *(European nightjar).*

A-type, right 15 scales: +-scales: 1, 9; ⃝-scales: 5, 11.

In the picture one minus sign too many.

***Cypselidae,*** Segler.

*Cypselus apus,* Mauersegler *(swift)* (text fig. 38, as per page 542).

1. A-type, right and left 15 scales, +-scales: 1, 9; ⃝-scales: 6, 11.

2. The same as under 1.

3. A-type, right 16 scales and left 15 scales;

Right: +-scales: 1, 10; ⃝-scales: 6, 12;

Left: +-scales: 1, 9; ⃝-scales: 6, 11.

Scales: almost square shaped, rounded, strongly covered.

Ring: steep, temporally steeper than nasally, strongly concave.

GIEBEL also counts 15 scales.

***Picidae,*** Spechte *(woodpeckers).*

According to GIEBEL:

*Picus martius,* Schwarzspecht *(black woodpecker).*

A-type, right and left 13 scales, +-scales: 1, 8; ⃝-scales: 5, 10.

Not mentioned in the text. The labels for the right ring in the drawing are missing; however, the ausgezeichneten scales are identifiable as those above.

*Dendrocopus,* Buntspecht *(spotted woodpecker)* (text fig. 39, as per page 542).

1. A-type, right and left 13 scales, +-scales: 1, 8; ⃝-scales: 5, 10.

2., 3. The same as under 1.

4. A-type, right 12 scales, +-scales: 1, 8; ⃝-scales: 5, 10.

Scales: wider than tall up to square shaped, well covered, many small medullary spaces, firmly fused to one another.

Ring: flat, the cartilage reaches under up to half of it, slightly convex.

According to GIEBEL:

*Gecinus viridis,* Grünspecht *(green woodpecker).*

A-type, right 12 scales, +-scales: 1, 8; ⃝-scales: 6, 10.

According to text: 1, 6; 4, 8. Here too, I assume that GIEBEL counted the wrong way, as the temporal side is narrower than the nasal side. The corrected data corresponds much more with the known facts that were clearly identifiable as per above.

*Gecinus canus,* Grauspecht *(gray woodpecker).*

A-type, right 13 scales, +-scales: 1, 8; ⃝-scales: 6, 10.

Scales: taller than wide up to square shaped, well covered.

Ring: flat.

Text fig. 34a-37g. *Coraciiformes* II.

Text fig. 34a, b. Schleierkauz *(barn owl),* right and left, ⁴∕₃;

text fig. 35. Waldkauz *(tawny owl)*, left scales 1, 2, ⅟₁;

text fig. 36a-c. Uhu *(eagle owl)*, ⅟₁, a right, b right scales 1, 2, 15, c left scales 16, 1, 2;

text fig. 37a-g. Waldohreule *(long-eared owl),* ⁴∕₃, a right, b right scales 6, 7, c left scales 5, 6, 7, d right scales 2, 1, 15, e right scales 1, 2, 3, f left scales 9, 10, g right scales 8, 9, 10.

3 groups can clearly be distinguished. Firstly Raken *(rollers),* Eisvögel *(kingfisher)*, Hopfe *(hoopoe)* and Spechte *(woodpecker)* with A-type and 13 scales; secondly Ziegenmelker *(nightjar)* and Segler *(Cypselidae)* also with A-type and 15 scales and then thirdly Eulen *(owls)* with B-type and on average 15 scales. Some findings differ in regard to the type; more about that will follow (page 568). Here again an accumulation of the same interlockings. We can find the interlocking 7 X 8 three times in the 4 rings of the Schleiereule *(barn owl)* and it always occurs in the same way. The Waldkauz *(tawny owl)* only shows the interlocking 1 X 2 but four times, here too always in the same way. The interlockings of the Uhu *(eagle owl)* are insofar remarkable as scale 1 reaches to some extent twice to scale 2 and scale 2 only once, and that in both rings. The same kind of coverage can be seen again on the right and left ring. Similar results cannot be found on a Waldohreule (long-eared owl).

***Passeriformes.***

***Muscicapidae,*** Fliegenfänger *(flycatcher)****.***

*Phylloscopus trochilus,* Fitislaubsänger *(willow warbler)*.

A-type, right 14 scales, +-scales: 1, 8; ⃝-scales: 6, 10.

Scales: wider than tall, rounded, well to strongly covered.

Ring: flat.

*Sylvia borin,* Gartengrasmücke *(garden warbler)* (text fig. 40).

A-type, right and left 14 scales, +-scales: 1, 8; ⃝-scales: 5, 10.

Scales: wider than tall up to square shaped, nasally the sides are drawn out to an irregular, spikey margin, well covered.

Ring: flat, slightly concave.

*Sylvia curruca,* Zaungrasmücke *(lesser whitethroat)* (text fig. 41a, b).

A-type, right 16 scales and left 13 scales;

Right: +-scales: 1, 9; ⃝-scales: 6, 11;

Left: +-scales: 1, 8; ⃝-scales: 6, 10.

Scales: wider than tall, partially square shaped, slightly rounded.

Ring: flat.

*Turdus viscivorus,* Misteldrossel *(mistle thrush)* (text fig. 42).

A-type, right and left 14 scales, +-scales: 1, 8; ⃝-scales: 5, 10.

*Turdus pilaris,* Wacholderdrossel *(fieldfare)*.

1. A-type, right and left 14 scales, +-scales: 1, 8; ⃝-scales: 5, 10.

2. The same as under 1.

Interlocked: 2. Right and left: 5 X 6; 2. Left 14 X 1.

*Turdus merula,* Amsel *(blackbird)*.

1. A-type, right and left 14 scales, +-scales: 1, 8; ⃝-scales: 5, 10.

2. A-type, right and left 14 scales, +-scales: 1, 8;

Right: ⃝-scales: 5, 10; left: ⃝-scales: 5, 11.

1. A-type, right and left 14 scales, +-scales: 1, 8;

Right: ⃝-scales: 5, 10; left: ⃝-scales: 5, 9.

1. A-type, right 13 scales and left 14 scales;

Right: +-scales: 1, 7; ⃝-scales: 5, 9;

Left: +-scales: 1, 8; ⃝-scales: 5, 10.

Interlocked: 4. Right: 6 X 7.

The following applies for all above mentioned representatives of the genus *Turdus* regarding their scales and rings:

Scales: wider than tall, rounded (*T. viscivorus*: the edges are drawn out to an irregular, spikey margin), fused on the outer margin, well covered.

Ring: flat.

*Phoenicurus phoenicurus,* Gartenrotschwanz *(redstart)*.

A-type, right and left 14 scales, +-scales: 1, 8; ⃝-scales: 5, 10.

Scales: wider than tall, rounded, well covered.

Ring: ascending, concave.

*Erithacus rubecula,* Rotkehlchen *(robbin)*.

A-type, right and left 14 scales, +-scales: 1, 8; ⃝-scales: 5, 10.

Scales: wider than tall, rounded, nasal laterally with spikes, well covered.

Ring: flat.

***Ampelidae,*** Seidenschwänze *(waxwings)****.***

According to GIEBEL:

*Bombycilla garrulus,* gemeiner Seidenschwanz *(bohemian waxwing)*.

A-type, left 14 scales, +-scales: 1, 8; ⃝-scales: 5, 10.

Besides the mention of the number of scales, only the drawing is available, which clearly shows the above-mentioned formation.

***Laniidae,*** Würger *(shrikes)****.***

According to GIEBEL:

*Lanius collurio,* rotrückiger Würger *(red-backed shrike)*: 14 scales.

***Corvidae,*** Rabenvögel *(corvids)****.***

According to GIEBEL:

*Corvus corax,* Kolkrabe *(common raven)*.

A-type, left 14 scales, +-scales: 1, 8; ⃝-scales: 5, 10.

According to ALBERS: A-type, 14 scales.

*Corvus corone,* Rabenkrähe *(carrion crow)*.

1. A-type, right and left 14 scales, +-scales: 1, 8; ⃝-scales: 5, 10.

2. The same as under 1. (fig. 43a).

Scales: somewhat wider than tall, almost square shaped, slightly rounded, all scales are structured considerably uniform, well to strongly covered, joining on the outer edge.

Ring: ascending, concave, on the inner edge steep.

Interlocked: 2. Right 7 X 8 (fig. 43 b).

In the left ring under 1., on the border of the 7^th^ and 8^th^ scale, there is another little scale developed, which covers both scales; I did not give her a number (fig. 43c).

According to ALBERS: A-type, 14 scales; according to GIEBEL also 14 scales.

According to GIEBEL:

*Corvus cornix,* Nebelkrähe *(hooded crow)*.

A-type, 15 scales.

GIEBEL also counts 15 scales.

*Pica pica,* europäische Elster *(Eurasian magpie)*.

1. A-type, right and left 13 scales;

Right: +-scales: 1, 7; ⃝-scales: 5, 10;

Left: +-scales: 1, 8; ⃝-scales: 6, 11.

2. A-type, right 14 scales, +-scales: 1, 8; ⃝-scales: 5, 10.

Interlocked: 2. 2 X 3.

Scales: wider than tall, rounded, also taller than wide (size varies a lot), partially fused on the outer margin.

Ring: flat.

*Garrulus glandarius,* Eichelhäher *(jay)*.

1. A-type, right and left 14 scales, +-scales: 1, 8; ⃝-scales: 5, 10.

2. A-type, right 13 scales and left 14 scales, +-scales: 1, 8; ⃝-scales: 5, 10.

3. A-type, right 14 scales and left 13 scales;

Right: +-scales: 1, 8; ⃝-scales: 5, 10;

Left: +-scales: 1, 7; ⃝-scales: 5, 10.

4. A-type, right 14 scales, +-scales: 1, 8; ⃝-scales: 5, 10.

5. A-type, right 14 scales and left 13 scales, +-scales: 1, 8; ⃝-scales: 5, 10.

Scales: wider than tall, rounded, the sides partially drawn out in peaks, well covered.

Ring: ascending up to steep, concave.

***Oriolidae,*** Pirole *(orioles)****.***

According to GIEBEL:

*Oriolus oriolus,* Pirol *(oriole)*.

Right 13 scales and left 14 scales.

ALBERS counts 13 scales.

***Sturnidae,*** Stare *(starlings)****.***

*Sturnus vulgaris,* Star *(starling)*.

1. A-type, right and left 14 scales, +-scales: 1, 8; ⃝-scales: 6, 9.

2. A-type, right and left 14 scales;

Right: +-scales: 1, 8; ⃝-scales: 6, 9;

Left: +-scales: 1, 9; ⃝-scales: 7, 10.

Scales: wider than tall, well covered.

Ring: flat.

***Fringillidae,*** Finken *(fringillids)****.***

According to GIEBEL:

*Passerina nivalis,* Schneeammer *(snow bunting)*.

A-type, left 14 scales, +-scales: 1, 8; ⃝-scales: 5, 10.

*Emberiza occothrau,* Goldammer *(yellow bunting)*.

A-type, right and left 14 scales, +-scales: 1, 8; ⃝-scales: 5, 10.

Scales: wider than tall, fused on the outer margin, rounded, well covered.

Ring: ascending.

*Passer domesticus,* Haussperling *(house sparrow)* (text fig. 44).

1. A-type, right and left 14 scales, +-scales: 1, 8; ⃝-scales: 6, 9.

2. The same as under 1.

Scales: wider than tall, rounded, fused on the outer margin (as per SLONAKER), well covered.

Ring: flat.

*Fringilla montifringilla,* Bergfink *(mountain finch)*.

1. A-type, right and left 14 scales, +-scales: 1, 8; ⃝-scales: 5, 10.

2. A-type, left 15 scales, +-scales: 1, 8; ⃝-scales: 5, 10.

Interlocked: 1. Right and left 1 X 14, 12 X 13; 2. 1 X 15.

Scales: almost square shaped, well covered.

Ring: slightly ascending.

*Pyrrhula pyrrhula,* Gimpel *(bullfinch)*.

1. A-type, right and left 14 scales, +-scales: 1, 8;

Right: ⃝-scales: 5, 10; left: ⃝-scales: 6, 11.

2. A-type, right and left 14 scales, +-scales: 1, 8; ⃝-scales: 6, 10.

Interlocked: 1. Left 6 X 7.

Scales: wider than tall, rounded, well covered, partially fused on the outer margin.

Ring: flat.

According to GIEBEL:

*Carduelus spinus,* Zeisig *(common siskin)*: 14 scales.

According to GIEBEL:

*Chloris chloris,* Grünfink *(common greenfinch)*: 14 scales.

In the text there is no mention regarding formation of the scales; the picture is indecipherable.

Text fig. 40-46b. *Passeriformes.*

Text fig. 40. Gartengrasmücke *(garden warbler),* right, ⁵∕₂;

Text fig. 41a, b. Zaungrasmücke *(lesser whitethroat),* right and left, ⁵∕₂;

Text fig. 42. Misteldrossel *(mistle thrush),* right, ³∕₂;

Text fig. 43a-c. Rabenkrähe *(carrion crow)*, a right, ³∕₂, b right scales 6-9, ⁵∕₂, c left scales 6-9, ⁵∕₂;

Text fig. 44. Haussperling *(house sparrow)*, right, ³∕₂;

Text fig. 45. Kernbeißer *(grosbeak)*, left, ⁵∕₂;

Text fig. 46a, b. Feldlerche *(European skylark)*, right and left, ⁵∕₂.

*Coccothraustes occothraustes,* Kernbeißer *(grosbeak)* (text fig. 45).

1. A-type, right and left 14 scales, +-scales: 1, 8; ⃝-scales: 5, 10.

2. The same as under 1.

3. A-type, left 15 scales, +-scales: 1, 8; ⃝-scales: 5, 11.

Interlocked: 1. Left 8 X 9; 3. Left 1 X 15, 6 X 7 X 8.

Scales: wider than tall, spikes have partially formed on the sides, well covered.

Ring: flat.

*Alauda arvensis,* Feldlerche *(European skylark)* (text fig. 46a, b).

B-type, right and left 14 scales, +-scales: 1; ⃝-scales: 6.

Interlocked: left 1 X 2.

Scales: wider than tall, rounded, well covered.

Ring: ascending.

A-type, a scale count of 14 and the formation 1, 8; 5, 10 are characteristics of the passerine birds; although not exclusive characteristics, they are more or less predominant. Here again both cases which show the same interlocking on the right and left side also show the same way of interlocking (fieldfare, mountain finch).

**General overview of the examined material**

Firstly, I will mention the features of the above findings.

Own examinations: ring pairs 137, individual rings 29. A total of 303 rings.

These rings are spread over 74 different species. One ring pair is without the mention of the positioning of its ausgezeichnete scales.

Examinations of other authors: ring pairs 11, individual rings 135. A total of 157 rings.

Those rings are spread over 112 different species, whereof 28 species are represented in my findings. Of the 157 rings, 79 are without mention of the formation of its scales.

Total examination: ring pairs 148, Individual rings 164. This makes a total of 460 rings.

They are spread over 158 different species. Of 148 ring pairs, 43 have a different scale count in the right eye than in the left one.

Based on these findings, we primarily want to establish the nature of the scales. After all the question is: Is the scale count and formation of the scales a random phenomenon or are both occurrences based on certain rules or based only on regularities? This is the primary question, from which further questions can be derived, which can only be answered incorrectly based on the examination of limited and very differing material as one will easily be convinced based on the following. Because we had to make do with material which happened to fall into our hands, it was impossible to make general statements at various points. Of the 14 orders 4 alone *(Ratites, Colymbiformes, Sphenisciformes, Procellariiformes*) have to stand aside due to very sparse findings that become almost insignificant to form general conclusions, whilst 1 order *(Tinamiformes)* is not represented at all. My findings of the plovers would tell us that the numbers 11 and 15 are typical for this order; but because I did not have 1 specimen available to me from the entire suborder of the *Alcidae*, which consistently showed a scale count of 13 as per GIEBEL, we would have a completely incorrect picture of this order without the findings from GIEBEL. Therefore, even if we have plenty of material available for one group, we still have to be careful in making conclusions or generalizations.

**3. The scales of the sclerotic ring**

**Form**

As many rings as I examined, I always found the ring to be made up of individual elements, the scales. The consistency of the scales varies from species to species and, as one can expect, in the individual stages of age. There are rings where the elements can be easily separated – this was the case for in the majority of cases - whereas a separation on others is impossible and only under great difficulty is it possible to determine the scales and their formation as such (woodpeckers, penguins and finally all specimens that are only a few days old). That the species possesses medullary spaces in varying formations in the scales was already mentioned in detail by CANFIELD. The scales of the owls, especially of the eagle owl, have noticeable big spaces.

There are scales that can be between thin-flexible and hard-brittle or between wider than tall and taller than wide (the height is the proximodistal orientation, the width the circular orientation). Just as variable is the shape of each scale; trapezoidal, hexagonal and narrow are recognizable as the basic shapes, between which all transitions are possible. Due to the fact that the size and shape of the scales vary greatly even within a ring, one cannot even attribute the sole possession of these characteristics to even small groups. The trapezoidal shape can be found in a parrot, the hexagonal shape in a common moorhen, especially in the nasally positioned scales; the narrow shape can be found the best in an eagle owl, just to pick a few of the typical examples. Naturally, these shapes are not to be understood as exactly geometrical, but only as an approximation. The edges of the scales are often more or less rounded, often extended to a hexagonal shape due to so called wing formation. The overlapping of the scales is also developed to different degrees. I differentiate between three types: strongly, well and little covered (text fig. 47a, b, c); the transitions from one type to another are likewise a fluent transition.

Text fig. 47a-c.

Cover ratio of the scales.

a strongly covered, b well covered, c little covered.

There will certainly be a high occurrence of two types existing next to each other in one and the same ring and still, one will be able to decide on one type that appears to be characteristic for this ring. At times, one can find that neighbouring scales touch proximally as SLONAKER describes of *Passer domesticus;* common bittern, *Spizaëtus ornatus*, blackbird, carrion crow, magpie, yellow bunting and common bullfinch are further examples of this. I have examined a complete abutting of two scales on *Lophogyps* and hen harrier if one even wants to speak of the possibility of this phenomenon at all. I almost find it impossible that a so-called abutting exists at all, as the touching of these scales itself is already a slight overlapping, provided there is no interlocking.

**Quantity**

It will also be possible to establish an order of the vast range of scale counts. It has been known since GIEBEL that the scale count of birds varies between 10 and 17; according to my findings the maximum limit will be increased to 18, leaving us with a range of variation of 10 to 18 ¹. Assuming that the structural design of the ring follows some kind of rules, we have to expect regularity in regard to the variability of the scales. Regarding these rules I thought that a number is typical for a group of birds, defined either through their morphology or biology. If, hereafter, I have to discuss any numerical relations, then I will use the graphical representation as much as possible, as this tool presents the situation much more clearly than the mere figures themselves. First, we will look at the scale count in a ring in relation to the present number of rings as part of the total findings.

| Scale count in the ring ………………. | 10 | 11 | 12 | 13 | 14 | 15 | 16 | 17 | 18 |
| --- | --- | --- | --- | --- | --- | --- | --- | --- | --- |
| Number of ring examples: | | | | | | | | | |
| Total results ………………………………. | 1 | 18 | 26 | 57 | 138 | 182 | 31 | 3 | 4 |
| Results from other authors ……….. | 1 | 3 | 14 | 23 | 40 | 65 | 9 | 2 | - |
| Own results ………………………………. | - | 15 | 12 | 34 | 98 | 117 | 22 | 1 | 4 |

I calculate the percent frequency for the graphical representation.

| Scale count in the ring ………………. | 10 | 11 | 12 | 13 | 14 | 15 | 16 | 17 | 18 |
| --- | --- | --- | --- | --- | --- | --- | --- | --- | --- |
| Percent frequency of the ring examples: | | | | | | | | | |
| Total results ………………………………. | 0.22 | 3.91 | 5.22 | 12.4 | 30 | 39.4 | 6.73 | 0.65 | 0.87 |
| Results from other authors ……….. | 0.64 | 1.91 | 8.91 | 14.61 | 25.5 | 41.4 | 5.73 | 1.28 | - |
| Own results ………………………………. | - | 4.97 | 3.31 | 7.94 | 32.5 | 38.5 | 7.56 | 3.31 | 1.32 |

We are able to read a lot from the following image (text fig. 48).

Text fig. 48. The number of scales in a ring in relation to the number of ring examples.

Abscissa: Number of scales.

Ordinate: ring examples (as a percentage).

Overall findings: X X.

Findings of other authors: • •.

Own findings: ○ - · - · - · - ○.

(Likewise in the following graphical images).

________________________

1. The scale count of 30 from *Alca arctica* as per LEUCKART (cited page 538) has not been taken into consideration as this result is too far off from my own findings.

A scale count of 15 does not hold the absolute majority but is the most frequent; this is followed by 14 and finally, with a significantly smaller percentage 13, followed by 12 and 16. Thus, the statement from GIEBEL: “the usual number of scales is 13, 14 or 15” is verified and more defined based on more material. TILLY EDINGERS opinion that the sclerotic ring of birds consists “usually of 14 scales” is not correct. Incidentally, after all the findings, which are predominantly GIEBEL’s findings, the number 15 outweighs all others; the relation is here 25.5% to 41.4%. TILLY EDINGER probably comes to her conclusion based on GIEBELs findings. As to be expected, the extreme values in this mixture are present in less large numbers. From the number 10 the graph rises relatively steep and irregular, until it reaches the maximum value of 15 and then immediately plummets. I do not want to claim that what we can see here is a strongly shifted binomial graph. It almost appears that this is basically an increasing graph and the numbers 16, 17 and 18 are anomalous individual variation and they are not typical for the respective species. I will discuss this again further below.

I now find myself with the findings from three batches that are off differing origin. Due to the fact that the graph representing my own findings does not differ greatly from the one of the other authors, it seems fair to argue that they both in principle correspond with the true graph; this is now all the more expected from the graph resulting from both findings, alas the overall findings. It naturally meets our expectations that the percentage frequency of the three graphs differ.

I used all the data that was generally available in the previously discussed graphical image. It was available: ring pairs next to individual rings, species with many ring pairs next to those with often only one individual ring. This fact will surely have an impact on the overall finding. This is why, based on the presenting results, I have to determine the limit of the constancy of the scale count, to be able to make a definite statement as to whether a scale count can be typical for a species. It was not difficult to reach an affirmation regarding this. The number of deviations from this is certainly not small; looking at almost 29% of the available ring pairs that do not have the same number of scales in the right and left ring. The data so obtained is shown in the following table. Beforehand some explanatory comments. It was at times not easy to decide if a certain count of scales was typical for a species or only an individual case. These difficulties presented themselves in particular where only a limited number of rings were available, especially when each one of them had a different number of scales still. I most often decided on a scale count but only based on special moments that somehow indicated it. It was also difficult to consider rings as typical that were only available as one specimen. It was thus unavoidable that I included many species in the table with reservation, however, I have marked those with a *. The results from other authors have been marked with the initial of their names (A: ALBERS, E: EDINGER, G: GIEBEL, I: ISCHREYT, P: PARREIDT).

The typical number and formation of the scales of the different species.

| Name | Type | Number of scales | Formation | Author |
| --- | --- | --- | --- | --- |

After I have now sorted this wealth of findings as to whether they are a typical phenomenon or individual deviations, with as much of an accurate assessment as possible, we now want to examine these results, these typical findings, for the properties of the scale count to finally discuss the number of scales in particular. We are talking about 150 species that are spread as follows:

| Scale count in the ring ………………. | 11 | 12 | 13 | 14 | 15 | 16 | 17 |  |
| --- | --- | --- | --- | --- | --- | --- | --- | --- |
| Ring examples: | | | | | | | | |
| True number ………………………………. | 5 | 14 | 20 | 50 | 55 | 5 | 1 |  |
| Percentage ………………………..……….. | 3.34 | 9.33 | 13.35 | 33.33 | 36.6 | 3.34 | 0.67 |  |

Text fig. 49.

The number of scales in the ring in relation to the number of ring examples of the typical results (terminology as per text fig. 48).

The first question when looking at this graph (text fig. 49) must refer to the relation of this one to the previous one. This image gives us a picture of how the number of scales is distributed within the birds, whereas the previous data only shows how the number of scales is distributed within the available material. However, this must not lead to far-reaching differences. So, what does the new graph tell us?

The two extreme numbers 10 and 18 have disappeared, as they could only be interpreted as individual cases. Now we must state that we examined a scale count of 10 and 18, but that the average number of scales for the individual species varies only between 11 and 17. Furthermore, the difference in percentage distribution is important. Based on the first graph, the data under 11, 16 and 17 scales are too high, whereas 12, 13 and also 14 show figures that are too low. However, these differences are not of fundamental importance. It is only that this graphical image offers a stronger statement of what I had already particularly pointed out in the first one. It is absolutely possible, obvious even, that the frequency values regarding the number of scales will change when examining further species. That we would then see a completely different picture is already not to be expected based on the comparison of the four graphs, which are the results of various compositions. Based on my findings it is right to say that the frequency of rings increases with a growing number of scales to finally reach a maximum scale count of 15. I cannot reach another conclusion regarding the scale count of 16 and 17 other than that they are individual phenomena, thus abnormalities, or my statement above is not justifiable.

When assessing the variability of the number of scales within the class of birds, we were simultaneously able to examine that any number is typical for a species. It must now be our next task to find out the limits within this that is applicable, meaning how often and when does the number change from individual to individual or how often and when both rings exhibit differing numbers from each other. Assuming that a specific number of scales is typical for a species, we will have to ask to what extent a certain number of scales is typical for a group of birds, say an order. I chose such a far-reaching and comprehensive group, because only in an order is there a corresponding number of rings available to me for such general consideration. Nonetheless I was limited to 8 orders, due to the little available material.

I had already mentioned that 43 out of 148 pairs of rings, so at approximately 29% of all pairs of rings, showed a differing number of scales between the right and left ring. The difference in the scale count is in most cases only 1 scale, however, at times the difference can reach from two to three scales. *Chauna chavaria* with 12 and 15 scales, goose with 16 and 18, *Lophogyps occipitalis* with 15 and 17, Sparrow hawk with 14 and 16, tawny owl with 15 and 18, long-eared owl with 16 and 18 and lesser whitethroat with 13 and 16 are all examples of such large differences. That much regarding the deviation in the number of scales within an individual.

The deviation in the number of scales within a species can be in such a way that within the individuals there is consistency but the scale count varies from individual to individual, or there is also the possibility that the number varies within the individual and at the same time from individual to individual. As an example, for the first case scenario we can look at the domestic fowl and for the second case scenario the Eurasian coot. The variability in the domestic fowl is so big that one could be tempted to deny the presence of a typical number of scales. Out of 12 pairs of rings there are 2 pairs of rings with 15,15-5, pairs of rings with 14,14-1, pairs of rings with 13,13-2, pairs of rings with 14,15 and pair of rings each with 15,14 and 16, 15 scales; furthermore, the five individual rings are spread over the numbers 13, 14 and 15. Based on these findings I think it is justifiable to assume the number 14 as typical for the domestic fowl. Common buzzard and domestic pigeon are present in similar large number. In contrast to the domestic fowl, both species show a strong consistency in the number of scales; the common buzzard only shows one deviation out of the 19 rings as well as the domestic pigeon out of 10 rings. Both these examples, which I have chosen from several examples, are enough I think, to be able to maintain the claim of the relative consistency or the typical number of scales. It is only natural that the question regarding the cause of this noticeable variability in the domestic fowl arises. It stands to reason that the variability is a functional consequence of degeneration due to the domestication. As a supporting example, we can name the findings of the goose. The findings in the domestic pigeon admittedly contradict this opinion, or so it appears, but perhaps this is a consequence of the fact that the domestic pigeon is less domesticated, especially since the specimens provided by me were feral pigeons *(Feldflüchter)*. If the number of scales of an individual varies and then furthermore from individual to individual without the existence of a pair of rings in which both rings have the same number of scales, then it is hardly possible to determine the typical scale count. This is the case with the Eurasian coot. We have here available two pairs of rings; the one pair of rings has 12 and 13 scales and the other 13 and 14. Very similar is therein the closely related common moorhen that has pairs or rings with 12, 13 scales, 13, 14 scales, 14, 13 scales and finally 13, 13 scales. Only the two pairs of rings with 13, 13 scales are giving us clarification regarding the typical number of scales. It is safe to assume that further examinations of the Eurasian coot will also show pairs of rings with 13, 13 scales.

After I have now presented the relative consistency of the number of scales and especially their deviations in ample detail, I would now like to look at the scale count within certain groups. I will now, based on the scale count, verify the assumption that based on the degree of relationship there is a correlating consistency in the entire structural design of the scales. To have as many results as possible, I will again use all available findings (right and left rings, typical and non-typical phenomena) and not only the ones ascertained as typical. As we were able to see from the previous graphs, hardly anything on the graph changes. On the contrary is to expect that the non-typical cases in the following graphs will have a worsening effect.

I begin with the order Anseriformes (text fig. 50):

| Number of scales ……….………………..…. | 12 | 13 | 14 | 15 | 16 | 17 | 18 |
| --- | --- | --- | --- | --- | --- | --- | --- |
| Number of rings ……………………………... | 1 | 1 | 2 | 31 | 7 | - | 2 |
| Percentage ………………………..……….….. | 2.27 | 2.27 | 4.55 | 70.3 | 15.9 | - | 4.55 |

The number 15 is quite obviously the typical count for this order. If we compare this figure with our table, then the picture changes in so far as 12 and 18 disappear and 16 is reduced by a count of five and 14 by a count of one. This means that this order almost exclusively has 15 scales.

Text fig. 50. The number of scales in Text fig. 51. The number of Text fig. 52. The number of

the ring in relation to the number of scales in the ring in relation to scales in the ring in relation

rings of the Anseriformes (legend see the number of rings of the birds to the number of rings of

text fig. 48). of prey (legend see text fig. 48). Galliformes (see text fig.48).

Birds of prey (text fig. 51):

| Number of scales ……….………………..…. | 13 | 14 | 15 | 16 | 17 |
| --- | --- | --- | --- | --- | --- |
| Number of rings: | | | | | |
| Total results ……………………….…………... | 2 | 10 | 52 | 7 | 1 |
| Results from other authors .……….….. | - | 7 | 18 | 2 | - |
| Own results …………………………………….. | 2 | 3 | 34 | 5 | 1 |
| Percentage: | | | | | |
| Total results ……………………….…………... | 2.78 | 13.9 | 72.2 | 9.72 | 1.39 |
| Results from other authors .……….….. | - | 25.95 | 66.8 | 7.4 | - |
| Own results …………………………………….. | 4.44 | 6.65 | 75.5 | 11.1 | 2.22 |

Also, in this order the number 15 is typical. According to our table, the number 15 would account for a much higher percentage, as 17 disappears completely, 16 reduces by a count of 6, 14 reduces by a count of 4 and 13 reduces by a count of 1.

Galliformes (text fig. 52):

| Number of scales ……….………………..…. | 11 | 12 | 13 | 14 | 15 | 16 |
| --- | --- | --- | --- | --- | --- | --- |
| Number of rings: | | | | | | |
| Total results ……………………….…………... | 1 | 2 | 4 | 32 | 13 | 1 |
| Results from other authors .……….….. | 1 | - | - | 8 | 1 | - |
| Own results …………………………………….. | - | 2 | 4 | 24 | 12 | 1 |
| Percentage: | | | | | | |
| Total results ……………………….…………... | 1.89 | 3.78 | 7.53 | 60.3 | 24.5 | 1.89 |
| Results from other authors .……….….. | 10.0 | - | - | 80.0 | 10.0 | - |
| Own results …………………………………….. | - | 4.65 | 9.30 | 55.95 | 27.28 | 2.32 |

Based on the table values, this graph would look vastly different. The numbers 11, 13 and 16 disappear completely and it would then clearly say that 14 is the typical number, which can also be seen in the above graph; the number, however, would still stand out more clearly. And those 12 scales from *Opisthocomus* seem to be an important factor regarding the family relations; more about this at a later stage.

Gruiformes (text fig. 53):

| Number of scales ……….………………..…. | 12 | 13 | 14 | 15 |  |
| --- | --- | --- | --- | --- | --- |
| Number of rings: | | | | | |
| Total results ……………………….…………... | 2 | 14 | 7 | 6 |  |
| Own results …………………………………….. | 1 | 11 | 7 | 2 |  |
| Percentage: | | | | | |
| Total results ……………………….…………... | 6.9 | 48.4 | 24.2 | 20.7 |  |
| Own results …………………………………….. | 4.76 | 52.4 | 33.4 | 9.52 |  |

13 is the typical number, whereas 14 and 15 stand close beside it with a relatively high percentage.

Charadriiformes (text fig. 54):

| Number of scales ……….………………..…. | 10 | 11 | 12 | 13 | 14 | 15 | 16 | 17 |
| --- | --- | --- | --- | --- | --- | --- | --- | --- |
| Number of rings: | | | | | | | | |
| Total results ……………………….…………... | 1 | 16 | 2 | 9 | 3 | 33 | 3 | 1 |
| Results from other authors .……….….. | 1 | 2 | - | 9 | 1 | 17 | 1 | 1 |
| Own results …………………………………….. | - | 14 | 2 | - | 2 | 16 | 2 | - |
| Percentage: | | | | | | | | |
| Total results ……………………….…………... | 1.48 | 23.6 | 2.95 | 13.28 | 4.43 | 48.6 | 4.43 | 1.48 |
| Results from other authors .……….….. | 3.12 | 6.24 | - | 28.2 | 3.12 | 53.1 | 3.12 | 3.12 |
| Own results …………………………………….. | - | 39.0 | 5.55 | - | 5.55 | 44.5 | 5.55 | - |

Text fig. 53. The number of scales in the Text fig. 54. The number of scales in the ring in

ring in relation to the number of rings of relation to the number of rings of

the Gruiformes (legend see text fig. 48). Charadriiformes (legend see text fig. 48).

We can see a lot from this graphical representation. In front of us is a graph with three peaks – on 11, 13 and 15 – as those are maxima, which means that for this order all three numbers are characteristic. From the table we are also able to see from which individual representatives those numbers originate. *Columbidae* have 11 scales, *Alcidae* have 13 scales and *Laridae* as well as *Charadriidae* have 15 scales.

Cuculiformes (text fig. 55):

| Number of scales ……….………………..…. | 11 | 12 | 13 | 14 |
| --- | --- | --- | --- | --- |
| Number of rings ……………………………... | 1 | 15 | 1 | 2 |
| Percentage ………………………..……….….. | 5.25 | 79.0 | 5.25 | 10.5 |

According to our table, this graph too would be significantly steeper as 11 would disappear completely and 14 would be reduced one count; the number 12 reaches an already high percentage in any case so that it can count as one of the most conclusive graphs.

Coraciiformes (text fig. 56):

| Number of scales ……….………………..…. | 12 | 13 | 14 | 15 | 16 | 17 | 18 |
| --- | --- | --- | --- | --- | --- | --- | --- |
| Number of rings: | | | | | | | |
| Total results ……………………….…………... | 2 | 16 | 6 | 33 | 12 | 1 | 2 |
| Results from other authors .……….….. | 1 | 7 | 2 | 6 | 2 | 1 | - |
| Own results …………………………………….. | 1 | 9 | 4 | 27 | 10 | - | 2 |
| Percentage: | | | | | | | |
| Total results ……………………….…………... | 2.78 | 22.25 | 8.35 | 46.0 | 16.6 | 1.39 | 2.78 |
| Results from other authors .……….….. | 5.25 | 36.75 | 10.5 | 31.5 | 10.5 | 5.25 | - |
| Own results …………………………………….. | 1.89 | 17.0 | 7.53 | 51.0 | 18.8 | - | 3.78 |

This graph reminds us of the graph of Charadriiformes due to its numerous peaks. However, it differs because it only has two peaks. Both these peaks show however that the numbers 13 and 15 are characteristic for this order. In this order too, both numbers are distributed analogously. Rollers, hoopoes, kingfishers and woodpeckers have 13 scales, whereas owls, nightjars and Cypselidae have 15 scales.

Text fig. 55. The number of scales in the Text fig. 56. The number of scales in the

ring in relation to the number of rings of ring in relation to the number of rings of

Cuculiformes (legend see text fig. 48). Coraciiformes (legend see text fig. 48).

Passeriformes (text fig. 57):

| Number of scales ……….………………..…. | 13 | 14 | 15 | 16 | |
| --- | --- | --- | --- | --- | --- |
| Number of rings: | | | | |  |
| Total results ……………………….…………... | 9 | 65 | 4 | 1 | |
| Results from other authors .……….….. | 2 | 12 | 2 | - | |
| Own results …………………………………….. | 7 | 53 | 2 | 1 | |
| Percentage: | | | | |  |
| Total results ……………………….…………... | 11.4 | 82.3 | 5.05 | 1.26 | |
| Results from other authors .……….….. | 12.5 | 75.0 | 12.5 | - | |
| Own results …………………………………….. | 11.5 | 84.2 | 3.17 | 1.59 | |

This graph is the steepest one and it would almost be conclusive if we used the figures from the table. Because there we can only see the number 14 beside a 13 and 15.

The graphs that we just discussed give us a very clear picture regarding the distribution of the scale count over the individual bird groups. Especially noteworthy are the Coraciiformes and the Charadriiformes, for which not only one specific scale count is typical, but numerous ones. I will specifically revisit the meaning of this fact again at a later stage. The only number of scales that appear are from 11 to 15; they are distributed as follows: birds of prey, Anseriformes, a part of the Coraciiformes and a part of the Charadriiformes have 15 scales; Passeriformes and Galliformes have 14 scales; Gruiformes, a part of the Charadriiformes and a part of the Coraciiformes have 13 scales; Cuculiformes have 12 scales and a part of the Charadriiformes have 11 scales. According to these results it is fair to argue that each order exhibits a specific number of scales. Also due to this, the statement regarding the consistency of the scale count is being supported from another side. Due to the fact that the number 14 is by far the most common number for the order of Passeriformes, we have to take a closer look at an initially made statement. Based on the present findings, I dared to state that the number 15 has the majority. Should Passeriformes almost continuously show 14 scales, which is probable, then the number 14 obviously has the majority, because three fifths of all bird species belong to the order of

Text fig. 57. The number of Passeriformes and T. EDINGER and A. DABELOW would then in this

scales in the ring in relation sense be right. But this is not what both authors meant and the

to the number of rings of evidence seem to indicate that a scale count of 14 is not an exclusive

Passeriformes (legend see average for all orders.

text fig. 48).

1. **The ring**

**Shape**

The sclerotic ring already shows great diversity in its design, even when only looking at it briefly. If the scales of a ring are arranged in such a way that they are positioned almost in the basic level of the ring, then we speak of a flat ring. If the scales are raised and if their inclination grows to a maximum, then the ring goes through all possible shapes up to the other extreme, the cone or tube shape. In between both of these extreme shapes, all other shapes occur, more or less. For the sake of simplicity, I assign all rings to four basic shapes according to the relation of their scales to the ring base: flat, ascending, steep, tubular (text fig. 58 a-d).

Text fig. 58 a-d.

Basic shapes of the sclerotic ring.

a) flat

b) ascending

c) steep

d) tubular shaped.

The classification can only be made through visual inspection, whereby in my opinion the necessary accuracy remains protected. Some rings are shaped in such a characteristic way that it is immediately clear which shape they belong to (eagle owl, tawny owl, common bussard). The frequency of the four basic shapes reduces with steepening. Steep rings or even the tube shape are rare phenomena, whereas flat rings can be found very often. Typical representatives of a flat ring are Passeriformes, apart from a few exceptions. Penguins, *Sula* and certain birds of prey have a steep ring. It is well known that the ring of owls is tube shaped and does not require any further statement. Less well known up to now is the similar steepness of the ring in *Cypselus*, which is however highlighted by ROCHON-DUVIGNEAUD and which I can verify.

**Structural design**

The outer shape is not the only distinctive feature of the rings, but the most prominent. More important are the differences of the structural design. The complete proceeding discussion would be unnecessary if the rings had a mere roof tile shaped kind of coverage and did not also feature scales that cover in a particular way, that are represented in twos or fours, sometimes even in sixes.

Involuntarily the next question that arises is why does the coverage all around in a ring not occur without interruption. It is not our first task to answer this question but to analyse the kind of coverage to be able to determine any possibly existing rules: do we even know if the question based on this causal relationship implies the opportunity for a solution? Perhaps as a result the reason will become known as to how the sclerotic ring developed this structural design with its ausgezeichnete scales and not the other ones with the simple kind of coverage. Due to the fact that rings have ausgezeichnete scales and through the variation in their arrangement within the ring, each ring has a specific look.

The first and most obvious difference in structural design is if a ring has one or two pairs of ausgezeichnete scales. Thus, how is the A- and B-type distributed? I can summarize my examinations regarding this in two sentences:

1. The A-type is the more common one and can be found in every order.

2. The B-type is limited to the orders birds of prey and Coraciiformes.

With regards to the Coraciiformes, the existence is further limited to the owls, whereas such a distinction within a specific group cannot be found within birds of prey. Based on my findings, I initially believed that birds of prey were consistently built according to B-type, until an array of examples showing the A-type proved the opposite. Without those examples, I would not have felt confident about the accuracy of GIEBEL’s results, of which almost half exhibit A-type. I was especially astounded by the fact that the honey buzzard was supposed to be A-type compared to the closely related common buzzard with B-type. That this is a typical and not an individual anomaly can be presumed after confirmation of GIEBEL’s results, albeit ALBERS speaking of the B-type. How the B-type of the skylark is to be interpreted is hard to decide. It is, however, surprising that the skylark is the only bird out of the many representatives of the Passeriformes that is supposed to have B-type. But the fact that the right and left ring do not differ from each other, neither in number nor layout of scales, points very much towards this being a typical anomaly.

The parrot species Jako will be similar. ALBERS states the observation of B-type, whereas all Ouculiformes show A-type; I do not believe that ALBERS made a mistake. Already the significant difference in scale count, which GIEBEL verifies by the way, can make the validity likely. I can, however, not prove it. Another example for a typical anomaly is the barn owl. The first pair of rings that I examined presented A-type. I was hoping a second pair of rings was going to verify this statement, whereby I received the answer yes and no, i.e. the right ring was built according to A-type and the left one according to B-type. Therefore, the barn owl is at the same time an example for an individual anomaly. That the A-type is characteristic for the barn owl and not the B-type can be assumed; or do we have a species here that can easily change its type?

Clearer is the case of the long-eared owl, which exhibits the A-type according to GIEBEL. If this observation is not based on an error, then this is surely an individual case, as I found without exception only B-type in all of the 17 rings that I examined. In this regard, the deviations of the domestic fowl are remarkably interesting. Out of 29 rings we find 3 rings with B-type, which are distributed over one pair of rings and an individual ring. There is not the slightest doubt that these are individual deviations. This occurrence too, just as the notable variation in scale count, could be interpreted as a result of domestication. If the number of scales and the type of a ring are not purposeless occurrences, then it is reasonable to expect a similar conclusion regarding the formation of the scales.

I already mentioned that the plus-scales are positioned dorsally and ventrally and that the minus-scales are positioned nasally and temporally. What is furthermore striking, is that the minus-scale is not positioned in the ventral half of the ring, and that the ventral plus-scale and the temporal minus-scale are present in the B-type. I do not know of any deviations of this last fact, whereas few and insignificant deviations exist from the previous ones. Now, it is not the case that the position of both of the plus-scales is defined with mathematical accuracy, that they, for example, are both positioned on the diameter of the ring, but small deviations from this can be examined everywhere. Examples of notable deviations would be: barn owl, the left ring of the unknown seagull and finally the single ring of the domestic goose. I examined the dorsal plus-scale regarding its relation to the head on numerous birds for a more precise assessment of the positioning of the plus-scales in the ring. I thereby found that this scale was always positioned in the same place or only differed so slightly that, based on my method of examination, it did not appear as such. This spot, that thus recurs with quite some regularity, at least insofar as I undertook examinations, is positioned on the lot that is found in the centre of the pupil on the straight line between nostril and the centre of the pupil (text fig. 59).

Text fig. 59.

For information on the positioning of the plus-scale.

The arrow shows the position of the nictitating membrane tendon.

Then, we have to say that the other plus-scale is located more ventral-nasally or more ventral- temporal, depending on species. So if I start my counting with the lower lying scale, apart from few exceptions, then it does not mean that we are talking about a scale that is absolute precisely positioned ventrally. Firstly, a definition regarding the positioning of the scales cannot be made so explicitly and secondly, I have only been able to make out that orientation on a few specimens; I usually went by the position of the nictitating membrane, in particular the nictitating membrane tendon, and that is how I always found the upper plus-scale in an exact dorsal position. The nictitating membrane inserts in the area of the dorsal plus-scale and the nictitating membrane tendon runs from the ventral plus-scale slightly temporally apart, around the bulb. On a ring of an eagle owl, I was able to determine that the nictitating membrane tendon runs alongside the plus-scale. When we speak of the formation of the ausgezeichneten scales and we know the total number of scales, then we plainly know the formation of the scales.

Since under the B-type only two scales exist, which can change their position to each other, the possible combinations are significantly smaller than under the A-type with four variable scales. I thus only know the following possibilities of distribution of the ausgezeichneten scales under B-type: 1, 5; 1, 6; 1, 7 and 1, 8. One can justifiably speak about typical formation of the scales for the species. The deviations correspond with those of the scale count; I inserted what was, in my opinion, the typical formation in the general table. I do not find it necessary to repeat all the values that exist under A-type again at this point, but I will point out the most frequent ones; 1, 9; 5, 11 – 1, 9; 6, 10 – 1, 8; 5, 10 – 1, 8; 6, 9.

In response to the question as to whether a certain formation is characteristic for a scale count, the answer has to be without a doubt, yes. The scale count of 15 is almost throughout connected to the formation 1, 9; 5, 11. As examples for this I can name: Anseriformes, dowitcher, Laridae – but of course not exclusively - furthermore the limpkin *Rhinochetus jubatus,* and the European nightjar. For a scale count of 14 the following is first and foremost possible: 1, 8; 5, 10; the same applies to a scale count of 13; for a scale count of 12 it would be 1, 7; 4, 9 and finally for 11 scales it would be 1, 7; 4, 8. Unfortunately I cannot draw any conclusions regarding the reasons for these deviations from the present results. What is certain, is that a certain formation is more or less characteristic for the individual number of scales.

The circumstance of interlocking of the scales cannot so easily be dismissed by saying that it somehow interrupts the regular way of covering of the scales. Upon closer inspection we ascertain remarkable characteristics here as well. Particularly interesting for that is the series of common buzzards. Out of 18 rings 14 have interlockings, whereof the same scales are involved 12 times. It is at least very striking that it always involves scales number 6 and 7, and that in four pairs of rings exactly the same interlocking was examined in the right and left ring. I can prove the fact that the same interlockings become more frequent within a species, well even within an individual, with further material.

*Spizaëtus* has an interlocking in the right and left eye between 1 and 2, 5 and 6 as well as 9 and 10; a goshawk also has the same interlocking in the right and left ring. Further examples for this are Papagei and a fieldfare with 5 X 6 right and left, a tawny owl with 1 X 2, an eagle owl with 1 X 2 and finally a mountain finch with 1 X 14. Very striking is that the interlockings are the same kind in the right and left ring, excluding few exceptions; one can even speak of a mirrored likeness. Further to mention is that the interlocking 7 X 8 occurs relatively often. It is also very noticeable that most of the interlockings are positioned temporally (approximately two thirds of all interlockings).

**5. Sclerotic ring and genealogical tree**

The variability in the number of scales that make up the ring is limited characteristically within certain groups of birds. This fact, which has been sufficiently proven from the prior material, leads us to the question to what extent there is a connection between the scale count and relation. Here again, I can only make assertions that are within the borders of reliability of the current findings. Firstly, I would like to determine to what extent the meaning of the scale count can be deduced from the genealogical tree, and secondly I am interested in the question, which is however less important, if the present findings offer possibilities for clarification of controversial questions of relation.

When briefly looking at the genealogical tree in the “Geschichte der Organismen” by FRANZ, we immediately notice that almost all groups with smaller numbers group together and are located on a lower level. Not much more can be seen at a first glance. Only a closer comparison of related groups shows further connections. PYKRAFT’s genealogical tree shows basically the same. What seems considerable to me is that *Opisthocomus* has 12 scales, whereas Galliformes, which according to the opinion of many is a close relative, are representatives for a scale count of 14. I unfortunately only had access to one pair of eyes, which however had 12 scales in the right as well as the left ring. Based on all my findings, I can however not assume that *Opisthocomus* is, to such a great extent an individual case, distant from the Galliformes. The following observation is based on the condition that typical deviation of *Opisthocomus* has been proven, which according to me is very likely. Even so, I would like to emphasize again that my argument has only a conditional value.

The systematic position of *Opisthocomus* is highly controversial as it is a strange bird based on its structure and lifestyle. Some researchers put it as belonging to the Musophaga (Pisangfresser), others however determine it to be close to the rails; then again it is thought to be distantly related to the cuckoo birds (literature in FRANZ, Geschichte der Organismen). According to HUXLEY it is closer to the Galliformes and Columbidae than any others. According to BREHM it is the most closely related to gallinaceous birds and is thus listed as a suborder under Galliformes. BÖKER however explains this to be untenable and lists it as a suborder under Cuculiformes. Due to the fact that the Cuculiformes that I examined showed 12 scales, whereas the gallinaceous birds showed 14, I think it justifiable to declare *Opisthocomus,* from the point of view of the sclerotic ring, to be closer related to the cuckoo birds rather than the gallinaceous birds. I cannot decide as yet what meaning this argument or the argument of the scale count in general holds.

Doves exhibit a very different scale count compared to the group that stands together with them in an order. According to BREHM, the suborder of the Columbidae is “one that is probably self-contained”, which in my opinion is being emphasized based on the scale count. There are also various opinions regarding the position of doves in the genealogical tree. GADOW wants to derive them from dowitchers, whereas FRANZ makes a case for relatedness with gallinaceous birds. Interesting are CHANDLERs findings regarding this, who records the phylogeny of the birds based on the morphology of the feathers. CHANDLER derives them from the connection *Galliformes-Cuculiformes*, in fact very closely to the Galliformes. Based on this, doves are at least near the Cuculiformes, which I find likely based on my findings. Also, the consistently small-scale count of doves and *Opisthocomus* points perhaps towards the just mentioned relation.

These are not the only examples of groups, that differ from other groups in their order based on their prominent scale count. Further examples are *Alcidae*, rollers and woodpeckers. Alcidae with their 13 scales differ just as thoroughly from the rest of the Charadriiformes as doves do with their 11 scales. If they differ like that from their closest relatives, the seagulls, then one could perhaps explain this through them having acquired a number of convergences with diving birds in their external appearances and way of life. That rollers and woodpeckers differ that strongly in the structure of their sclerotic rings from other Coraciiformes (owls, Cypselidae), must not surprise us as then again we are dealing with a substantial order. Phylogenetically, FRANZ positions rollers as less specialized types in the middle between Cuculiformes and passerine birds, not far away from the main stem. The suborder of rollers consists of five closely related, relatively basic built families and forms the basis for the order of the Coraciiformes. Woodpeckers are to be looked for close by, whereas owls, Cypselidae and nightjars have distanced themselves due to specialisation. According to BREHM, the phylogenetic origin of woodpeckers is to be looked for within the sphere of rollers, probably around the kingfishers and motmots. According to the scale count too, these two main groups can be separated – owls, Cypselidae on the one hand and rollers, woodpeckers on the other hand.

Especially noteworthy, particularly at this point, is the similarity of the sclerotic ring between swift and owls. Not only the scale count, but rather the ring shape puts these two groups together, which do without a doubt belong together based on their way of life. Perhaps though, the scale count of owls points towards kinship relations with birds of prey (diurnal birds of prey), as v. BOETTICHER assumes also in other respects a closer relationship between owls and diurnal birds of prey than the currently existing GADOWSCHE view on this matter.

Finally, Gruiformes also belong here as an example. This too is a very diverse order; appearance as well as way of life differ within the various species. According to BREHM, the family of rails, phylogenetically seen as an old family, constitutes the middle, around which the other families of the order group. If we compare this fact with our scale count, then we will recognize a corresponding difference here too; rails have the least number of scales, whereas other families with a higher scale count stand alongside them.

The results are quite obvious. We were able to examine that shapes, which exhibit fundamental differences within their species**,** also differ in their scale count. What is furthermore important is that a lower scale count is typical for phylogenetically old shapes. Our findings show a number of things, but they only suffice to answer such comprehensive and general questions with probability.

What needs to be heeded too, is that a relatively high degree of consistency is being reached especially of the passerine birds as a highly developed and species-rich branch at the end of the genealogical tree**.** We often find similar relationships in the animal kingdom; one should be reminded of mostly constant number of segments in *Malacostraca* or the mostly constant number of cervical vertebras in mammals.

**6. Histogenesis of the sclerotic ring**

The morphological circumstances do not yet give us a final indication regarding the nature of the sclerotic ring. Some important questions remain problems for now. This is firstly the question why do the ausgezeichneten scales exist at all and secondly, how do certain formations come about? To further clarify this, I feel the revising of the embryonic development is crucial. To what extent any knowledge in this area gets us closer to a satisfying solution is hard to tell; but the embryonic development on its own is worth clarification.

It was first and foremost the phenomena of interlocking that would not allow me to relinquish the thought that the scales have certain origin centres from where they form through appositions within the connective tissue. Only due to this did I believe that the interlockings could be explained as random occurrences, a consequence of localized tissue damage.

My task can be summarized into two questions, whereby the last one is still to be broken down into individual questions.

1. When does the sclerotic ring form?

2. In what manner does this formation happen?

Question 1 is easily understandable, but question 2 needs to be refined through the following formulations:

a) Is the sclerotic ring being formed as a homogenous ring and as a result of this are the scales

a secondary occurrence?

b) If a) is out of the question, then does each scale start to form in its full size straight away?

c) Does the formative tissue already show any special characteristics that point

towards the kind of positioning of the scales?

To begin with, a few general comments regarding the available bibliographical references in the question of the histogenesis. Information to this very specific problem is in general as sparse as it is in the problem of the sclerotic ring. What does exist about it in the literature, has usually been found in passing. However, HEINRICH MÜLLERS earned special mention of his observation that the bone develops directly out of a soft blastema. KESSLER, who worked on the development of the eye of chickens, cannot make any certain comments regarding the timing of the formation of the ring. LEPLAT, however, already offers one positive answer. If he had not dismissed the sclerotic ring to be of marginal importance compared to the main issue in front of him, then he would probably have been able to give correct information regarding the development of the scales based on his median section.

In this median section through the anterior part of the bulb of a 12-day old chicken embryo, we can already see the formation of the bony ring; here one scale can be seen, that is however only situated in the proximal area of the scleral section between cornea and cartilaginous cup and does not extend over the entire section. He simply limits himself to state the fact that: *“… l’anneau osseux, encore étroit, est déjà formé, assez périphérique relativement à ce qu’il sera l’adulte” )“The bone ring, still narrow, is already formed, placed relatively peripherally compared to what it will be in adults.”).* We will find out from my examination what this section is about.

That the ring is already inherent in a 12-day old chicken embryo is the only positive fact that LEPLAT can state. This exhausts the knowledge of the ontogenesis of the sclerotic ring.

I continue my research on the assumption of LEPLATS findings and believe that it is safe to assume that the sclerotic ring is not laid out the way we know it in its adult state. That is also the only thing that shows in this median section. I thought the 12-day state to be suitable to give us clarification regarding the forming of the sclerotic ring.

I was disappointed from the first series of sections as I was not able to find even the slightest trace of a bone scale. The second series of sections, which was also from a 12-day old chicken embryo, just like the first one was, shows the sclerotic ring at a stage of development that gives important information regarding the histogenesis. I took meridional cuts, so that some of the scales are seen lengthwise and others crossways. When reconstructing this series of sections, we found a completely different picture of the sclerotic ring than we know it in its adult stage. The scales do not overlap on their edges but leave a space between them. They also do not reach as far after the cornea and the sclera cartilage as we otherwise know it. Based on this, LEPLAT’s section can be easily understood.

Depending if the cut in the interstitial spaces runs parallel or diagonal to the edges, it will either intersect a scale or only a corner of one. This corner can then again be positioned proximally or distally in the sclera, also both options are possible at the same time, so that there can be a bone free space between both parts. The first case is now what we saw under LEPLAT.

As a result of the preservation of the embryos available to me, it was unfortunately not possible with this material to obtain an overview image than is better than sufficient. What is however important is to prove that at a particular embryonic stage, the scales are not yet developed enough that they overlap each other. The picture (text fig. 60) seems to show this clearly enough.

Text fig. 60 meridional cut through the anterior part of the eye of a 12-day chicken embryo.

*Ep.* Epidermis, *Bdg.* Connective tissue, *Bl.* Blastema, *Kn.* Bone, *S.* sinewy connective tissue, *Abl.* Epithelium pigmentosum, *Ibl.* Epithelium nonpigmentosum of the Pars ciliaris retinae.

If in the adult stage, both neighbouring scales overlap each other respectively and if there is a condition at a certain embryonic stage as we have just shown, then it might be assumed that the two neighbouring scales – I would like to use a mental image – gradually slide over each other. It is then right to assume, and does not need to be verified first, that each scale has a centre of origin from which it grows to all sides. The scales are surrounded from both sides, from the inside as well as the outside, by connective tissue and they grow in a layer which is characterized by a particular richness in kernels. – H. MÜLLERS “Blastem” (text fig. 60, *Bl.*). I would like to specifically point out that the one scale is positioned on the outer side of the layer, which is rich in kernels and that the other one is positioned on the inner side. The kernels are, however, only to be found at the end of the scales. I cannot see a reason as to why the scales spread apart in such a way at this point and do not grow towards each other to eventually fuse. I do not know to decide if this wavy behaviour of the sinewy layer of connective tissue is in any kind connected with the offsetting. The strong curvature of the one scale does not have a principle meaning and is only to be observed on this scale; perhaps this is a post-mortem compression phenomenon. Otherwise this picture shows everything that can be seen at the other parts of this series of sections, not more and not less.

I think with this, I will be able to answer the question posed at the beginning. With regards to the timing of the formation, it is certain that it is around the 12^th^ day. When we were able to examine that nothing could be seen in the first series of sections, although this was also of a 12-day old embryo, whereas in the other object so much existed already as we could see above, then we could conclude that the scales develop relatively quickly. We would answer question 2 that, based on this series of sections it is right to assume that a so-called centre of origin exists for each scale. This means that interlockings can in my opinion be understood correctly. In my opinion, they form in such a way that the two scales cannot grow towards each other on two parallel levels due to some kind of tissue damage, but that they have to grow towards each other on two diagonally inclined levels (or similar), which then results in the “crossing” (interlocking). With regards to the question as to whether there were any specific characteristics in the formative tissue just before the formation, which relates to the kind and positioning of the scales, I was unfortunately unable to determine anything.

**7. The function of the sclerotic ring**

The findings that exist regarding this all lack the necessary validity; and I do not want to increase the many assumptions by adding more to them. It is my task to verify if we can reach more positive results based on the present findings. I start my observation with the fact that the sclerotic ring in birds is a structure, which has unmistakeable regularities in its entire formation. Surely it is not randomness when the scale count despite variability within certain groups shows something typical; and when it is small in this group but big in that one; when this group exhibits A-type while the other one shows B-type; and finally when these birds exhibit this formation of ausgezeichnete scales, whilst those birds show another one. The assumption that specific functions require the respective design of the ring cannot be denied. The great diversity of build and way of life of birds runs parallel to the diversity of the ring structure.

Firstly, a compilation of the existing assumptions regarding the functional meaning of the ring:

1. Support and protective function.

2. Extension of the eye socket.

3. Protective function against exposure to light due to screening effect.

4. Segmentation of the ring for particular reinforcement.

5. Scales as solid points of attachment for the muscles.

6. Adaptation to accommodation.

The fact that the sclerotic ring has a supporting function is so obvious that it does not require a big discussion relating to it. Two factors are of importance when forming the ring. According to the way of life, what is essential:

1. relatively big brain, 2. Relatively little cranial matter.

The implementation of this principle would only allow for an eye that could not comply with the demands of bird life. Therefore, because the bird’s eye only has a relatively flat eye socket available, in which the eye must be particularly big according to its performance, a replacement is to be obtained for the virtually missing part of the eye socket. ALBERS already regards the sclerotic ring as an extension of the wall of the eye socket. Due to his opinion that a big part of the bony ring is being covered by brow bones and the lacrimal bone, he thinks that “… thus also the anterior upper pieces of the bony ring are shorter than the posterior ones”. He continues: “This shows that we can actually only regard the part of the bony ring that is situated at the small corners of the eye as an extension of the eye socket”.

That the upper nasal scales are smaller than the temporal scales is almost accurate. All things considered, all nasally located scales are smaller than the others; there are also cases where the nasal-ventral scales are the smallest. This asymmetry of the ring signifies an asymmetry of the bulb. If we suppose that the ring is indeed an extension of the eye socket, then we should rightly consider if the brow bones and the lacrimal bones are not the initiator of the asymmetry of the ring and therefore also of the entire bulb. The eye in birds is generally more positioned towards the front than that in reptiles and touches due to this positioning, phyletically speaking, the orbital bone, which is why it shrinks on the nasal side. The asymmetry is strongest on big eyes, as they are long-axis eyes and therefore require the most convergence towards the front.

A change in the relative size of the bulb must entail a corresponding shift in steepness and size of the sclerotic ring: relatively small eyes have an inconspicuous ring, relatively big eyes on the other hand have an unexpectedly big ring. That the shape of the ring is so variable, need not surprise us when we look at PLATE and some of the figures for birds that indicate the relation of weight of both eyes to the entire body weight. Some examples named there: gray goose 1 : 567, peacock 1 : 326, magpie 1 : 72, kestrel 1 : 35, tawny owl 1 : 32.

This order correlates to the one in rings, judged on a visual estimate. To get exact figures, one would have to numerically ascertain the ratio between eye and sclerotic ring.

Very little is to be said about the screening effect of the sclerotic ring. Together with T. EDINGER, I am of the opinion that we do not need to consider a possible function as a screen. Experimental evidence for this has been provided on the eyes of *Ophisaurus*. When looking at the sclerotic ring of birds, the entire structure speaks, in my opinion, already from the start against such an assumption (the possession of ausgezeichnete scales; the often very insignificant slidability of the scales due to the strong layer of connective tissue embedded in them, which is particularly noticeable at the distal part of the ring. After having examined preserved material, I only think the slidability of scales possible under certain conditions, which in my opinion are not met in a functioning eye. Thus, I cannot agree with SLONAKER, who associates the slidability of scales with the accommodation.

The question regarding segmentation of the ring also seems very important to me. There are a number of factors that I find noteworthy, which should be considered when resolving this question. The notion of FRANZ, “that the fragmentation of the ring into scales cannot fulfil any other purpose than one of strengthening”, can only be accurate for rings with hollow scales, which most are not. Regarding scales that are not hollow, it would be more plausible that a ring which is made of such pieces, is more elastic than a uniform ring. I think to see the interlockings as a specialization for the supporting function is even less correct. My results clearly show that the interlockings are individual disruptions.

The explanation of the segmentation as a means to elasticity would not be sufficient, as we would rightly have to ask for the necessity of this elasticity; the fluctuating internal pressure is however no explanation for it.

In my opinion the segmentation is plainly linked to growth. We saw that the ring is already laid out in its true shape early on. Because the growth of the eye is at that point not yet concluded, the ring must be designed in such a way that it can comfortably follow the changes in proportion to its surroundings. And for that, the segmented ring seems to be in my opinion the best and perhaps the only possible solution. I would like to compare this principle of construction with the one of the bony skull, which is also made up of many separate bones. The task to show a capsule, which has to already be functional accordingly in the juvenile stage is applicable to both. The skull, as well as the sclerotic ring, have bony plates that are unobstructed in their growth and that, on their own accord, do not hinder the growth of the included organs; they also afford the necessary stability due to their positioning towards each other. The sclerotic ring generally shows the overlapping of the plates, and only sometimes can we find reminders of the interlocking with jagged edges as we know it from skulls.

The degree of segmentation as well as the scale count cannot be functionally interpreted at this time. I tend to understand the scale count as an organisational characteristic, as a characteristic which is to be understood as phylogenetically. Such a characteristic is the result of driving forces from within, which I find highly probable here. I at least cannot see that the scale count could be a feature of adaptation. Perhaps our case can be very well compared to a botanical fact. The pentamery or tetramery of the perianth is most likely only determined by inner factors. This fact, transferred to our case, would mean that the number of scales is generally seen as an organisational characteristic.

In the proceeding text, I would like to try to give an interpretation to the kind of segmentation, the presence of ausgezeichneten scales. Due to the fact that from the outset, the scales are not laid out the way that they appear at the adult stage, but only slowly reach their normal size due to growth along the edges and finally slide over each other in a sense, we have to ask the question why the ausgezeichneten scales always have a typical location, depending on the species. Perhaps the elliptical outline of the eye can be held responsible. I could imagine that the neighbouring scales are being covered by the dorsal and ventral scale owing to the small curvature of this part of the eye and then the temporal and nasal scale should have to slide under the neighbouring scales due to the larger curvature. The variable outline of the eye would possibly determine the respective formation of the ausgezeichneten scales. It can also not be ruled out that possibly any forces, which are exerted by the eye muscles onto the eye, are effective in the formation of ausgezeichnete scales. In this sense, there could be a relationship between the eye muscles and the scales, but I would not like to agree with the thought that scales offer fixed points of attachment for the muscles. The B-type in detail is inexplicable to me, it is, however, striking that those bird species generally tend all the more so towards it the bigger their eyes are and thus the steeper the ring, hence the stronger the eye is pointed forwards.

This positioning towards the front in conjunction with the thereby stronger asymmetry of the eye might somehow bring about the B-type instead of the A-type. Thus the match between, for example, many owls and the common buzzard – which has a very steep ring - can be explained, whereas the honey buzzard has a shorter and flatter eye and therefore A-type. It should be looked at whether or not the skylark (B-type) has a relatively long-axis eye with a steep ring. A noticeable deviation from the just established rule exists in the fact that *Cypselus* exhibits a steep ring and A-type.

In conclusion, I would like to say the following. We know very little about the meaning of the sclerotic ring. Provided that we assume characteristics of adaptation of any kind of relationship, then we cannot reach clarity in any other way than by approaching this question experimentally. It then simply amounts to the task to eliminate the external effective factors that we suspect; but perhaps we will encounter big technical issues here. It cannot be ruled out that one would get further this way than through pure speculation.

**8. Summary**

The sclerotic ring of the birds’ eye is a structure of unmistakeable regularity.

**1. The scales**

The scale count varies between 11 and 17; 15 is the most frequent one. For each species a number is typical. Equally, each order or family is characterized by a typical scale count; for example the number 12 is the most common number for *Cuculiformes* (*Cuculidae* and *Psittacidae*), besides that there are deviations from 11 – 14; 11 is the most common number for *Columbidae*, the rare variations are 10 – 12; the number 13 can continuously be found in the *Alcidae,* the variations in the diurnal bird pf prey is bigger, it numbers 13 – 17, the most common figure is 15; the most common number for passerine birds is 14, variations are 13 and 15. More examples as per the above. Provided that two or three numbers stand out in particular in an order, as with *Coraciiformes* and *Charadriiformes* (see graphical images on pages 564 and 563), then those orders exist under noticeably differing living conditions – thus, under the *Charadriiformes*, the *Columbidae* have typically 11 scales, the *Alcidae* 13, the rest generally 15 scales - or their positioning in the system is still controversial.

**2. The ring**

The rooftile shaped kind of covering of the scales happens based on a specific structural design. Depending on the number of ausgezeichnete scales, two different types can be differentiated: A-type with 4 (at times even 6) ausgezeichneten scales and B-type with 2 ausgezeichneten scales (text fig. 1a, b). The plus-scales are positioned dorsally and ventrally, the minus-scale nasally and temporally. B-type exhibits a ventral plus-scale and a temporal minus-scale. The structural design is further characterized through the formation of ausgezeichnete scales. For each species, a specific structural design is typical; mostly each family has a specific structural design, for example *Anseridae* 1, 9; 5, 11. All of the passerine birds usually have 1, 8; 5, 10. There is a link between number of scales and formation of scales. The interlockings are individual deviations. A certain accumulation of interlockings can be observed in specific species (common buzzard), as well as on specific scales (7 X 8); also the kind of interlockings are recurring.

**3. Sclerotic ring and genealogical tree.**

Phylogenetic relationships due to the scale count:

A smaller scale count in phylogenetically old forms, for example *Opisthocomus, Cuculiformes, Coraciiformes* except owls and Cypselidae.

Contribution to controversial questions regarding family relations:

*Opisthocomus* seems to have family relations with the cuckoos; there are also relations of doves to the cuckoos. Related groups often have similar numbers in general.

Relatively biggest constancy in particularly higher developed orders (passerine birds).

**4. Histogenesis**

Time of formation of the ring: in a chicken approximately on the 12^th^ day of incubation. Process of formation: each scale has a bone centre (cf. cranial bone) in H. MÜLLER’s blastema. The scales grow towards each other and finally cover each other or at times interlockings happen. This state is probably reached in 4 days in a chicken.

**5. Function**

A support and protective function is an undisputed fact. The ring can, in a sense, be seen as an extension of the orbit. The meaning of segmentation: there is primarily a link between segmentation and growth. Due to the fact that the ring has the function of a capsule, it must be able to comfortably follow the changes in proportion to its surroundings and for this, the segmented ring offers the best guarantee (cranial bone). The number of scales is probably to be seen as an organisational characteristic. The elliptical outline of the eye is likely to be held responsible for the formation of the scales in an A-type; in the B-type one can probably add the long-axis of the eyes concerned as a cause.

______________

I would like to genially thank Professor Dr. V. FRANZ for the suggestion to this work and the many pieces of advice that he gave me. I am much obliged to Dr. L. PLATE for the granting of a position in the institute as well as his continued interest in my work, to Professor Dr. H. HOFFMANN for plenty of advice and the museum in Senkenberg for granting ample and valuable material.

______________
